# Precise and *In Vivo*-Compatible Spatial Proteomics via Bioluminescence-Triggered Photocatalytic Proximity Labeling

**DOI:** 10.1021/acscentsci.5c00520

**Published:** 2025-07-30

**Authors:** Xuege Sun, Yanling Zhang, Wenjie Lu, Hongyang Guo, Guodong He, Siyuan Luo, Haodong Guo, Zijuan Zhang, Wenjing Wang, Ling Chu, Xiangyu Liu, Wei Qin

**Affiliations:** † State Key Laboratory of Membrane Biology, Tsinghua University, Beijing 100084, China; ‡ School of Pharmaceutical Sciences, Tsinghua University, Beijing 100084, China; § Tsinghua-Peking Center for Life Sciences, Tsinghua University, Beijing 100084, China; ∥ MOE Key Laboratory of Bioorganic Phosphorus Chemistry & Chemical Biology, Tsinghua University, Beijing 100084, China; ⊥ Beijing Frontier Research Center for Biological Structure, Tsinghua University, Beijing 100084, China; ∇ Institute of Medicinal Plant Development, Chinese Academy of Medical Sciences and Peking Union Medical College, Beijing 100193, China

## Abstract

Protein function is closely tied to its localization and interactions, which can be mapped using proximity labeling (PL). Traditional PL methods, such as peroxidases and biotin ligases, suffer from toxicity or high background. While visible-light-triggered photocatalytic labeling offers great potential, it is limited by light-induced background and restricted *in vivo* applications. Here we present BRET-ID, an *in vivo*-compatible PL technology for precise mapping of membraneless organelles and transient protein–protein interactions with subminute temporal resolution. BRET-ID combines a genetically encoded photocatalyst and NanoLuc luciferase, locally generating blue light to activate the photocatalyst via bioluminescence resonance energy transfer (BRET). This activation produces singlet oxygen, which oxidizes nearby proteins for analysis with a streamlined chemoproteomic workflow. BRET-ID enables precise mapping of ER membrane proteins, exhibiting high spatial specificity. Leveraging its high temporal resolution, BRET-ID provides 1 min snapshots of dynamic GPCR interactions during ligand-induced endocytosis. Additionally, BRET-ID identifies G3BP1-interacting proteins in arsenite-stressed cells and tumor xenografts, uncovering novel stress granule components, including the mTORC2 subunit RICTOR. BRET-ID serves as a powerful genetically encoded tool for studying protein localization and molecular interactions in living organisms.

## Graphical abstract

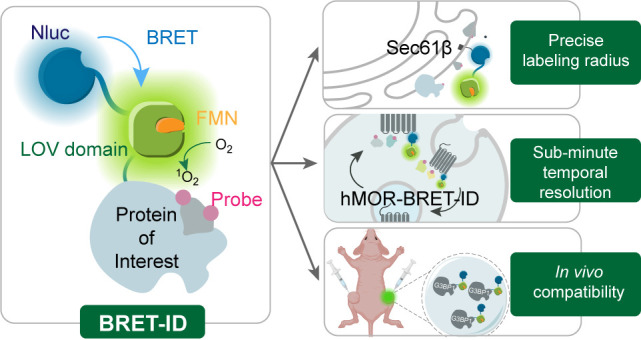

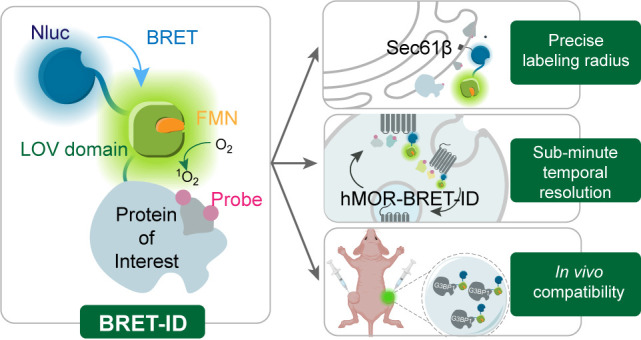

Social networks between proteins, including their close partnerships and interactions within specific subcellular communities, form the fundamental basis of cellular processes. Mammalian cells are highly compartmentalized, with proteins dynamically segregating into defined assemblies.^1,2^ For example, numerous proteins are recruited into biomolecular condensates, such as membraneless organelles like stress granules (SGs) and P bodies.^3,4^ The assembly of SGs depends on both the stable interactions of proteins in the “core” and the dynamic associations of proteins in the “shell”.^5^ Mass spectrometry-based proteomics has largely facilitated the understanding of protein components and interactions through fractionation-based approaches like density gradient ultracentrifugation and co-immunoprecipitation.^6,7^

In recent years, proximity labeling (PL) techniques, including APEX2,^8,9^ TurboID,^10^ and miniSOG,^11–14^ have advanced the mapping of subcellular proteomes and molecular interactions. APEX2, a peroxidase that labels nearby proteins through phenoxyl radical formation upon H_2_O_2_ addition, is limited by its toxicity, which can interfere with redox-sensitive pathways,^15^ making it unsuitable for use in living animals. On the other hand, biotin ligases like BioID^16,17^ and TurboID^10,18–20^ can be used *in vivo*, but high tissue biotin concentrations lead to significant background labeling, complicating the identification of true positives. MiniSOG and its variants, genetically encoded photosensitizers, generate singlet oxygen via endogenous flavin mononucleotide (FMN) under blue light, oxidizing and labeling nearby biomolecules.^11–14,21^ Small molecule photosensitizers, including metal complexes^100,22^ and fluorescent dyes^101,23^ have also been developed and can be targeted to specific proteins through haloalkane modification and attachment to a HaloTag-fused protein.^105–26^ However, the limited penetration depth of blue light^27^ and the potential activation of endogenous photosensitizers^28^ pose challenges, confining these photocatalytic methods primarily to cultured cells and leading to high background and nonspecific identifications.^29^

The labeling radius of PL methods is determined by both the half-life of the reactive species and the density of surrounding biomolecules, making it highly context-dependent, but generally within the range of 1–20 nm in living cells.^30–32^ However, labeling is often not strictly confined to specific assemblies and can diffuse into other subcellular regions, leading to false positives. For example, PL enzymes anchored to the ER membrane (ERM) may label not only ER-resident proteins but also a large number of cytosolic proteins.^10,12,33^ To exclude these cytosolic bystanders, a ratiometric analysis against a cytosol-localized PL experiment, known as a spatial reference, is used to identify proteins more significantly enriched by the ER-targeted labeling. This spatial reference strategy has become a standard practice to enhance the specificity of PL in mapping components of open organelles^24,34,35^ (i.e., those not enclosed by membranes) and in resolving specific protein–protein interactions (PPIs).^36–38^ However, such ratiometric analysis may inadvertently exclude dual-localized proteins, reducing detection of sensitivity. Additionally, selecting an appropriate spatial reference requires knowledge of the bait’s subcellular localization, which can be challenging when the bait is highly dynamic or localizes to multiple sites.

In this study, we introduce BRET-ID, a novel PL technology that enables nontoxic protein labeling with high spatiotemporal precision. BRET-ID employs a fusion or chimeric protein consisting of a bright luciferase^39^ and a genetically encoded photosensitizer. Upon the addition of a luciferase substrate, local blue light is emitted, activating the photosensitizer through bioluminescence resonance energy transfer (BRET).^40^ This process eliminates the need for exogenous blue light, thereby avoiding nonspecific labeling caused by endogenous photosensitizers. Given the widespread use of luciferase and its substrates in animal experiments, BRET-ID offers a nontoxic labeling method suitable for *in vivo* applications. We demonstrate that BRET-ID generates a highly confined labeling radius, enabling the precise identification of subcellular proteomes and PPIs. Proteomic data sets for the ERM were generated, showing significantly higher spatial specificity compared to blue-light-triggered labeling. With its high temporal resolution (1 min labeling), BRET-ID was used to capture snapshots of GPCR-proximal proteins during GPCR endocytosis, identifying ZYG11B and Septin7 as novel GPCR-associated proteins. We also used BRET-ID to identify G3BP1-interacting proteins under both basal and arsenite-induced oxidative stress conditions, uncovering previously unrecognized SG proteins, such as RICTOR and TLK1. Finally, we highlight the versatility of BRET-ID for *in vivo* labeling and present the first proteomic map of SG proteomes in living mice, revealing novel G3BP1-interacting proteins specifically discovered in tumor xenografts.

## Results and Discussion

### Development and Validation of BRET-ID

To develop BRET-ID, we initially explored a chemogenetic approach using a HaloTag-attached photosensitizer to map neighboring proteins of HaloTag-fused proteins.^41^ These photosensitizers generate singlet oxygen species under light irradiation to oxidize surrounding proteins. These proximal oxidized proteins can be labeled with amine-directed probes, such as alkyne-aniline. While this method has been successfully implemented with various haloalkane-modified photosensitizers,^105–26^ it has a significant limitation: excess photosensitizer, including endogenous photosensitizers, can also generate protein labeling upon light irradiation (Figure 1a). To overcome this, we hypothesized that the HaloTag-attached photosensitizer could be activated by the light emitted from an associated luciferase through a BRET mechanism, eliminating the need for exogenous light (Figure 1b). We selected a chimera of NanoLuc and HaloTag (cpNluc-HT), with circularly permuted NanoLuc inserted into HaloTag, a system previously used for imaging with tunable colors^42^ (Figure S1a). Since the emission maximum of NanoLuc is 460 nm, we screened a range of blue light-activatable photocatalysts to assess their photocatalytic labeling efficiency with NanoLuc (Figure S1b). HEK293T cells expressing NanoLuc were incubated with various photosensitizers and alkyne-aniline, followed by the addition of furimazine to trigger labeling. The cell lysates were reacted with azide-rhodamine via Click reaction (CuAAc, Cu (I)-catalyzed azide–alkyne 1,3-dipolar cycloaddition), followed by in-gel fluorescence detection. This comparison led to the discovery of hematoporphyrin monomethyl ether (HMME),^43^ a photodynamic therapy photosensitizer that outperformed other photosensitizers like eosin Y and Ru+ complex in NanoLuc-triggered photocatalytic labeling (Figure S1c).

**Figure 1:**
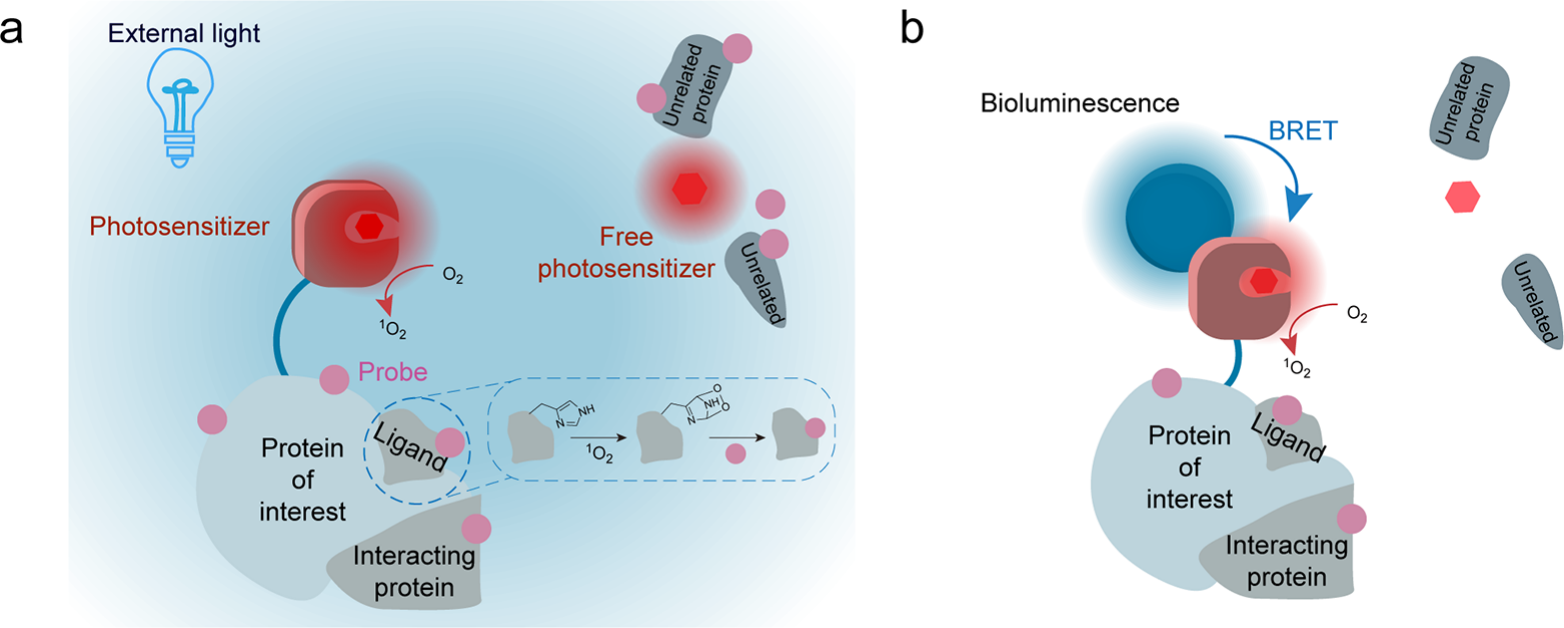
Design of BRET-ID. (a) Schematic of traditional photocatalytic PL. Blue light irradiation not only activates the photosensitizers attached to the HaloTag or the genetically encoded photosensitizers (e.g., miniSOG) but also triggers free photosensitizers, leading to nonspecific labeling. (b) Schematic of the BRET-based PL presented in this study. The bioluminescence emitted by the luciferase specifically activates the associated photocatalyst via the BRET mechanism. Free, unbound photocatalysts remain inactive.

We then synthesized a chloroalkane-modified HMME (CA-HMME) (Figure S1d) and incubated it with HEK293T cells expressing the cpNluc-HT chimera for 1 h, followed by the addition of furimazine, the NanoLuc substrate, and alkyne-aniline, the labeling probe, for 45 min (Figure S1e). Promiscuous proteome labeling was observed in the labeled sample, whereas control samples lacking either furimazine, the enzyme, or the probe showed no protein labeling (Figure S1f). To confirm that the labeling originated from the enzyme-associated HMME, HEK293T cells expressing the cpNluc-HT chimera were also incubated with unmodified HMME, resulting in significantly lower proteome labeling (Figure S1f). We also conducted HMME-mediated labeling with blue light irradiation at 200 mW/cm^2^ for 5 min and found that the labeling was not dependent on HaloTag (Figure S1g). Cells lacking HaloTag or treated with unmodified HMME showed similar levels of labeling, as free HMME could not be fully washed out and continued to label proteins upon light exposure. These experiments demonstrate that NanoLuc can activate its associated photosensitizer through the BRET mechanism.

### Development of Genetically Encoded BRET-ID

To fully develop BRET-ID as a genetically encoded tool, we replaced HaloTag with the Light-Oxygen-Voltage (LOV) domain, which uses endogenous FMN as the photocatalyst (Figure 2a). LOV domains, such as miniSOG, efficiently generate singlet oxygen under blue light without the need for exogenous photocatalysts and have been successfully used in PL to resolve PPIs and map subcellular proteomes.^11–14^ To create a LOV-based BRET-ID, we engineered fusion constructs with different LOV domains and various linker strategies, evaluating their labeling efficiency (Figure 2b). Through systematic optimization, we identified NanoLuc-SOPP3—a fusion protein of NanoLuc and SOPP3 ^44^ linked by a rigid 15-amino-acid linker—as the most efficient BRET-ID version (Figures 2c and S2a,b). This rigid linker might stably maintain an optimal donor–acceptor distance, a critical parameter for efficient energy transfer. Based on these findings, this construct served as our lead candidate for subsequent characterization. We also compared different probes, and alkyne-aniline demonstrates superior labeling efficiency over alkyne-phenol for detecting oxidized proteins resulting from SOPP3-generated singlet oxygen (Figure S2c). The addition of furimazine to HEK293T cells expressing NanoLuc-SOPP3 resulted in clear proteome labeling, whereas cells expressing NanoLuc alone showed negligible labeling (Figure 2d). Although blue light-induced labeling of NanoLuc-SOPP3 was significantly higher, it also led to substantial background labeling in cells lacking the LOV domain. We confirmed that this fusion protein exhibits similar photocatalytic labeling efficiency to SOPP3, indicating that NanoLuc does not interfere with SOPP3′s activity (Figure S2d). We also tested 1-s blue light irradiation, as its photon emission is comparable to NanoLuc’s emission level. However, no obvious labeling was detected with this light energy (Figure S2e), suggesting that the light emitted by NanoLuc is efficiently transferred to the LOV domain. To further confirm the BRET effect between NanoLuc and SOPP3, we measured the relative fluorescence intensity of NanoLuc-SOPP3 after furimazine addition. Unlike NanoLuc, which emits blue light, the emission of NanoLuc-SOPP3 shifted to a green spectrum, as SOPP3 is also a green fluorescent protein^44^ (Figure 2e).

**Figure 2:**
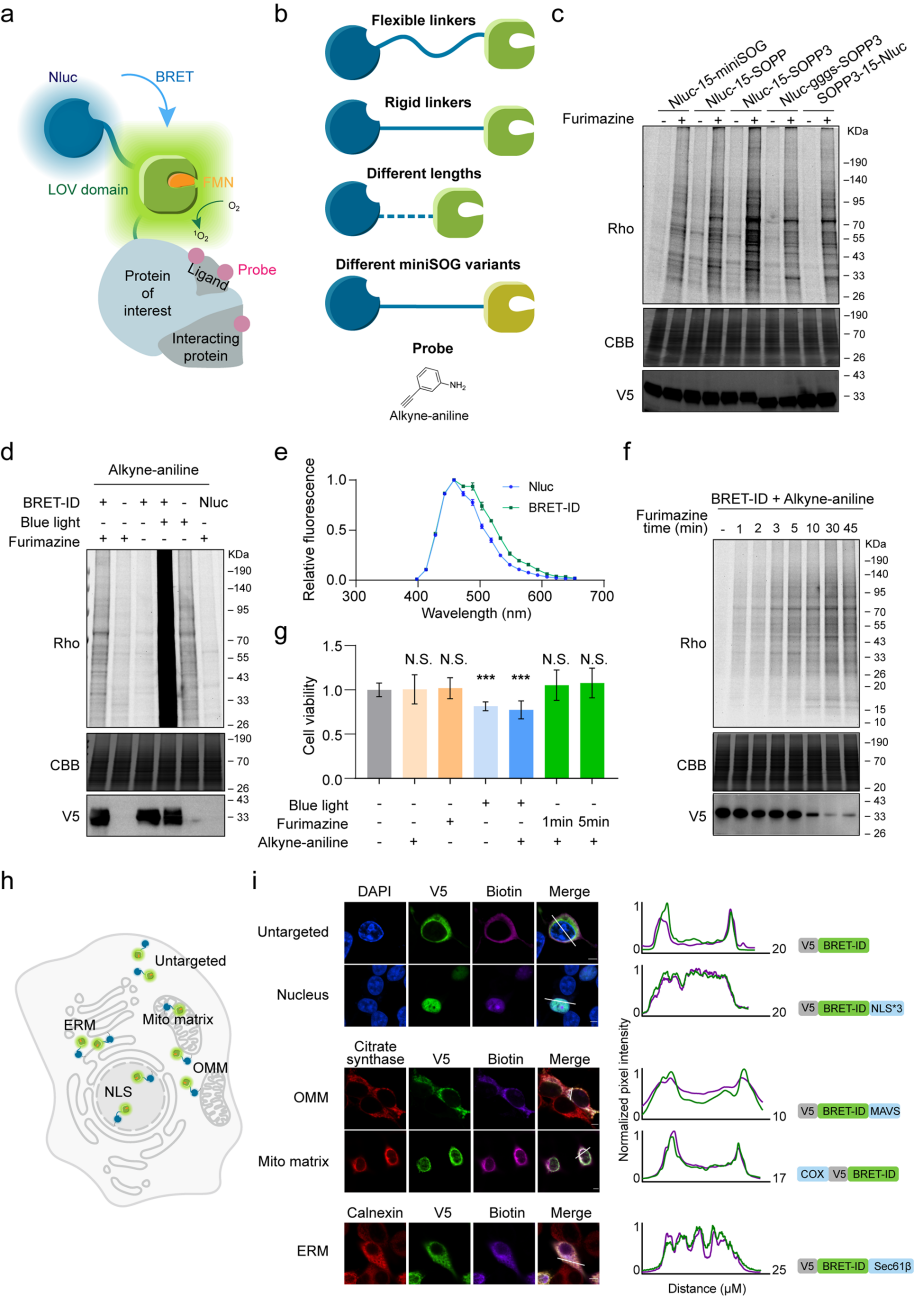
Development of the genetically encoded BRET-ID system. (a) The design of the genetically encoded BRET-ID fusion protein consists of a NanoLuc luciferase and the LOV domain. The bioluminescence emitted by the luciferase specifically activates the FMN-containing LOV domain through the BRET mechanism. (b) Schematic of the optimization of the NanoLuc-LOV fusion protein for efficient PL. (c) The NanoLuc-SOPP3 fusion protein, linked by a rigid 15-amino acid linker, exhibits the highest labeling efficiency. HEK293T cells were transfected with various fusion constructs and treated with 1 mM alkyne-aniline for 15 min, followed by cotreatment with 75 μM furimazine and 1 mM alkyne-aniline for an additional 45 min. (d) In-gel fluorescence scanning of the genetically encoded BRET-ID (NanoLuc-15-SOPP3) labeling following furimazine addition or blue light irradiation. HEK293T cells expressing the BRET-ID tag were subjected to labeling under either furimazine activation (75 μM for 45 min) or blue light activation (200 mW/cm^2^ for 10 min). (e) Quantification of fluorescence intensity for encoded BRET-ID and NanoLuc after furimazine addition. HEK293T cells expressing either the BRET-ID tag or NanoLuc were treated with 7.5 μM furimazine and 20 μM riboflavin. The fluorescence intensity was measured at various wavelengths using a plate reader immediately after the treatment. (f) Effect of furimazine treatment duration on BRET-ID labeling efficiency. HEK293T cells expressing the BRET tag were preincubated with 1 mM alkyne-aniline for 15 min, followed by cotreatment with 75 μM furimazine for varying durations. (g) Impact of blue light and furimazine treatment on cell viability. HEK293T cells expressing the BRET-ID tag were treated with 75 μM furimazine or blue light with or without 1 mM alkyne-aniline. The cell viability was determined by the MTS assay. The error bars show mean ± SD. ***, *p* < 0.001; N.S., not significant (Student’s *t* test). (h) Schematic of BRET-ID fusion proteins targeting different subcellular compartments, including the cytosol, nucleus, mitochondrial matrix, outer mitochondrial membrane (OMM), and ER membrane (ERM). (i) Confocal fluorescence imaging of BRET-ID labeling in various subcellular locations. HEK293T cells were transfected with the corresponding BRET-ID constructs for 24 h, followed by furimazine-based labeling initiation. Cells were fixed and labeled with azide-biotin, followed by staining with streptavidin-AF647 for visualization of labeled proteins. Anti-V5 staining indicates enzyme expression. Citrate synthase marks the mitochondria, and calnexin marks the ER. Scale bars, 5 μm. White lines indicate where line plots were generated. Average intensity of biotinylation and V5 staining was quantified.

Next, we evaluated the temporal resolution of NanoLuc-SOPP3 labeling by treating cells with furimazine for varying durations. Remarkably, just 1 min of furimazine exposure resulted in effective proteome labeling, with labeling nearly saturating at 5 min, even though NanoLuc can continue emitting blue light for up to 45 min (Figure 2f). To rule out the possibility that local FMN depletion contributes to the loss of BRET-ID activity, we supplemented BRET-ID-expressing cells with 150 μM riboflavin, a precursor of FMN. However, this treatment failed to restore activity after a 5 or 10 min incubation with furimazine (Figure S2f). This suggests a potential “suicide” mechanism, where prolonged blue light activation leads to the inactivation of the LOV domain. This aligns with previous studies indicating that singlet oxygen generated by the LOV domain can oxidize its own electron-rich residues,^45,46^ potentially leading to enzyme inactivation following alkyne-aniline conjugation. To confirm this mechanism, we performed a sequential labeling experiment: cells were first labeled with alkyne-aniline for 5 min, followed by biotin-aniline labeling for an additional 5 min. Minimal biotinylation was detected during the second 5 min, whereas direct biotin-aniline labeling during the first 5 min showed much stronger biotinylation (Figure S2g). This indicates that BRET-ID is intrinsically self-limiting, eliminating the need for quenching reagents or extensive washes, making it ideal for pulse-chase experiments. We also optimized the concentration of furimazine and alkyne-aniline for BRET-ID labeling (Figure S2h). Under the optimized condition with 75 μM furimazine and 1 mM alkyne-aniline, we conducted a cell viability assay to assess the toxicity of BRET-ID labeling and found no significant toxicity after 1 or 5 min of furimazine induction (Figure 2g). In contrast, photocatalytic labeling triggered by blue light caused significant toxicity, confirming that BRET-ID is nontoxic.

To assess the spatial specificity of BRET-ID, HEK293T cells were transfected with NanoLuc-SOPP3 targeted to specific organelles—such as the mitochondrial matrix, outer mitochondrial membrane, ERM, and nucleus—using organelle-targeting sequences, followed by confocal microscopy imaging (Figure 2h). We found that NanoLuc-SOPP3 could be specifically targeted to all four compartments, exhibiting furimazine-dependent labeling with high spatial specificity (Figure 2i). Since the ERM is an open compartment facing the cytosol, traditional PL methods like APEX2 and TurboID often result in diffuse labeling, a phenomenon we also observed with blue-light-triggered labeling (Figure S2i). In contrast, BRET-ID labeling induced by furimazine was strictly confined to the enzyme’s localization. This suggests that BRET-ID offers high spatial resolution, likely because it generates a smaller amount of singlet oxygen, which primarily oxidizes the nearest neighbors. We hereby designate the NanoLuc-SOPP3 fusion protein as the BRET-ID tag.

### Precise Mapping of ER Membrane Proteins by BRET-ID

We aimed to benchmark BRET-ID by performing a proteomic profiling of the local proteome at the ERM, a well-established compartment used to assess the spatial specificity of PL methods^10,12,33^ (Figure 3a). HEK293T cells expressing BRET-ID-ERM were treated with 1 mM alkyne-aniline for 15 min, followed by the treatment of 75 μM furimazine for 1 min. A negative control was included in which the furimazine treatment was omitted, along with a spatial reference using untargeted BRET-ID to nonspecifically label all cytosolic proteins. We also conducted the same experiments under blue light (BL) irradiation at 200 mW/cm^2^ for 10 min. Additionally, we performed blue light irradiation on untransfected cells to differentiate background labeling caused by endogenous free photocatalysts, such as FMN (Figure 3b). After cell lysis, click reaction with azide-biotin was performed. Streptavidin blotting confirmed both BRET-ID- and BL-triggered labeling, as well as the successful enrichment of biotinylated proteins (Figure S3a). Biotinylated proteins were captured using streptavidin beads and subjected to on-beads trypsin digestion to release peptides. To quantify the enriched proteins across different conditions and replicates, we employed data-independent acquisition (DIA)-based liquid chromatography-tandem mass spectrometry^47,48^ (LC-MS/MS). Unlike data-dependent acquisition, which is commonly used in previous PL studies, DIA offers more comprehensive coverage of precursor peptide ions, regardless of their intensities, and provides more accurate quantitation with improved reproducibility. The DIA-based data was analyzed using DIA-NN software,^49^ allowing for simultaneous quantification of peptide intensities across different samples. Ultimately, 5312 and 5402 proteins were quantified across three biological replicates for BRET- and BL-dependent labeling, respectively, with high correlation observed across replicates (Figure S3b).

**Figure 3:**
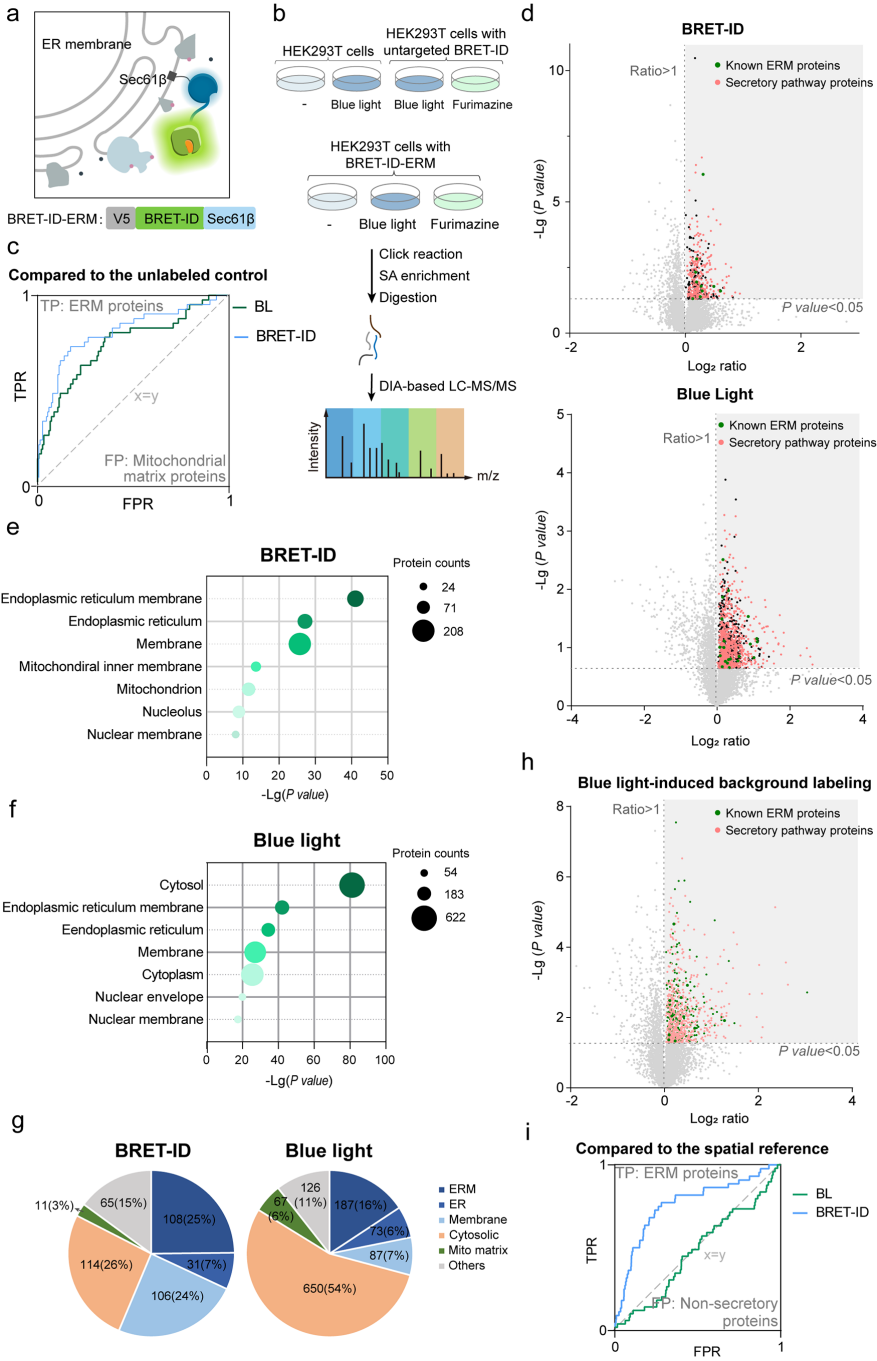
Precise mapping of ER membrane proteins by BRET-ID. (a) Schematic of ERM-targeted BRET-ID labeling and the design of DIA-based proteomics for both BRET- and blue-light-triggered PL. (b) Design of DIA-based proteomics for mapping ERM proteins using BRET-ID. (c) Receiver operating characteristic (ROC) curves for BRET- and blue-light-triggered PL on ERM. Proteins are ranked in descending order based on enrichment ratios against unlabeled controls. True positives are known ER membrane proteins, while false positives are annotated mitochondrial matrix proteins. (d) Volcano plots showing the enrichment of labeled proteins in BRET- and blue-light-triggered PL on ERM. Proteins that are significantly enriched in the labeled samples (*p* < 0.05) are highlighted, with known ER membrane proteins in green and secretory proteins in pink. (e) Gene Ontology (GO) cellular component analysis of BRET-ID-enriched proteins. (f) GO cellular component analysis of proteins enriched by blue-light-triggered labeling. (g) Percentages of enriched proteins with various subcellular localizations for BRET- or blue-light-triggered ERM labeling. (h) Volcano plots showing the enrichment of blue light-induced protein labeling in untransfected cells. (i) ROC curves comparing ERM-targeted labeling with cytosolic spatial reference labeling for both BRET- and blue-light-triggered PL. True positives are known ER membrane proteins, while false positives are nonsecretory proteins.

We began by performing receiver operating characteristic (ROC) analysis to compare BRET-based and BL-induced ERM labeling, a commonly used method for evaluating the specificity of ERM labeling with PL techniques.^10,12,33^ A *bona fide* ERM protein should show a high enrichment ratio compared to the nonlabeled control, while a false positive—such as an endogenously biotinylated protein or a nonspecific bead binder—would exhibit a low enrichment ratio, as it would be captured similarly in both conditions. We ranked the quantified proteins by descending mean enrichment ratio and plotted the ROC curve, using known ERM proteins as true positives and mitochondrial matrix proteins as false positives. This curve illustrates the true positive rate (TPR) against the false positive rate (FPR) for detected proteins. We found that both methods significantly enriched ERM proteins, with BRET generally showing higher TPRs compared to BL-based labeling (Figure 3c). Proteins that were significantly enriched (*p* < 0.05) were retained, resulting in sets of 435 BRET-labeled and 1190 BL-labeled proteins (Figure 3d and Table S1). Gene Ontology (GO) analysis of BRET-labeled proteins revealed significant enrichment of terms related to the ERM and membranes in contact with it, such as the nuclear and Golgi membranes (Figure 3e). In contrast, the most enriched GO term for BL-labeled proteins was the cytosol (with 333 cytosolic proteins labeled), although ERM-related terms were also enriched (Figure 3f). Indeed, a significantly larger portion of BL-labeled proteins were cytosolic soluble proteins, while most BRET-labeled proteins were previously annotated as ERM- or membrane-resident proteins (Figure 3g). This suggests that BL-based labeling generates a broader labeling radius, potentially due to the higher levels of reactive intermediates produced by the LOV domain or the nonspecific labeling caused by the diffusive endogenous photocatalysts.

Indeed, blue light irradiation induces widespread proteome labeling in untransfected cells, resulting in the identification of 423 significantly enriched proteins (*p* < 0.05) (Figure 3h and Table S2). These proteins are enriched in various compartments, including the cytosol and membrane-enclosed organelles, suggesting that this background labeling may interfere with PL-based mapping in those organelles (Figure S3c). Fluorescent imaging further revealed pervasive light-induced background labeling throughout the cells (Figure S3d).

In previous attempts to map ERM proteins using classic PL methods, such as APEX2 ^33^ and TurboID,^10^ comparisons with nonlabeled controls alone led to the identification of many cytosolic proteins (Figure S4a). A common strategy to improve ERM specificity is to additionally compare against a cytosolic spatial reference. This ratiometric analysis assumes that *bona fide* ERM proteins will be preferentially labeled by ERM-targeted labeling over the spatial reference, while cytosolic bystanders will be more labeled by the cytosol-resident labeling. However, as discussed in previous studies, this extra comparison can result in the loss of many true positives, particularly dual-localized proteins, which decreases sensitivity.^50–52^ Therefore, we further conducted ROC analysis on the comparative ratio between ERM-targeted and cytosol-resident labeling, using known ERM proteins as true positives and nonsecretory proteins as false positives. Our results showed that the BRET-based approach preferentially enriched known ERM proteins over nonsecretory proteins, whereas the BL-based approach failed to do so (Figure 3i). After applying the spatial reference filter, the BL-based method yielded a more specific list of 190 ERM proteins (Figure S4b and Table S2). However, this additional filtering step also excluded many known ERM proteins, such as calumenin and reticulocalbin-1, consistent with previous observations (Figure S4c). Applying the spatial reference filter to the BRET-ID data resulted in a significantly smaller list of only 77 proteins, with numerous known ERM proteins excluded (Figure S4d). Critically, however, we observed no significant improvement in ERM specificity following this spatial reference comparison (Figure S4e). Given that the BRET-ID-ERM data set prior to spatial reference comparison already exhibits high specificity, we have used this larger list for subsequent analyses. Collectively, these results suggest that BRET-ID provides highly precise labeling and enables spatial proteomic mapping of open compartments.

### Mapping Dynamic GPCR Interactomes by BRET-ID

After validating the spatial specificity of BRET-ID, we next aimed to evaluate its temporal resolution for mapping dynamic PPIs, a key area where PL methods are often utilized. Ratiometric analysis based on spatial references is commonly used to identify proteins in close proximity to the bait, rather than those located within the same compartment. Given that BRET-ID offers high temporal resolution, with labeling occurring in as little as 1 min, we sought to apply it to map the transient interactions of G protein-coupled receptors (GPCRs) in response to ligand stimulation. While APEX2 has been previously used to capture 1 min snapshots of GPCRs during ligand-induced endocytosis,^36–38^ its application often requires complex spatial references due to inherent spatial variations and exogenous H_2_O_2_ treatment might interfere with GPCR dynamics through oxidative stresses. Since APEX2-generated phenoxyl radicals have a relatively long half-life, we hypothesized that BRET-ID could provide a more localized labeling radius without relying on spatial references. To test this, we fused the human μ-opioid receptor (hMOR)^53^ with the BRET-ID sequence and transfected the construct into HEK293T cells. hMOR can be activated by the opioid peptide agonist DAMGO,^54^ which induces endocytosis and allows for the labeling of nearby interacting proteins by BRET-ID (Figure 4a). We first used a split-luciferase-based cAMP biosensor to measure DAMGO-induced inhibition of cAMP.^55^ The cells were treated with varying concentrations of DAMGO, and we confirmed that tagging with the BRET-ID sequence did not interfere with hMOR activation (Figure 4b).

**Figure 4:**
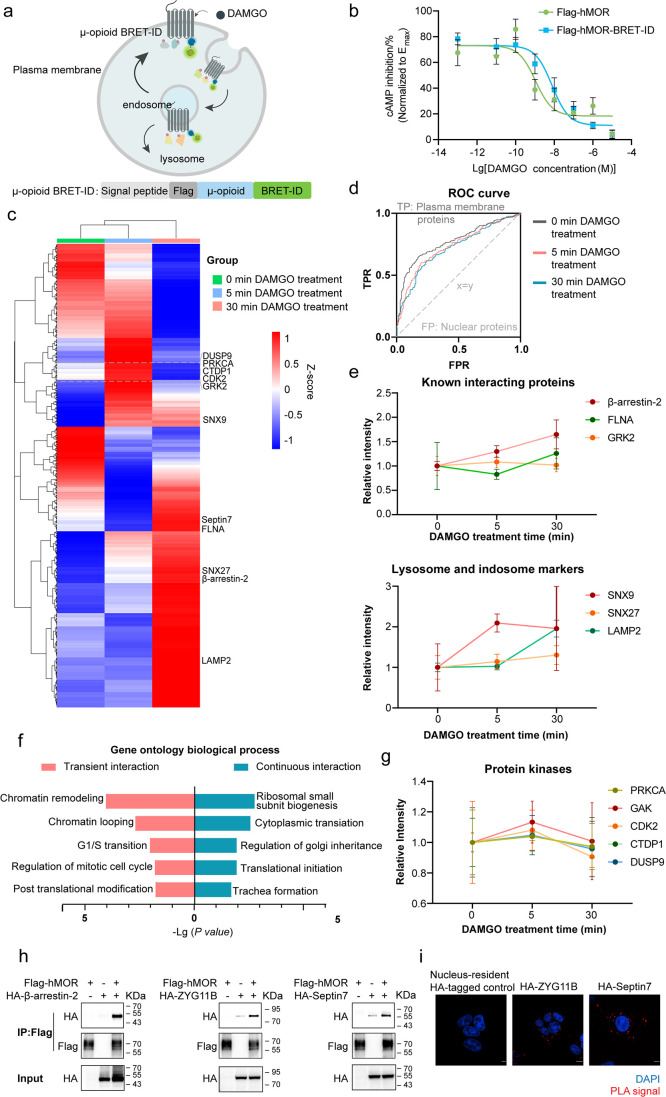
Dynamic interactome mapping of human μ-opioid receptor using BRET-ID. (a) Schematic of BRET-ID labeling to map hMOR-proximal proteins during DAMGO-induced endocytosis. (b) Impact of the BRET-ID tag on hMOR activity. HEK293T cells were transfected with Flag-hMOR or Flag-hMOR-BRET-ID, along with a split-luciferase based cAMP biosensor. Medium was replaced with CO_2_-independent medium containing 150 μg/mL luciferin and incubated for 1 h at 37 °C, followed by another 1 h at room temperature. The baseline of luminescence was measured before drug stimulation. To activate hMOR, cells were treated with different concentrations of DAMGO and incubated at room temperature for 15 min. Then a final concentration of 200 nM isoprenaline was added per well to stimulate endogenous cAMP via β2-adrenergic-Gs activation. Fluorescence intensity was measured using a plate reader. (c) Heat map showing DAMGO-dependent changes in the proximal proteomes of the hMOR. Proteins that are significantly enriched in at least one time point were subjected to the clustering analysis. (d) ROC curves of hMOR-BRET-ID labeling at different DAMGO treatment time points. True positives are known plasma membrane proteins, while false positives are nuclear proteins. (e) Relative intensities of known hMOR-interacting proteins and lysosome/endosome markers at different DAMGO treatment time points. (f) GO biological process analysis of transient (left) and continuous (right) DAMGO-induced hMOR-proximal proteins. (g) Relative intensities of protein kinases and phosphatases involved in chromatin remodeling, which were annotated as DAMGO-dependent transient interactors. In (b), (e), and (g), the error bars show mean ± SD. (h) Validation of ZYG11B and Septin7 as hMOR-interacting proteins by co-immunoprecipitation. HEK293T cells were transfected with Flag-tagged hMOR, along with HA-tagged interacting proteins (e.g., β-arrestin-2, ZYG11B, and Septin7). The cell lysates were subjected to immunoprecipitation using an anti-Flag antibody, and the eluates were analyzed by Western blotting with an anti-HA antibody to detect the corresponding HA-tagged interacting proteins. (i) Validation of ZYG11B and Septin7 as hMOR-interacting proteins by proximity ligation assay (PLA). HEK293T cells were transfected with Flag-tagged hMOR and HA-tagged interacting proteins (e.g., ARRB2, ZYG11B, and Septin7). The cells were then fixed and subjected to PLA imaging. A nucleus-localized HA-tagged HaloTag was used as a negative control. Scale bars, 5 μm.

Next, we evaluated hMOR-BRET-ID labeling and found that 1 min furimazine treatment resulted in efficient biotin-aniline labeling (Figure S5a). Based on this, we performed 1 min hMOR-BRET-ID labeling with DAMGO stimulation for 0, 5, and 30 min to identify hMOR-proximal proteins during its ligand-induced trafficking (Figure S5b). The labeling was confirmed by streptavidin blotting (Figure S5c), followed by DIA-based LC-MS/MS analysis (Figure S5d and Table S3). The proteomic data revealed distinct hMOR interactomes before and after ligand stimulation (Figure 4c). Proteins labeled prior to DAMGO treatment were predominantly enriched in plasma membrane proteins (Figure 4d), while after DAMGO treatment, there was increased labeling of endolysosomal markers, as well as known hMOR-interacting proteins such as β-arrestin-2 and GRK2 (Figure 4e). Among proteins that exhibited stronger interactions with hMOR after 5 min of DAMGO treatment, 65 proteins maintained their interaction, while 105 proteins showed a decrease in interaction after 30 min of DAMGO treatment. These proteins were enriched in distinct biological processes, with transient interactors being more enriched in chromatin remodeling, and prolonged interactors showing greater enrichment in cytosolic translation (Figure 4f). For example, we identified several protein kinases and phosphatases involved in chromatin remodeling that were annotated as DAMGO-dependent transient interactors, including protein kinase C alpha (PRKCA), cyclin G-associated kinase (GAK), cyclin-dependent kinase 2 (CDK2), CTD phosphatase subunit 1 (CTDP1), and dual specificity phosphatase 9 (DUSP9) (Figure 4g). Moreover, protein clusters that showed decreased interactions with hMOR after 5 min of DAMGO treatment were also enriched in distinct biological processes (Figure S5e).

To validate novel hMOR-proximal proteins, we performed co-immunoprecipitation of hMOR, followed by Western blot detection of ZYG11B and Septin7, two novel interactors. Both proteins were significantly enriched in the hMOR co-IP, though to a lesser extent than the well-characterized hMOR-interacting protein β-arrestin-2 (Figure 4h). Additionally, we performed a proximity ligation assay (PLA) in HEK293T cells cotransfected with Flag-tagged hMOR and HA-tagged selected proteins. The PLA revealed a significant fluorescent signal between these proteins and hMOR, in contrast to the nucleus-localized HA-tagged control (Figure 4i). These orthogonal approaches confirm the reliability of the novel hMOR-interacting proteins identified by BRET-ID and underscore the potential of BRET-ID for studying dynamic protein interactomes with unprecedented spatiotemporal resolution.

### Precise Proteomic Profiling of Stress Granules by BRET-ID

After separately validating the spatial and temporal resolution of BRET-ID, we next evaluated its ability to map stress granules (SGs),^56^ membraneless organelles known for their highly dynamic molecular interactions in both space and time. To do this, we established a stable HEK293T cell line expressing a BRET construct fused to G3BP1 (Ras GTPase-activating protein-binding protein 1), a core component of SGs^57^ (Figure 5a). Western blot analysis revealed that the expression level of G3BP1-BRET-ID was 1.2-fold of the endogenous G3BP1 level (Figure S6a). The cells were then treated with 0.5 mM sodium arsenite for 60 min to induce SG formation, followed by BRET-ID labeling with 75 μM furimazine for 5 min. Immunofluorescence imaging showed that the expression and labeling of G3BP1-BRET-ID strongly overlapped with the SG marker protein FXR1 after arsenite treatment (Figure 5b). In contrast, under basal conditions, the labeling was dispersed throughout the cells. Additionally, we confirmed that the BRET-ID workflow did not induce granule formation in the absence of sodium arsenite, indicating minimal interference with the native cellular state (Figure S6b). Building on these results, we proceeded to enrich the labeled proteins and perform DIA-based proteomic analysis, which demonstrated excellent reproducibility (Figures 5c and S6c).

**Figure 5:**
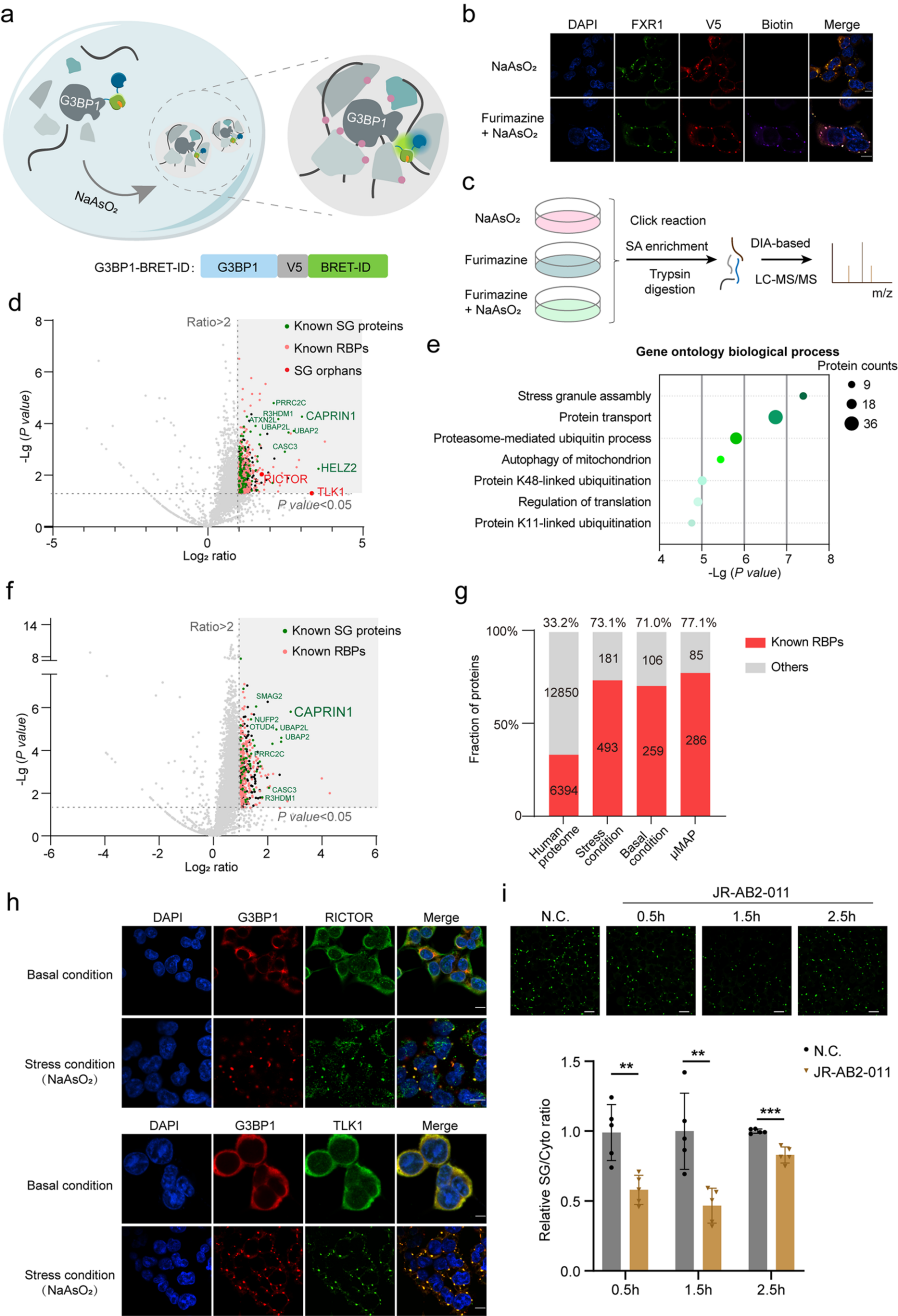
Precise proteomic profiling of SGs using BRET-ID. (a) Schematic of SG-targeted BRET-ID labeling. (b) Confocal fluorescence imaging of BRET-ID labeling in SGs. HEK293T cells stably expressing G3BP1-BRET-ID were treated with 500 μM sodium arsenite for 1 h, followed by furimazine-based BRET-ID labeling and imaging. Streptavidin-AF647 labels biotinylated proteins, while anti-V5 staining shows enzyme expression. FXR1 marks the SGs. Scale bars, 5 μm. (c) Design of DIA-based proteomics for mapping SG proteins using BRET-ID. (d) Volcano plots showing the enrichment of labeled proteins in G3BP1-based BRET-ID labeling under arsenite treatment. Proteins that are significantly enriched in the labeled samples (*p* < 0.05, ratio > 2) are highlighted, with known RNA-binding proteins (RBPs) in pink, known SG proteins in green, and SG orphans in red. (e) GO cellular component analysis of G3BP1-BRET-ID enriched proteins. (f) Volcano plots showing the enrichment of labeled proteins in G3BP1-based BRET-ID labeling under basal conditions. Proteins that are significantly enriched in the labeled samples (*p* < 0.05, ratio > 2) are highlighted, with known RBPs in pink and known SG proteins in green. (g) Percentages of known RBPs in BRET-ID-identified SG proteins under both basal and arsenite conditions. The list of known RBPs was manually collected from previous studies and μMAP-identified SG proteins were provided by a previous study. (h) Confocal fluorescence imaging of RICTOR and TLK1 under basal and arsenite conditions. G3BP1 marks the SGs. Scale bars, 5 μm. (i) G3BP1-GFP knock-in HEK293T cells were pretreated with 10 μM of the selective RICTOR inhibitor JR-AB2-011 for the indicated durations, followed by 500 μM of arsenite treatment for 30 min. The relative ratios of G3BP1 in the SG versus in the cytosol were calculated based on five different areas per well. The error bars show mean ± SD. **, *p* < 0.01; ***, *p* < 0.001 (Student’s *t* test). Scale bars, 30 μm.

We first compared the BRET-ID-labeled sample under arsenite treatment with the nonlabeled control, which omitted the furimazine treatment. We found that several functional SG proteins, including G3BP1, CAPRIN1, PRRC2C, and UBAP2, were among the most significantly enriched proteins (Figure 5d). ROC analysis also indicated that known SG proteins were significantly more enriched than false positives, such as nuclear proteins (Figure S6d). Applying a filter with criteria of fold change > 2 and *p* < 0.05, we retained a list of 674 significantly enriched proteins (Table S4). GO analysis revealed that SG assembly was the most significantly enriched GO term for these proteins (Figure 5e). We also performed untargeted BRET-ID labeling under arsenite stress as a spatial reference. However, this step did not improve the specificity of our data set and actually decreased its sensitivity, consistent with our previous observations on ERM mapping (Figure S6e). Consequently, we omitted this step during the compilation of the final SG protein lists. Nonetheless, using spatial references is crucial for enhancing SG specificity in other studies, such as those employing APEX2 labeling or BL-triggered photocatalytic labeling (Figure S6f)^24,34,35^ .

G3BP1 forms submicroscopic assemblies by preassociating with its binding partners under normal conditions, which act as “seeds” for SG assembly upon stress induction.^24,35^ To map these “preseed” interactors, we also performed G3BP1-BRET-ID labeling under normal conditions and identified 365 significantly enriched proteins (Figure 5f and Table S4). These “preseed” interactors predominantly represent a subset of G3BP1-proximal proteins identified under the arsenite condition (Figure S6g) and exhibit significant enrichment of RBPs and known SG components (Figure S6h). Among these interactors are several SG core proteins, such as CAPRIN1, PRRC2C, UBAP2 and UBAP2L.

BRET-ID-labeled proteins under both basal and stress conditions were enriched in known SG proteins and established RBPs, to a comparable extent as SG proteins identified by μMAP,^24^ further validating the high specificity of our SG data sets (Figures 5f and S6h). We selected two “SG orphans”—proteins not previously identified as SG proteins: TLK1 (serine/threonine-protein kinase tousled-like 1) and RICTOR (rapamycin-insensitive companion of mTOR)—for further localization validation. Immunofluorescence analysis of the two proteins revealed the formation of puncta-like structures, which colocalized with G3BP1 under arsenite-induced stress (Figure 5h). Given that RICTOR is an essential component of mTORC2, a complex previously implicated in heat-induced SG assembly in *Drosophila*,^58^ we investigated whether RICTOR regulates SG formation in arsenite-stressed mammalian cells. HEK293T cells were pretreated with 10 μM of JR-AB2-011 (a selective RICTOR inhibitor)^59^ and subsequently exposed to arsenite. Confocal imaging demonstrated that pharmacological inhibition of RICTOR markedly impaired SG assembly (Figure 5i), supporting its functional role in this process. Collectively, these findings suggest that BRET-ID provides a high-resolution map of the SG proteome, revealing novel SG-associated players.

### G3BP1 Interactome Mapping *In Vivo* by BRET-ID

The nontoxic, rapid PL facilitated by BRET-ID prompted us to explore its potential for *in vivo* applications—an area largely inaccessible to peroxidase-based and blue light-activated PL methods. G3BP1 is overexpressed in various tumor tissues and plays a role in promoting cancer cell proliferation, invasion, and metastasis.^60,61^ It has also been recognized as a potential drug target for cancer treatment and a biomarker for cancer diagnosis.^62^ Mechanistically, G3BP1-centric SGs assist cancer cells in adapting to harsh conditions like hypoxia, nutrient deprivation, and exposure to chemotherapy or radiation, thereby fostering a more aggressive and treatment-resistant tumor phenotype.^63^ Although SG proteomes have been extensively characterized in cultured cells, their compositions are highly context-dependent and disease-specific.^34^ Due to the limitations of current PL tools, the *in vivo* heterogeneity of G3BP1-interacting proteins remains unexplored.

To map the G3BP1 interactomes *in vivo* using BRET-ID, we established tumor xenografts expressing G3BP1-BRET-ID in nude mice (Figure 6a). For labeling, mice were intratumorally injected with furimazine and alkyne-aniline. NanoLuc-generated bioluminescence was then directly observed via live-animal imaging in a furimazine-dependent manner (Figure 6b). Cross sections of furimazine-treated tumors revealed an intrinsic pale yellow signal from furimazine, demonstrating its robust penetration into tumor tissue (Figure S7a). The tumor slices were further click-labeled with azide-biotin, followed by immunofluorescence imaging with streptavidin-AF647, which revealed furimazine-dependent biotinylation across tumor tissues (Figure S7b). Tumor tissues were subsequently harvested and subjected to click labeling with azide-biotin, followed by streptavidin blotting. Promiscuous biotinylation was detected in labeled tumors, while those treated with alkyne-aniline alone showed significantly lower biotinylation (Figure 6c). Since only a limited number of proteins were labeled in the tumor tissues, we employed the fully integrated spintip-based affinity purification-MS technology (FISAP),^64^ which uses a custom C18 tip loaded with streptavidin beads to minimize protein loss and ensure broad coverage of labeled proteins (Figure 6d). Biotinylated proteins bound to the streptavidin beads on the tip, while unmodified proteins were removed by centrifugation. On-bead trypsin digestion was then performed, and the resulting peptides were released into the C18 phase. The peptides were desalted directly on the tip before being eluted for DIA-based LC-MS/MS analysis.

**Figure 6:**
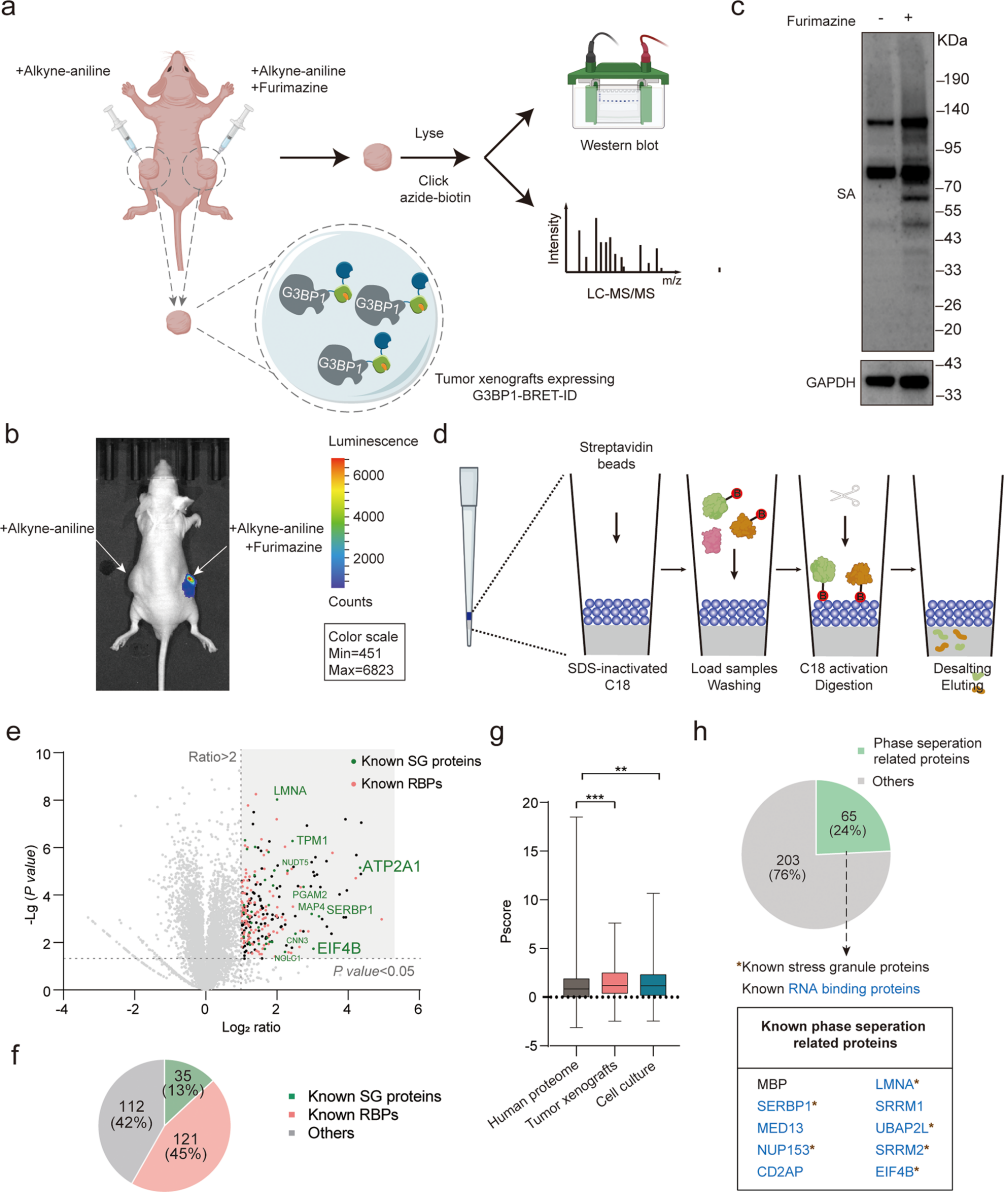
*In vivo* mapping of G3BP1-interacting proteins by BRET-ID. (a) Schematic of *in vivo* BRET-ID labeling to map G3BP1-interacting proteins. Tumor xenografts expressing G3BP1-BRET-ID were intratumorally injected with furimazine and alkyne-aniline, while tumors injected with only alkyne-aniline served as negative controls. Tumor tissues were dissected, lysed, and subjected to a click reaction with azide-biotin, followed by Western blot and LC-MS/MS analysis. (b) *In vivo* imaging of bioluminescence emitted by BRET-ID after the addition of furimazine. (c) Streptavidin blotting of furimazine-dependent G3BP1-BRET-ID labeling in tumor xenografts. Labeled tumor tissues were lysed, reacted with azide-biotin through a click reaction, and then subjected to streptavidin blotting. (d) Schematic of the FISAP platform. A custom C18 tip, preloaded with streptavidin beads, was initially treated with sodium dodecyl sulfate (SDS) to deactivate the C18. BRET-ID-labeled proteins were click-reacted with azide-biotin, then incubated with streptavidin beads for enrichment. Unlabeled proteins were removed by centrifugation, the C18 was reactivated, and on-bead trypsin digestion was performed. The released peptides were desalted directly using C18, followed by elution for DIA-based LC-MS/MS analysis. (e) Volcano plots showing the enrichment of labeled proteins in G3BP1-based BRET-ID labeling in tumor xenografts. Proteins that are significantly enriched in the labeled samples (*p* < 0.05, ratio > 2) are highlighted, with known RBPs in pink and known SG proteins in green. (f) Percentages of known SG proteins and RBPs in BRET-ID-identified G3BP1-interacting proteins from tumor xenografts. (g) Phase separation propensity (Pscore) of G3BP1-interacting proteins identified by BRET-ID in tumor xenografts and cell culture. **, *p* < 0.01; ***, *p* < 0.001 (Wilcoxon rank sum test). (h) Percentages of G3BP1-interacting proteins that are capable of forming biomolecular condensates. Representative proteins with biochemical evidence supporting their ability to undergo liquid–liquid phase separation are shown.

We identified a total of 268 significantly enriched G3BP1-interacting proteins in tumor xenografts (Figure 6e and Table S5). As expected, we observed the enrichment of several well-known G3BP1-interacting proteins and RBPs, such as EIF4B (eukaryotic translation initiation factor 4B), SERBP1 (SERPINE1 mRNA-binding protein 1) and MED13 (Mediator of RNA polymerase II transcription subunit 13) (Figure 6f). Consistent with previous SG data sets, these proteins also exhibited a higher propensity for phase separation, as indicated by increased Pscores^65^ (Figure 6g). Notably, we found that 65 of these proteins had previously been annotated as capable of forming biomolecular condensates in living cells, including EIF4B, SERBP1, and MED13, with biochemical evidence supporting their ability to undergo liquid–liquid phase separation^66–68^ (Figure 6h). The G3BP1-proximal proteins identified in tumor xenografts were notably distinct from those identified in cultured cells (Figure S7c), further emphasizing the heterogeneity of G3BP1 interactomes across different contexts and highlighting the importance of *in vivo* mapping. GO analysis of these proteins in tumor xenografts revealed an enrichment of biological processes more relevant to the *in vivo* environment, such as muscle contraction, blood coagulation, and regulation of T cell proliferation (Figure S7d). In summary, our experiments demonstrate the utility of BRET-ID for *in vivo* PL, with the added advantage of bioluminescence enabling live-animal imaging—a feature not achievable with other PL methods.

## Discussion

PL has become a powerful tool for capturing subcellular protein information and molecular interactions in mass spectrometry experiments, particularly with the recent advancements in genetically encoded photocatalysts that enable the visualization of nanoscale protein arrangements with high spatiotemporal resolution. These techniques include fully genetically encoded photosensitizers, such as miniSOG^11–14^ and SOPP3,^21^ which utilize endogenous chromophores for photocatalysis, as well as HaloTag, which is chemically conjugated to an exogenous photocatalyst.^24–26^ However, in both cases, light irradiation can lead to significant background labeling due to free photocatalysts^29^ and may interfere with cellular activities due to phototoxicity.^69^ Furthermore, these methods cannot be applied in living animals because of the limited penetration of visible light.

The BRET-ID technology we present here employs a fusion or chimeric protein consisting of a bright luciferase and a genetically encoded photosensitizer. Unlike traditional methods that use exogenous blue light, BRET-ID leverages luciferase-generated local blue light to activate the proximal photocatalysts, rather than the free, diffused photocatalysts, via the BRET mechanism. BRET-ID is fully genetically encodable, easy-to-use and nontoxic. We demonstrated that BRET-ID provides highly precise labeling to map subcellular proteomes of open compartments, such as the ER membrane and SGs, and to profile dynamic protein interactomes of GPCRs and kinases. The high spatial specificity of BRET-ID likely stems from its minimalist nonspecific background and the restricted reactive species it generates. Notably, BRET-ID does not require spatial references—typically used in PL studies to enhance specificity at the cost of sensitivity. Moreover, BRET-ID enables significant labeling with just 1 min of furimazine addition, allowing for rapid, 1 min proteomic snapshots of dynamic PPIs. This extraordinary temporal resolution rivals that of the state-of-the-art APEX2 labeling, which also achieves labeling with only 1 min of toxic H_2_O_2_ treatment. Taking advantage of BRET-ID’s high spatiotemporal resolution, we uncovered novel components of SGs such as RICTOR and new interacting proteins with hMOR such as Septin7 and ZYG11B, which were further validated through orthogonal imaging-based approaches.The primary advantage of BRET-ID over traditional visible light-activated genetically encoded photocatalysts is its superior compatibility with *in vivo* settings. Moreover, other existing PL methods also have significant limitations for *in vivo* labeling. For example, APEX2 labeling requires H_2_O_2_, which cannot be administered to live mice, while TurboID suffers from high background due to endogenous biotin levels. While we and others have developed tyrosinase-based PL for *in vivo* studies, it is also limited in extracellular mapping.^70,71^ Recent studies have addressed APEX2’s reliance on cytotoxic H_2_O_2_ by engineering fusion constructs that locally generate H_2_O_2_ to trigger spatially restricted APEX-based biotin-phenol labeling. For instance, SOPP3 produces singlet oxygen species under blue light, which are enzymatically converted to H_2_O_2_ via endogenous superoxide dismutase (SOD).^72^ Separately, the improved APEX (iAPEX) system employs a D-amino acid oxidase to synthesize H_2_O_2_ *in situ*.^73^ These strategies differ fundamentally from BRET-ID, where bioluminescence activates SOPP3 to generate singlet oxygen, oxidizing proximal proteins for subsequent tagging with aniline-based probes. Here, we demonstrate the utility of BRET-ID for *in vivo* labeling in xenograft tumors, using it to identify G3BP1-interacting proteins during tumor progression. This represents the first *in vivo* map of SG proteomes and uncovers distinct G3BP1 partners compared to those identified in cultured cells. Additionally, NanoLuc-emitted bioluminescence enables live-animal imaging, a unique feature not achievable by TurboID or tyrosinase-based methods.^70,71^ Given the broad *in vivo* applications of luciferase and its substrates, BRET-ID is versatile and can be applied to various tissues and organs. As a fully genetically encoded system, BRET-ID can be used to generate transgenic mice with tissue-specific expression, enabling comprehensive *in vivo* proteomic mapping.

BRET-ID does have some limitations. Since the luminescence generated by NanoLuc is much weaker than that produced by exogenous light irradiation, the labeling efficiency of BRET-ID may be relatively low. Although the low levels of singlet oxygen species ensure a short labeling radius, this could also result in reduced sensitivity. This inherent trade-off must be carefully weighed in applications requiring high labeling efficiency, particularly when mapping interactomes of low-abundance proteins. Further optimization and engineering of the BRET-ID fusion protein will be necessary to improve BRET efficiency and labeling sensitivity. Additionally, only intramolecular BRET was performed in this study, although the BRET system has been widely used for detecting protein–protein interactions.^40^ We envision that by fusing NanoLuc with one protein and the genetically encoded photosensitizer with its interacting partner, we can achieve complex-dependent interactome mapping when the two proteins come into close proximity to trigger the BRET. In this regard, we believe BRET-ID bridges the gap between BRET-based detection methods and MS-based proteomics, allowing not only visualization but also the discovery of novel regulators of molecular interactions. We anticipate that BRET-ID will have broad applications in addressing various biological questions in living systems.

## Supplementary Material















## Notes

Table S1 (XLSX)
Table S2 (XLSX)
Table S3 (XLSX)
Table S4 (XLSX)
Table S5 (XLSX)
Additional experimental methods, materials (including protein sequences), supplementary schematic illustrations, condition optimization data, extended mass spectrometry analyses, and other biological evidence supporting the main findings (PDF)
Transparent Peer Review report available (PDF)
