# Supplementary material for: Precise and In Vivo-Compatible Spatial Proteomics via Bioluminescence-Triggered Photocatalytic Proximity Labeling
Source: ACS Cent Sci. 2025 Jul 30;11(9):1611–26. doi: 10.1021/acscentsci.5c00520 (PMC12464768; doi:10.1021/acscentsci.5c00520)
Supplement: Supplementary file 6 [file oc5c00520_si_006.pdf]

Supplementary information for

Precise and *in vivo*-compatible spatial proteomics via bioluminescence-triggered photocatalytic proximity labeling

Xuege Sun[2,3#], Yanling Zhang[2 #], Wenjie Lu[2#], Hongyang Guo[2,3#], Guodong He[2,3], Siyuan Luo[2,3], Haodong Guo[2,3], Zijuan Zhang[2,3], Wenjing Wang[2,4,5,6], Ling Chu[2,4,5], Xiangyu Liu[1,2,3,5], Wei Qin[1,2,3,4,5 *]

[1]The State Key Laboratory of Membrane Biology, Tsinghua University, Beijing, 100084, China. [2]School of Pharmaceutical Sciences, Tsinghua University, Beijing, 100084, China. [3]Tsinghua-Peking Center for Life Sciences, Tsinghua University, Beijing, 100084, China. [4]MOE Key Laboratory of Bioorganic Phosphorus Chemistry & Chemical Biology, Tsinghua University, Beijing, 100084, China. [5]Beijing Frontier Research Center for Biological Structure, Tsinghua University, Beijing, 100084, China. [6]Institute of Medicinal Plant Development, Chinese Academy of Medical Sciences & Peking Union Medical College, Beijing, 100193, China.

*Correspondence: weiqin@tsinghua.edu.cn

[#]These authors contribute equally.

Methods

No unexpected or unusually high safety hazards were encountered.

Cell culture. HEK293T from the ATCC (passages <25) were cultured in DMEM (catalog no. C11995500BT, Gibco) supplemented with 10% fetal bovine serum (catalog no. 04-001-1ACS, Biological Industries), 100 units/mL penicillin and 100 mg/mL streptomycin at 37 °C under 5% CO_2. For fluorescence microscopy imaging experiments, cells were grown on 15-mm glass-bottom cell culture dish (NEST). For proteomic experiments, cells were grown on 10-cm glass-bottomed Petri dishes (NEST). For In-gel fluorescence experiments, cells were grown on six-well plates (NEST).

Construction of stable cell lines. Segments of G3BP1-BRET-ID were inserted into PXL304 vector. The constructed PXL304 plasmids were co-transfected with pMD2.G and pSPAX2 into HEK293T using neofect Transfection Reagent (catalog no. TF201201, Neofect). Viral supernatant was collected 48 hours after transfection and transduced to the indicated cell line for 48 hours. Cells were selected using the complete culture medium with 5 μg/mL Blasticidin S (catalog no. HY-103401, MCE) for approximately 1 week, subsequently maintained in the complete culture medium.

Plasmids. Polymerase chain reactions (PCR) were performed with Phanta DNA Polymerase (catalog no. P520, Vazyme). Ligase-free cloning reactions were performed with Basic Seamless Cloning and Assembly Kit (catalog no. CU201, TransGen).

The DNA of NanoLuc and HaloTag were gifts from Ling Chu at Tsinghua university. The DNA of G3BP1 was a gift from the Pilong Li lab at Tsinghua university. The DNA of miniSOG was a gift from the Peng Zou lab at Peking university. The plasmids contain the DNA sequence of ARRB2, SEPTIN7, ZYG11B were ordered from MIAOLING Biology (catalog no. P64731, P57687, P40492, MIAOLING Biology). The fusion constructs are listed in table S6.

Plasmid products were transformed into DH5α competent cell (catalog no. TSC-C14, Tsingke). DNA sequences were confirmed by Sanger sequencing before use.

Synthesis of chloroalkane-modified HMME.

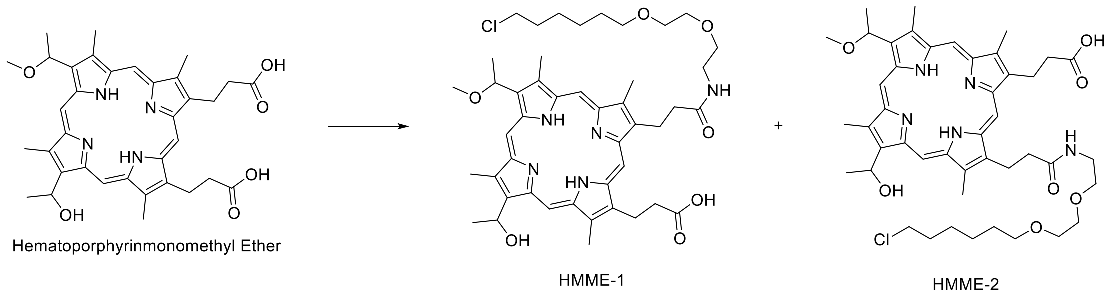

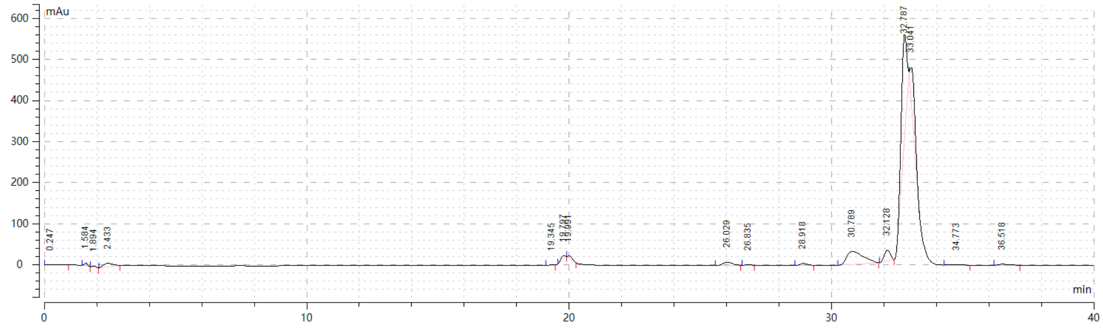

Hematoporphyrinmonomethyl Ether (20 mg, 1.0 eq), TSTU (7.3 mg, 1.0 eq) was dissolved in DMSF (5mL) and stirred for 15 minutes before Ethanamine 2-[2-[(6-chlorohexyl)oxy]ethoxy]-hydrochloride (7.6 mg, 1.2 eq) was added. The solution was stirred at room temperature for 2h and purified by reverse phase HPLC using eluent A (H_2O with 0.1% TFA) and eluent B (CH_3CN) to give the mixture of **HMME-1**and **HMME-2** as light yellow solid. HRMS [C45H61ClN5O7+] Cal: 817.4181, Obs:817.4237.

Chloroalkane-modified HMME (CA-HMME) labeling in living cells. For the labeling of HMME-chloroalkane with cpNluc-HT in living cells, HEK293T cells were transiently transfected with cpNluc-HT for 24 hours. Cells were washed twice with PBS (Solarbio), and then incubated with 5 μM CA-HMME or HMME in DMEM for 60 minutes. Cells were washed 3 times with PBS for 1 minute each time. The cells were then incubated with 1mM alkyne-aniline probe in Hank's Balanced Salt Solution (HBSS, catalog no. H1025, Solarbio) for 15 minutes at 37 °C, followed by 5 minutes blue light treatment or the addition of 75 μM Furimazine (catalog no. T15359, TargetMol) for 45 minutes at 37 °C. The reaction was stopped

by replacing the HBSS and washed 3 times with PBS, harvested by scraping, pelleted by centrifugation at 2,000 r.p.m. for 3 minutes, and either processed immediately or flash frozen in liquid nitrogen and stored at -80 °C before further analysis. The entire experimental procedure was conducted under strict light protection. Cells were collected in amber tubes and kept protected from light until analysis.

BRET-ID labeling in living cells. For the labeling of BRET-ID and other related versions in living cells, HEK293T cells were transiently transfected with BRET-ID fusion construct of interest for 24 hours. Cells were washed twice with PBS (Solarbio), and then incubated with 1mM alkyne-aniline probe in HBSS for 15 minutes at 37 °C, followed by the addition of 75 μM Furimazine for varying durations at room temperature. To evaluate the effect of flavin mononucleotide (FMN) on BRET-ID labeling, 150 μM riboflavin (catalog no. 60340ES60, YEASEN) was co-administered with furimazine during the labeling step. The reaction was stopped by replacing the HBSS and washed three times with PBS, harvested by scraping, pelleted by centrifugation at 2,000 r.p.m. for 3 minutes, and either processed immediately or flash frozen in liquid nitrogen and stored at -80 °C before further analysis. The entire experimental procedure was conducted under strict light protection with red light illumination.

For the labeling of G3BP1 proximal proteins by BRET-ID, HEK293T cells stably expressing G3BP1-BRET-ID were washed twice with PBS (Solarbio), and then incubated with 1mM alkyne-aniline probe in HBSS for 15 minutes at 37 °C, followed by the addition of 75 μM Furimazine for 5 minutes at room temperature. The reaction was stopped by replacing the HBSS and washed three times with PBS, harvested by scraping, pelleted by centrifugation at 2,000 r.p.m. for 3 minutes, and either processed immediately or flash frozen in liquid nitrogen and stored at -80 °C before further analysis. The entire experimental procedure was conducted under strict light protection with red light illumination.

For all cell labeling experiments described above, the cells were lysed by resuspending in RIPA lysis buffer (50 mM Tris pH 8, 150 mM NaCl, 0.1% SDS, 0.5% sodium deoxycholate, 1% Triton X-100, 1× protease inhibitor cocktail (Sigma-Aldrich)) by gentle pipetting. Notably, the RIPA buffer should be EDTA-free because EDTA interferes with the following copper-catalyzed click

reaction. Lysates were clarified by centrifugation at 12,000 *g* for 15 minutes at 4 °C. Protein concentration in clarified lysates was estimated with Pierce BCA Protein Assay Kit (Thermo Fisher) and normalized to 1.5-2 mg/mL. 50 µL of lysates were reacted with 100 µM azide-Rhodamine or azide-biotin, premixed 2-(4-((bis((1-tertbutyl-1H-1,2,3-triazol-4-yl)methyl)amino)methyl)-1H-1,2,3-triazol1-yl)-acetic acid (BTTAA)-$CuSO_4$ complex (500 µM $CuSO_4$, BTTAA:$CuSO_4$ with a 2:1 molar ratio) and 0.5 mg/mL freshly prepared sodium ascorbate for 2 hours at room temperature. The excess reagents were removed by methanol and chloroform (4:1) precipitation and the fluorescent intensity was detected by in-gel fluorescence scanning or western blot.

For the labeling of µ-Opioid receptor proximal proteins by BRET-ID, HEK293T cells were transiently transfected with Flag-hMOR-BRET-ID for 36 hours. Cells were washed twice with PBS (Solarbio), and then incubated with 500 µM biotin aniline probe in HBSS for 30 minutes. Then, 10 µM DAMGO ([d-Ala2, *N*-Me-Phe4, Gly5-ol]-enkephalin acetate salt) (catalog no. HY-P0210, MCE) was added for the noted period of time (0 minutes, 5 minutes or 30 minutes). BRET-ID labeling was initiated by the addition of 75 µM Furimazine at room temperature. The labeling reaction was allowed to continue for 1 minute, media were removed, and the cells were washed three times with PBS. The cells were harvested by scraping, pelleted by centrifugation at 2,000 r.p.m. for 3 minutes, and either processed immediately or flash frozen in liquid nitrogen and stored at -80 °C before further analysis.

Quantification of fluorescence intensity. Cells expressing BRET-ID or NanoLuc were seeded at a density of 5000 cells per well in a 96-well plate and incubated overnight. Cells were lysed with 50 µL of passive lysis buffer (catalog no. E1941, Promega) containing 7.5 µM Furimazine and 20 mM riboflavin, and fluorescence intensities at different wavelengths were immediately measured using a plate reader (Spark multimode microplate reader, TECAN). Signal intensities were normalized to the highest peak, which was set to 1.

In-gel fluorescence scanning and Western blotting. For all in-gel fluorescence experiments, samples were separated on 10% SDS-PAGE gels and imaged using a Tanon 5200Multi imaging system. Gels were subsequently stained with Coomassie Brilliant Blue to verify equal protein

loading. For all Western blotting experiments, samples were resolved on 10% SDS-PAGE gels and transferred to PVDF membranes. Membranes were briefly stained with Ponceau S solution (0.1% w/v Ponceau S in 5% acetic acid) for 5 minutes to assess transfer efficiency, then blocked in 5% (w/v) skim milk in TBS-T (Tris-buffered saline containing 0.1% Tween-20) for at least 30 minutes at room temperature. For streptavidin blotting, membranes were incubated with HRP-conjugated streptavidin (1:2,000 dilution; Beyotime, A0303) for 1 hour at room temperature. For V5 blotting, membranes were incubated with anti-V5 antibody (1:2,000 dilution; ABclonal, AE071) for 2 hours at room temperature or overnight at 4 °C. For HA blotting, the blots were stained with anti-HA (1:2,000 dilution, catalog no. HT301-01, TransGen Biotech) for 2 hours at room temperature or 4 ℃ overnight. For G3BP1 blotting, the blots were stained with anti-G3BP1 (1:2,000 dilution, catalog no. A3968, ABclonal) for 2 hours at room temperature or 4 ℃ overnight. After washing three times with TBS-T for 5 minutes each, the blots were stained with secondary antibodies in TBS-T for 1 hour at room temperature. The blots were washed three times with TBS-T for 5 minutes each time before to development with Clarity Western ECL Blotting Substrates (catalog no. E1070, LABLEAD) and imaging on the Tanon 5200multi.

Confocal fluorescence microscopy. To validate BRET-ID labeling across different subcellular localizations, HEK293T cells were transiently transfected with the BRET-ID fusion construct of interest or the empty vector control for 24 hours. Cells were washed twice with PBS (Solarbio), and then incubated with 1mM alkyne-aniline probe in HBSS for 15 minutes at 37 °C, followed by the addition of 75 μM Furimazine for 1 minute at room temperature. Cells were washed with PBS for 3 times and fixed for 15 minutes with pre-cooled carbinol at -20 ℃. After 3 times washes in PBS, alkyne labeled proteins were then reacted with 100 μM azide-biotin, premixed 2-(4-((bis((1-tertbutyl-1H-1,2,3-triazol-4-yl)methyl)amino)methyl)-1H-1,2,3-triazol1-yl)-acetic acid (BTTAA)-$CuSO_4$ complex (500 μM $CuSO_4$, BTTAA:$CuSO_4$ with a 2:1 molar ratio) and 0.5 mg/mL freshly prepared sodium ascorbate in PBS for 1 hour at 25 °C. Cells were washed 3 times in PBS and blocked in 5% BSA dissolved in PBS for at least 15 minutes. Cell dishes were incubated with primary antibodies against v5(1:250 dilution, catalog no. 460705, Thermo Fisher) and Citrate Synthase (1:250 dilution, catalog no. BM5202, BOSTER) or Calnexin (1:250

dilution, catalog no. A15631, ABclonal) overnight at 4 °C. Cells were washed three times in PBS with 5 minutes for each wash then incubated in secondary antibodies conjugated to either AlexaFluor-488 or 568 and streptavidin conjugated to AlexaFluor647 for 1 hour. Cells were washed 3 times in PBST (0.1% Tween-20 in PBS) and incubated with DAPI (1:2000 dilution, catalog no. C1002, Beyotime) for 10 minutes.

To validate the labeling of G3BP1-BRET-ID, HEK293T cells stably expressing G3BP1-BRET-ID were treated with 500 μM sodium arsenite for an hour or not. Then washed twice with PBS (Solarbio) and incubated with 1mM alkyne-aniline probe in HBSS for 15 minutes at 37 °C, followed by the addition of 75 μM Furimazine for 1 minute at room temperature. Cells were washed with PBS for 3 times and fixed for 15 minutes with pre-cooled carbinol at -20 °C. After 3 times washes in PBS, alkyne labeled proteins were then reacted with 100 μM azide-biotin, premixed 2-(4-((bis((1-tertbutyl-1H-1,2,3-triazol-4-yl)methyl)amino)methyl)-1H-1,2,3-triazol1-yl)-acetic acid (BTTAA)-$CuSO_4$ complex (500 μM $CuSO_4$, BTTAA:$CuSO_4$ with a 2:1 molar ratio) and 0.5 mg/mL freshly prepared sodium ascorbate in PBS for 1 hour at 25 °C. Cells were washed 3 times in PBS and blocked in 5% BSA dissolved in PBS for at least 15 minutes. Cells were incubated with primary antibodies against v5(1:250 dilution, catalog no. 460705, Thermo Fisher) and FXR1(1:250 dilution, catalog no. sc-374148, Santa Cruz Biotechnology) overnight at 4 °C and then washed three times in PBS with 5 minutes for each wash then incubated in secondary antibodies conjugated to either AlexaFluor-488 or 568 and streptavidin conjugated to AlexaFluor647 for 1 hour. Cells were washed 3 times in PBST (0.1% Tween-20 in PBS) and incubated with DAPI (1:2000 dilution, catalog no. C1002, Beyotime) for 10 minutes.

To validate the G3BP1 interacting proteins, HEK293T cells were treated with 500 μM sodium arsenite for an hour or not and washed with PBS for 3 times before fixed for 15 minutes with pre-cooled carbinol at -20 °C. After 3 times washes in PBS, cells were incubated with primary antibodies against G3BP1(1:250 dilution, catalog no. A3968, ABclonal) and TLK1(1:50 dilution, catalog no. sc-393515, Proteintech) or RICTOR (1:250 dilution, catalog no. 66867-2-Ig, Proteintech) overnight at 4 °C. Cells were washed three times in PBS with 5 minutes for each wash then incubated in secondary antibodies conjugated to either AlexaFluor-488 and 568 for 1

hour. Cells were washed 3 times in PBST (0.1% Tween-20 in PBS) and them incubated with DAPI (1:2000 dilution, catalog no. C1002, Beyotime) for 10 minutes.

To validate the effect of the RICTOR inhibitor on SG formation, HEK293T cells with GFP knocked into the G3BP1 locus were seeded into imaging dishes. When the cells reached approximately 60–80% confluency, they were treated with 10 μM JR-AB2-011 for 0.5, 1.5, or 2.5 hours. Subsequently, 500 μM sodium arsenite was added for 30 minutes to induce SG formation, followed by live-cell imaging. Samples were incubated in an environmental control chamber at 37 °C and 5% CO_2 atmosphere during the entire imaging process.

All confocal imaging was performed using a Nikon AXR-NSPARC (Nikon Spatial Array Confocal) microscope. Image processing and quantification were conducted using ImageJ2/FIJI software. Subcellular localization was analyzed using the "Plot Profile" function to extract fluorescence intensity distributions along defined regions of interest. Stress granules (SGs) and cytosolic regions were segmented based on intensity thresholds, and quantitative measurements were performed on thresholded images acquired from randomly selected fields of view.

Cell viability assays. Cells were transfected with BRET-ID plasmids for 6 hours, then digested and seeded in a 96-well plate 10,000 cells per well (six technical replicates). Cells were then treated with 75 μM furimazine or 1 mM alkyne-aniline, or blue light irradiation for noted time. After 3 times washes with PBS, cells were incubated with medium without FBS for another 24 hours. Cell viability was then detected by using MTS reagent (CellTiter 96® AQueous One Solution Cell Proliferation Assay, catalog no. G3580, Promega). Cells were incubated in 100 μL serum-free medium (DMEM) containing 1/6 of the MTS reagent at 37 °C for 30 minutes and the absorbance at 490 nm was measured with a microplate reader. For data presentation, the mean and standard deviation for the three biological replicates of each data point in a representative experiment were plotted in Prism 9 (Graphpad) and presented. The fitted curves were regressed by "Inhibitor vs Response" in Graphpad Prism software.

GloSensor assay to evaluate the function of human μOR. This assay was performed following a similar methods as previous reported[1]. To evaluate the function of hMOR-BRET, HEK293T

cells were co-transfected at a 1:1 ratio with either human μ-opioid receptor wild type (Flag-hMOR) or human μ-opioid receptor fused with BRET-ID (Flag-hMOR-BRET-ID), along with a split-luciferase based cAMP biosensor (pGloSensorTM-22F, catalog no. E1171, Promega) using PEI as the transfection reagent. Transfected cells were seeded into 96-well plate and incubated at 37 °C, 5% CO_2 overnight. The next day, medium was replaced with CO_2-independent medium containing 150 μg/mL luciferin, and the cells were incubated for 1 hour at 37 °C followed by another 1 hour at room temperature. After incubation, the baseline of luminescence was measure before drug stimulation. To activate hMOR, different concentrations of DAMGO were added to each well and incubated at room temperature for 15 minutes. To stimulate endogenous cAMP via β2-adrenergic-Gs activation, a final concentration of 200 nM isoprenaline was added per well. Then the luminescence of each well was measured every minute using a plate reader (Spark multimode microplate reader, TECAN). Data were analyzed using nonlinear regression in GraphPad Prism (GraphPad Software Inc., San Diego, CA) and normalized to mock control.

Co-Immunoprecipitation. HEK293T cells were cultured in a 6-well plate and allowed to reach ~60-70% confluency before co-transfected with Flag-tagged human μ-opioid receptor wild type (Flag-hMOR) and HA-tagged ARRB2, ZYG11B or Septin7. The cells were lysed in 400μL lysis buffer (20mM HEPES,150mM NaCl,1%(w/v) LMNG and 1%(w/v) CHS) at room temperature for 30 minutes, followed by 20000g-centrifugation at 4°C for 30 minutes. The protein concentration was normalized to a final protein concentration of 2 mg/mL in 400μL using BCA Protein Assay Kit. Anti-Flag beads (catalog no. M8823, Sigma) were rinsed twice in lysis buffer using 30μL of beads for each harvested well. A 20μL aliquot of the cell lysates was collected as input, and the remaining supernatant was added lysis buffer and 5% BSA up to 400μL and incubated with the beads at 4°C with rotation for 3 hours. The beads were washed three times with lysis buffer, and the immunoprecipitants were resuspended in 2×SDS loading buffer. Protein was eluted by heating at 95°C for 10 minutes before analysis of protein content by immunoblotting.

Proximity Ligation Assay. HEK293T cells were seeded into 4-chamber glass-bottom dishes. Flag-tagged hMOR plasmid was co-transfected with HA-tagged candidate interactors, using a

HA-tagged NLS plasmid as a negative control. Twenty-four hours post-transfection, cells were washed once with PBS and fixed in pre-chilled methanol at –20 °C for 20 minutes. Proximity ligation assay was performed using the Duolink® In Situ Orange Starter Kit Mouse/Rabbit (catalog no. DUO92007, Millipore Sigma) according to the manufacturer's instructions.

Animal experiments. For xenograft mouse model, male BALB/c nude mice aged 3-4 weeks were obtained from Beijing Vital River Laboratory Animal Technology Co., Ltd (Beijing, China). The animals were housed in pressurized, individually ventilated cages (PIV/IVC) and maintained under specific-pathogen-free conditions, with free access to food and water in a controlled 12-hour light/dark cycle. All animal experiments were conducted in accordance with the ethical standards and approved by the Institutional Animal Care and Use Committees of Tsinghua University (Beijing, China).

Xenograft Mouse Model. HEK293T cells stably expressing G3BP1-BRET-ID were resuspended in PBS with the density of 5×10^7 cells per mL, and were injected into the flank of hind legs of BALB/c nude mice in a volume of 0.1 mL on both sides through s.c.. 1 week later. When the tumor volume reached approximately 100 mm^3, as measured by caliper, mice were anesthetized and injected intratumorally at multiple points with 0.1 mL of a 20 mM alkyne-aniline solution in PBS on both sides of the tumor. One hour after the initial injection, 0.1 mL of a solution containing 4 mM Furimazine and 20 mM alkyne-aniline in PBS was injected intratumorally into the right tumor, while the left tumor received a second injection of 20 mM alkyne-aniline in PBS (0.1 mL). Luminescence was then monitored using a PerkinElmer IVIS Spectrum bioluminescent imaging system (Waltham, MA, USA). After an additional hour, 0.06 mL of a 50 mM alkyne-aniline solution in PBS was injected intratumorally at multiple points into both tumors. Mice were euthanized, and tumors were harvested 12 hours following the final injection. The maximum tumor size approved by IACUC protocol was 20 mm in diameter and this was not exceeded in all experiments.

Cryosectioning and immunofluorescence staining of tumor tissues. Tumors were harvested from live mice and stored at −80 °C. Prior to sectioning, tissues were fixed in 4% paraformaldehyde (PFA) at 4 °C overnight. Fixed tissues were then dehydrated sequentially in

15% and 30% sucrose (in PBS) for 12 hours each at 4 °C. After dehydration, tissues were briefly blotted dry, embedded in OCT compound (catalog no.4583, Sakura Tissue-Tek), and snap-frozen in liquid nitrogen. Embedded blocks were stored at −80 °C until sectioning. Cryosections were cut at a thickness of 10 μm and mounted onto microscope slides. Slides were immersed in antigen retrieval buffer (catalog no. PR30001, Proteintech) and heated at 95 °C for 1 hour in a water bath, then allowed to cool to room temperature in PBS. Endogenous biotin was blocked using a commercial biotin-blocking kit (catalog no. P0101, Beyotime): Biotin Blocking Buffer was applied for 30 minutes, followed by three 5-minute PBS washes, then Streptavidin/Biotin-Binding Blocking Buffer was applied for another 30 minutes. Slides were washed three times (5 minutes each) in PBST (PBS + 0.05% Tween-20). Sections were then blocked with 5% BSA in PBS for 2 hours at room temperature.

Click chemistry was performed in a total volume of 100 μl, consisting of 85 μl PBS, 4 μl of a premixed BTTAA (25 mM)/$CuSO_4$ (12.5 mM) solution, 6 μl sodium ascorbate (10 mg/ml), and 5 μl N_3-biotin (2 mM; final concentration 100 μM). The mixture was applied to the sections and incubated at room temperature for 2 hours in the dark. Slides were washed three times in PBST. Streptavidin-Alexa Fluor 647 was added and incubated at room temperature for 1 hour, followed by DAPI staining (1:2000 in PBS) for 10 minutes. After a final round of three PBST washes, slides were mounted with antifade mounting medium and sealed with a coverslip and nail polish.

All confocal imaging was performed using a Nikon AXR-NSPARC (Nikon Spatial Array Confocal) microscope.

Streptavidin enrichment. Cells in a 10cm dish were lysed in 1 mL EDTA-free RIPA lysis buffer on ice for 30 minutes. Samples were centrifuged at 12,000g for 15 minutes at 4 °C and the protein concentration was normalized to a final protein concentration of 2 mg/mL using BCA Protein Assay Kit. The Supernatant was removed and subjected to click reaction with N_3-Biotin via CuAAC for 2h at room temperature. After the click reaction, the proteins were extracted using chloroform-methanol precipitation and dissolved in RIPA buffer. 200 μL streptavidin magnetic beads (ChomiX Biotech Co., Ltd. Nanjing, China, cat. no. 02030002) were washed with RIPA once and co-incubated with samples on a rotary shaker overnight at 4 °C.

For the hMOR-BRET-ID labeling samples, the cells were lysed in 1 mL EDTA-free RIPA lysis buffer on ice for 30 minutes. Samples were centrifuged at 20,000g for 30 minutes at 4 °C and the protein concentration was normalized to a final protein concentration of 2 mg/mL using BCA Protein Assay Kit. 200 μL streptavidin magnetic beads (ChomiX Biotech Co., Ltd. Nanjing, China, cat. no. 02030002) were washed with RIPA once and co-incubated with 1 mL lysate on a rotary shaker overnight at 4 °C.

DIA-based quantitative proteomics. Pellet the beads on a magnetic rack and wash the beads twice with 1 mL RIPA buffer, once with 1 mL of 1 M KCl, once with 1 mL of 0.1 M Na_2CO_3, once with 1 mL of 2 M urea in 10 mM Tris-HCl (pH 8.0), and twice with RIPA buffer. The enriched proteins with magnetic beads were washed twice by 1 mL PBS, the resulting beads were resuspended in 500 μL 100 mM triethylammonium bicarbonate (TEAB) buffer with 6 M urea and 10 mM dithiothreitol (DTT) at 35 °C for 30 minutes and alkylated by addition of 20 mM iodoacetamide (IAA) at 37 °C for 30 minutes in the dark. The beads were then washed with 1 mL of 100 mM TEAB buffer and resuspended in 200 μL of 100 mM TEAB buffer and 10 ng/μL trypsin (Meizhiyuan). Trypsin digestion was performed at 37 °C on a thermomixer overnight. Collect the digested peptides in fresh microcentrifuge tubes, wash the the beads twice with 50 μL of 100 mM TEAB buffer and the supernatant was combined with the peptide solution.

The eluted peptides were desalted on C18 StageTips (catalog no. WAT036905, Waters): The desalting column was activated and equilibrated by ACN and 0.1% FA water respectively. The peptide samples were loaded onto the column three times. Then, 1 mL of 0.1% FA in water was added and this washing step was repeated three times. Subsequently, 300 μL of 30%, 50%, and 80% ACN were added, and the eluted peptides were combined.

For the FISAP samples, one plug of the C18 SPE disc was inserted into a standard 200 μL tip, then 15 mg of C18 packing material was dissolved in acetonitrile and added into tip. 60 μL of methanol was added and centrifuged at 3000 *g* for 3 mihutes for conditioning and 60 μL of 2% SDS was added and centrifuged at 3000 *g* for 3 minutes to blocking C18. Streptavidin beads were washed twice with 1 mL RIPA lysis buffer and then resuspend with RIPA buffer. The pre-

washed Streptavidin beads slurry was loaded and centrifuged at 1000 *g* for 3 minutes. 100 µg conditioned medium was then loaded onto the tip and centrifuged at 100 *g* for 1 h. After biotinylated proteins were captured, the tips were washed 4 times with 60 µL of RIPA buffer at 1500 *g* for 1 minutes. Then C18 was activated with 60 µL of 0.5%(v/v) acetic acid in 80% acetonitrile by centrifuged at 6000 *g* for 3 minutes. For reduction of cysteine residues, 20 µL of 50 mM ammonium bicarbonate containing 10 mM DTT was added and incubated at 25 °C with 600 rpm for 15 min, the solution was removed with centrifugation at 6000 *g* for 3 minutes. For digestion, 10 µL 50 mM ammonium bicarbonate containing 0.25 µg/µL trypsin, 50 mM IAA was loaded onto the tips and the samples were incubated at 37 °C avoid light for 1 h with 600 rpm to digest and alkylate the proteins. The digested peptides (retained on C18) were washed with 60 µL 10 mM pH=10 ammonium bicarbonate for three times. Afterwards, the peptides were eluted with 60 µL of 0.5% (v/v) acetic acid in 80% acetonitrile at 5000 *g* for 5 minutes three times. Sample was dried in a vacuum centrifuge and stored at -80 °C until analysis.

LC-MS/MS analysis. Peptides were separated using a loading column (100 µm × 2 cm) and a C18 separating capillary column (100 µm × 15 cm) packed in-house with Luna 3 µm C18(2) bulk packing material (Phenomenex, USA). The mobile phases (A: water with 0.1% formic acid and B: 94% acetonitrile with 0.1% formic acid) were driven and controlled by a Vanquish™ Neo UHPLC system (Thermo Fisher Scientific). The LC gradient for protein samples was held at 4% B for the first 4 minutes of the analysis, followed by an increase from 5% to 20% B from 4 to 109 minutes, an increase from 20% to 35% B from 109 to 150 minutes and an increase from 35% to 99% B from 150 to 159 minutes. For the samples analyzed by Q Exactive-plus series Orbitrap mass spectrometers (Thermo Fisher Scientific), the precursors were ionized using an EASY-Spray ionization source (Thermo Fisher Scientific) source held at +2.0 kV compared to ground, and the inlet capillary temperature was held at 320 °C. Survey scans of peptide precursors were collected in the Orbitrap from 350-1800 Th with an AGC target of 3,000,000, a maximum injection time of 20 ms and a resolution of 70,000. The data-independent acquisition mode was selected. For each DIA window, resolution was set to 17,500. AGC target value for fragment

spectra was set at 1,000,000 with an auto IT. Normalized CE was set at 28%. Default charge was 3 and the fixed first mass was set to 200 Th.

Data analysis. The raw data were processed using DIA-NN in an advanced library-free module. The main search settings for in silico library generation were set as following: trypsin/P with maximum 3 missed cleavage; protein N-terminal M excision on; carbamidomethyl on C as fixed modification; oxidation on M as variable modification; peptide length from 5-30; precursor charge 1-4; precursor m/z from 250 to 1800; fragment m/z from 200 to 1800. The Human UniProt isoform sequence database (3AUP000005640) was used to annotate proteins for human cell samples. Other search parameters were set as following: quantification strategy was set to ‘‘QuantUMS (high precision)’’ mode; cross-run normalization was off; MS2 and MS1 mass accuracies were set to 0, allowing the DIA-NN to automatically determine mass tolerances; Scan window was set to 0 corresponding to the approximate average number of data points per peak; Peptidoforms and MBR were turned on; neural network classifier was single-pass mode.

Receiver operating characteristic (ROC) analysis. For all proteomes identified, we performed ROC analysis to assess their subcellular distribution specificity. For Endoplasmic reticulum membrane proteome compared with unlabeled control, 90 established ERM-localized proteins were defined as ‘true positive’, 173 mitochondrial matrix proteins were defined as were defined as ‘false positive’ (**Table S1**). For Endoplasmic reticulum membrane proteome compared with spatial reference, 90 established ERM-localized proteins were defined as ‘true positive’, 7421 proteins with non-secretory annotation in TurboID work[2] were defined as ‘false positive’ (**Table S2**). For hMOR interactome, the ‘false positives’ (FPs) were gold standard nuclear proteins assembled in TurboID work[2]. the ‘ture positives’(TPs) were plasma membrane proteins annotated by GO:0005886 (**Table S3**). For stress granule proteome, true-positive proteins (TPs) were GOCC-annotated stress granule proteins and false positives (FPs) were nuclear proteins mentioned above (**Table S4**).

Statistical analysis. Gene ontology analysis was conducted using the DAVID databases. All the bar and line charts in the figures were generated using GraphPad Prism 10. Heatmap was plotted by https://www.bioinformatics.com.cn (last accessed on 10 Dec 2024), an online platform for

data analysis and visualization. Some of the schematic workflows were created in https://BioRender.com.

Supplementary Figures

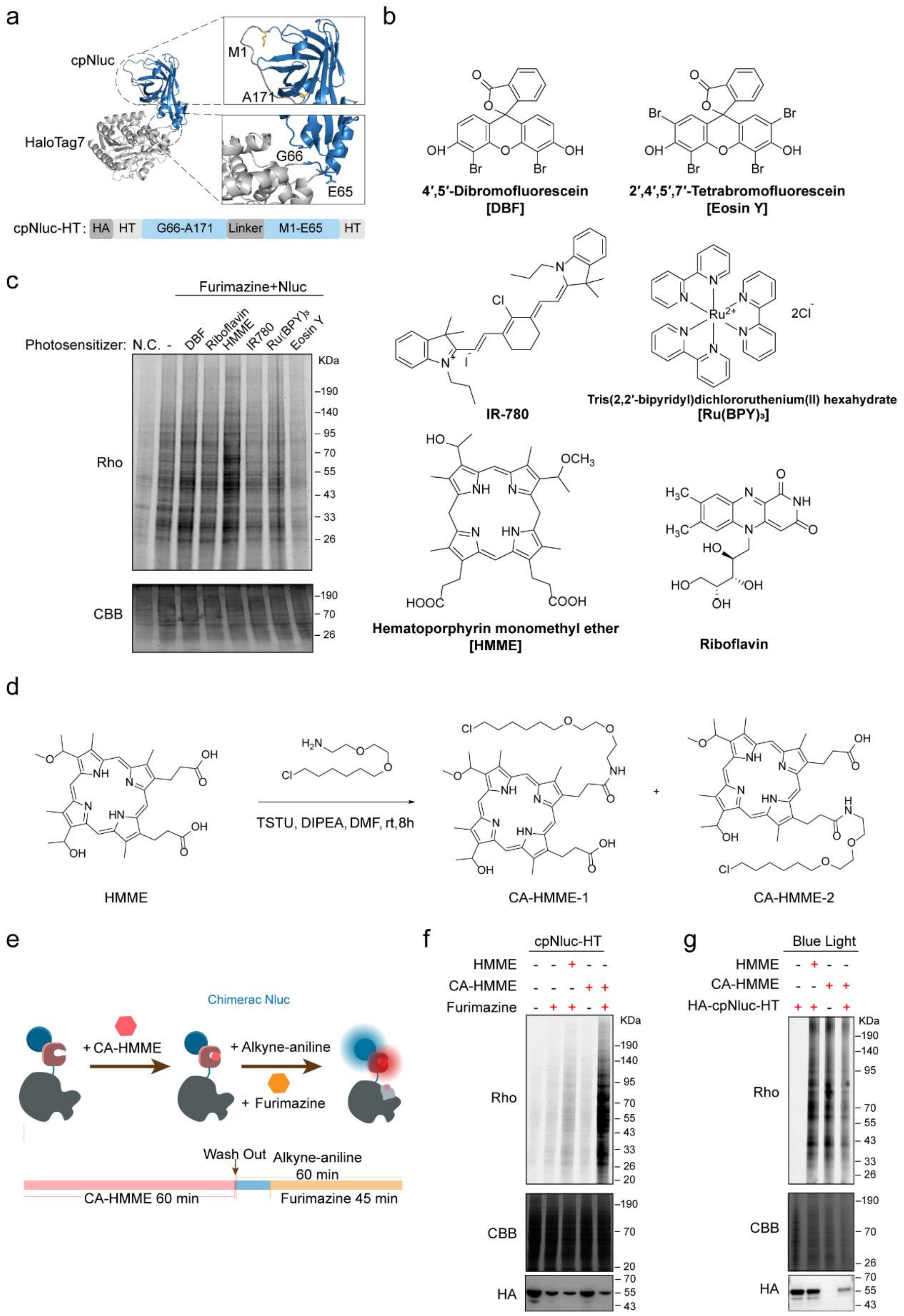

a
cpNluc
M1
A171
HaloTag7
G66
E65
cpNluc-HT: HA HT G66-A171 Linker M1-E65 HT
b
4′,5′-Dibromofluorescein [DBF]
2′,4′,5′,7′-Tetrabromofluorescein [Eosin Y]
IR-780
Tris(2,2′-bipyridyl)dichlororuthenium(II) hexahydrate [Ru(BPY)3]
Hematoporphyrin monomethyl ether [HMME]
Riboflavin
c
Furimazine+Nluc
Photosensitizer: N.C. - DBF Riboflavin HMME IR780 Ru(BPY)3 Eosin Y
KDa
Rho
CBB
d
HMME
TSTU, DIPEA, DMF, rt, 8h
CA-HMME-1
CA-HMME-2
e
Chimerac Nluc
+ CA-HMME
+ Alkyne-aniline
+ Furimazine
Wash Out
Alkyne-aniline 60 min
CA-HMME 60 min
Furimazine 45 min
f
cpNluc-HT
HMME
CA-HMME
Furimazine
Rho
CBB
HA
g
Blue Light
HMME
CA-HMME
HA-cpNluc-HT
Rho
CBB
HA

Figure S1. **Identification of photocatalysts for the development of chemogenetic BRET-ID**. **a.** The AlphaFold-predicted structure of the chimeric cpNluc-HT fusion protein, in which the circularly permuted NanoLuc is inserted into the HaloTag. **b.** The chemical structures of tested photocatalysts, including DBF, eosin Y, IR-780, Ru(BPY)$_3$, HMME and riboflavin. **c.** Evaluation of the NanoLuc-triggered labeling efficiency with various photocatalysts. HEK293T cells were transfected with NanoLuc, treated with 5 μM different photocatalysts and 1 mM alkyne-aniline for 1 hour, followed by co-treatment with 75 μM furimazine and 1 mM alkyne-aniline for an additional 45 minutes. The labeled cells were lysed, and the lysates were subjected to click chemistry with azide-Rhodamine, followed by in-gel fluorescence scanning. Coomassie Brilliant Blue (CBB) staining was used to confirm equal protein loading. **d.** The synthetic route of the chloroalkane-modified HMME (CA-HMME). The final products are isomers (CA-HMME-1 and CA-HMME-2). **e.** Experimental workflow for evaluating the efficiency of chemogenetic BRET-ID. Cells expressing cpNluc-HT were treated with 5 μM haloalkane-modified HMME (CA-HMME) for 1 hour, followed by treatment with 1 mM alkyne-aniline for 15 minutes, and co-treatment with 75 μM of furimazine and 1 mM of alkyne-aniline for an additional 45 minutes. **f.** In-gel fluorescence scanning of cpNluc-HT labeling following furimazine addition, using the workflow described in panel **e**. **g.** In-gel fluorescence scanning of cpNluc-HT labeling upon blue light irradiation. In **f-g**, the labeled cells were lysed, and the lysates were subjected to click chemistry with azide-Rhodamine, followed by in-gel fluorescence scanning. Coomassie Brilliant Blue (CBB) staining was used to confirm equal protein loading and anti-HA blotting indicates enzyme expression.

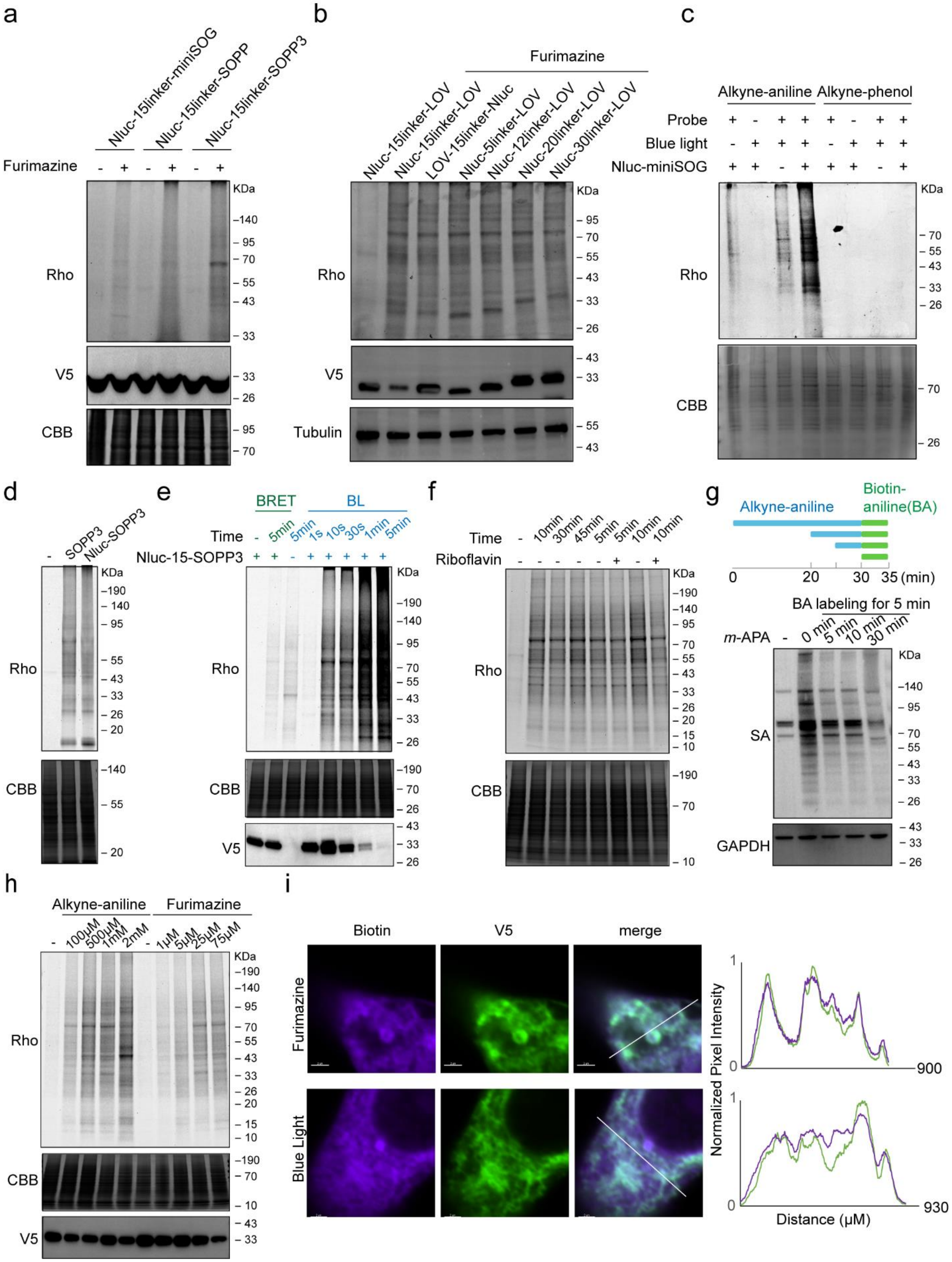
a
Nluc-15linker-miniSOG
Nluc-15linker-SOPP
Nluc-15linker-SOPP3
Furimazine
KDa
Rho
V5
CBB
b
Furimazine
Nluc-15linker-LOV
Nluc-15linker-LOV
LOV-15linker-Nluc
Nluc-5linker-LOV
Nluc-12linker-LOV
Nluc-20linker-LOV
Nluc-30linker-LOV
Tubulin
c
Alkyne-aniline
Alkyne-phenol
Probe
Blue light
Nluc-miniSOG
d
SOPP3
Nluc-SOPP3
e
BRET
BL
Time
Nluc-15-SOPP3
f
Riboflavin
g
Alkyne-aniline
Biotin-aniline(BA)
(min)
BA labeling for 5 min
m-APA
SA
GAPDH
h
Alkyne-aniline
Furimazine
i
Biotin
V5
merge
Furimazine
Blue Light
Normalized Pixel Intensity
Distance (μM)

Figure S2. Optimization of the genetically encoded BRET-ID system. a. Evaluation of different LOV domains for BRET-ID labeling. **b**. Evaluation of different linker strategies for BRET-ID labeling. In **a** and **b**, HEK293T cells were transfected with indicated constructs and treated with 1 mM alkyne-aniline for 15 minutes, followed by the treatment of 75 μM furimazine and 1 mM alkyne-aniline for 45 minutes. **c**. Evaluation of chemical probes for BRET-ID labeling. HEK293T cells were transfected with NanoLuc-miniSOG for 24 hours and treated with 1mM alkyne-aniline or alkyne-phenol for 30 minutes, followed by 130 mW/cm^2 blue light irradiation for 5 minutes. **d**. The impact of NanoLuc on the photocatalytic labeling efficiency of SOPP3. HEK293T cells were transfected with SOPP3 or NanoLuc-15-SOPP3, and treated with 1 mM alkyne-aniline for 15 minutes, followed by 200 mW/cm^2 blue light irradiation for 10 minutes. **e**. Comparison of the labeling efficiency under BRET activation and blue light irradiation at different labeling time. For BRET-triggered labeling, HEK293T cells expressing NanoLuc-15-SOPP3 were treated with 1 mM alkyne-aniline for 15 minutes, followed by the treatment of 75 μM furimazine and 1 mM alkyne-aniline for 5 minutes. For blue light-triggered labeling, HEK293T cells expressing Nanoluc-15-SOPP3 were treated with 1 mM alkyne-aniline for 15 minutes, followed by 200 mW/cm^2 blue light irradiation for various durations. **f**. HEK293T cells expressing the BRET-ID construct were pre-incubated with 1 mM alkyne-aniline for 15 minutes, followed by treatment with 75 μM furimazine for 5 minutes, with or without 150 μM riboflavin supplementation. **g.** The impact of BRET-ID labeling on the enzymatic activity of NanoLuc-15-SOPP3. The schematic of various labeling conditions is shown. HEK293T cells expressing Nanoluc-15-SOPP3 were subjected to alkyne-aniline labeling for various duration, followed by 5 minutes of biotin-aniline (BA) labeling. The biotinylation extent was determined by streptavidin blotting. **h**. The impact of alkyne-aniline and furimazine concentrations on BRET-ID labeling. For the evaluation of alkyne-aniline concentrations, HEK293T cells expressing NanoLuc-15-SOPP3 were treated with various concentrations of alkyne-aniline for 15 minutes, followed by co-treatment with 75 μM furimazine for 5 minutes. For the evaluation of furimazine concentrations, HEK293T cells expressing NanoLuc-15-SOPP3 were treated with 1 mM alkyne-aniline for 15 minutes, followed by co-treatment with various concentrations of

furimazine for 5 minutes. In a-e and g, the labeled cells were lysed, and the lysates were subjected to click chemistry with azide-Rhodamine, followed by in-gel fluorescence scanning. **i**. Confocal fluorescence imaging of BRET-ID labeling on ERM. HEK293T cells were transfected with BRET-ID-ERM for 24 hours, followed by furimazine-based labeling initiation. Cells were fixed and labeled with azide-biotin, followed by staining with Streptavidin-AF647 for visualization of labeled proteins. Anti-V5 staining indicates enzyme expression. White lines indicate where line plots were generated. Average intensity of biotinylation and V5 staining was quantified. Scale bars, 2 μm.

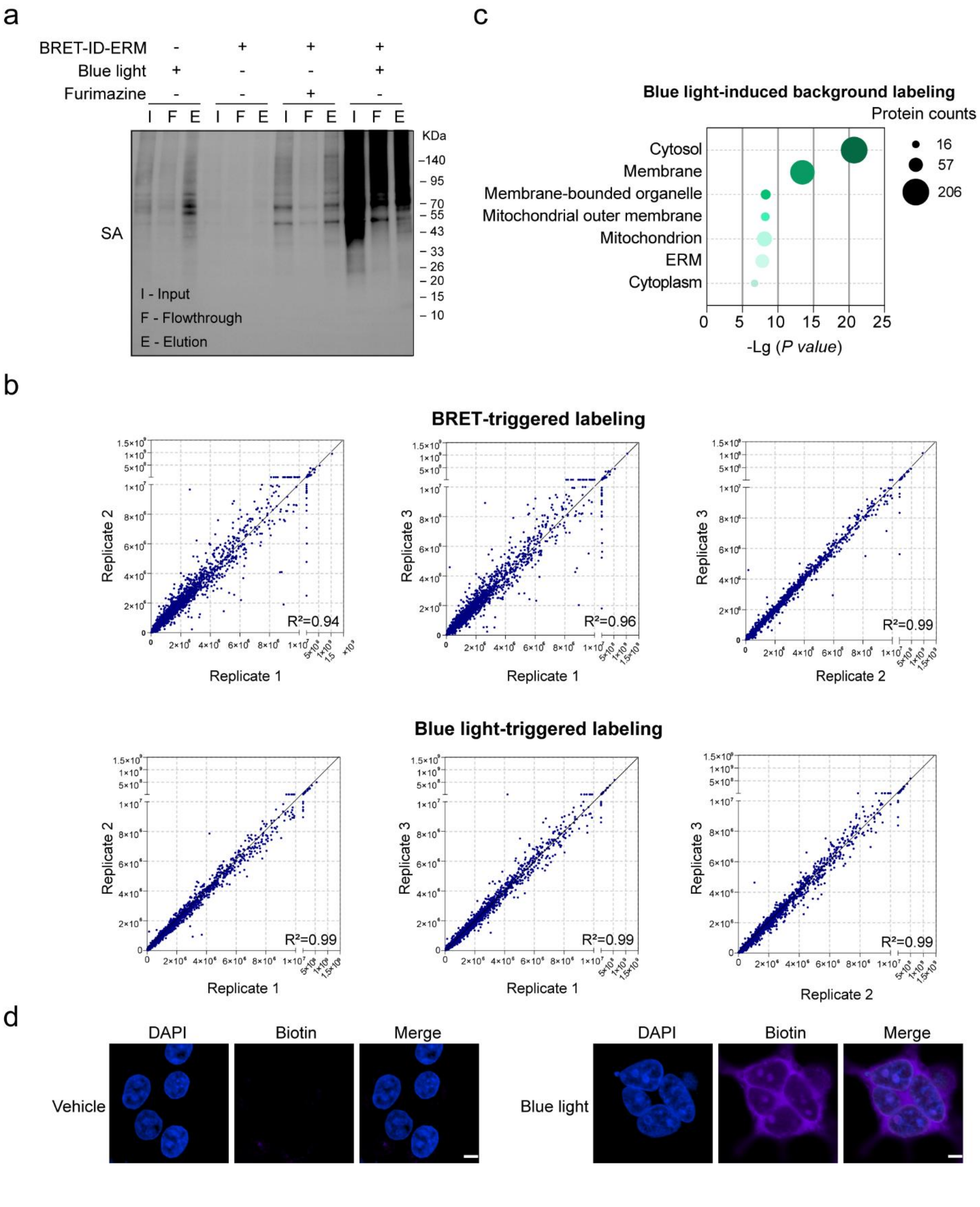

a
BRET-ID-ERM
Blue light
Furimazine
I F E I F E I F E I F E
KDa
140
95
70
55
43
33
26
20
15
10
SA
I - Input
F - Flowthrough
E - Elution
c
Blue light-induced background labeling
Protein counts
16
57
206
Cytosol
Membrane
Membrane-bounded organelle
Mitochondrial outer membrane
Mitochondrion
ERM
Cytoplasm
0 5 10 15 20 25
-Lg (P value)
b
BRET-triggered labeling
Replicate 2
Replicate 1
R²=0.94
Replicate 3
Replicate 1
R²=0.96
Replicate 3
Replicate 2
R²=0.99
Blue light-triggered labeling
Replicate 2
Replicate 1
R²=0.99
Replicate 3
Replicate 1
R²=0.99
Replicate 3
Replicate 2
R²=0.99
d
DAPI
Biotin
Merge
Vehicle
Blue light

Figure S3. Proteomic mapping of ER membrane proteins by BRET-ID. a. Validation of BRET-ID-ERM labeling and streptavidin enrichment. The labeled cells were lysed and subjected to click reaction with azide-biotin. The resulted lysates (i.e., input; labeled as 'I') were incubated with streptavidin beads to capture biotinylated proteins. After enrichment, the supernatant (i.e., flow-through; labeled as 'F') was collected and the biotinylated proteins (i.e., eluates; labeled as 'E') were eluted by beads boiling. All samples were analyzed by streptavidin blotting. **b.** Correlation of protein intensities across biological replicates for BRET-triggered (upper) and blue light-triggered (lower) labeling. **c**. Gene Ontology (GO) cellular component analysis of enriched proteins by blue light-induced background labeling. **d.** Confocal fluorescence imaging of light-induced background labeling. HEK293T cells were treated with alkyne-aniline and irradiated by blue light. Cells were fixed and labeled with azide-biotin, followed by staining with Streptavidin-AF647 for visualization of labeled proteins. Scale bars, 5 μm.

a

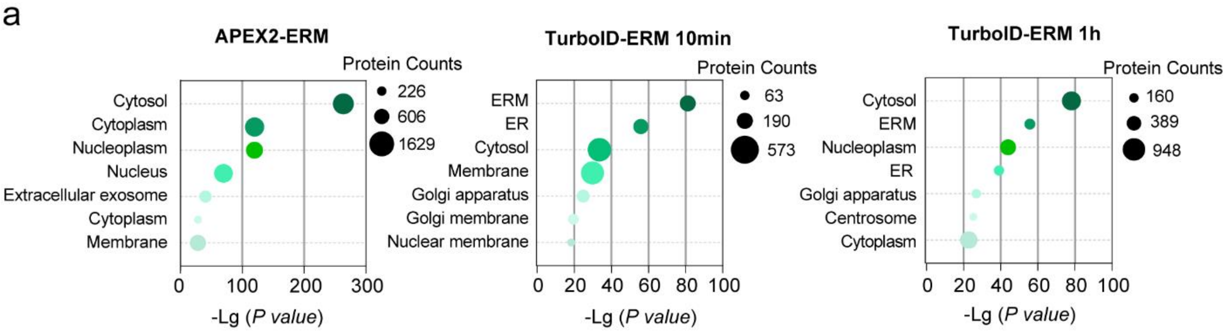

c

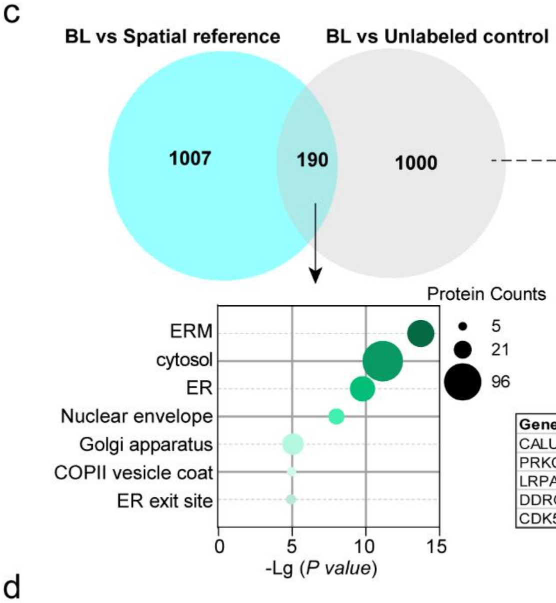

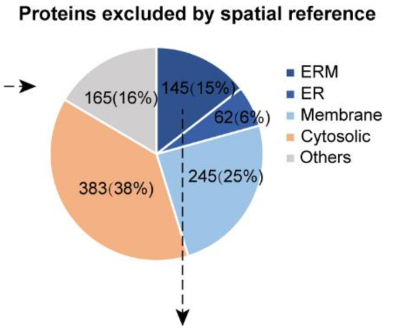

Gene symbol	Protein name	Subcellular localization
CALU	Calumenin	ERM, Golgi, Melanosome
PRKCSH	Glucosidase 2 subunit beta	ER
LRPAP1	Alpha-2-macroglobulin receptor-associated protein	ER, Golgi, Endosome, cell surface
DDRGK1	DDRGK domain-containing protein 1	ERM
CDK5RAP3	CDK5 regulatory subunit-associated protein 3	ERM,Cytoplasm,Nucleus,Centrosome

d

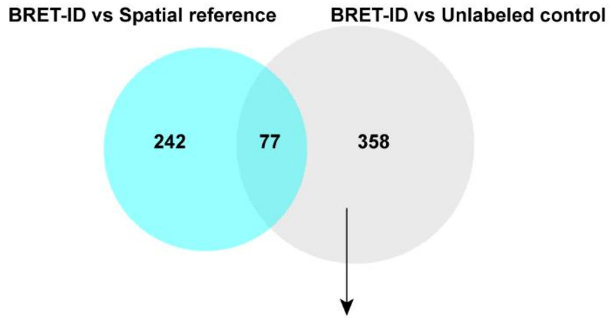

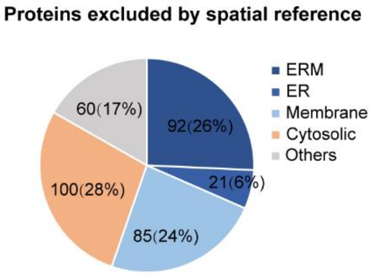

Gene symbol	Protein name	Subcellular localization
AGPAT1	Lysophosphatidic acid acyltransferase alpha	ERM
KDSR	3-ketodihydrosphingosine reductase	ERM
ALG6	Asparagine-linked glycosylation protein 6 homolog	ERM
ATP6AP1	V-type proton ATPase subunit S1	ERM, Cytoplasm, Golgi
ATP2A2	Calcium pump 2	ERM

e

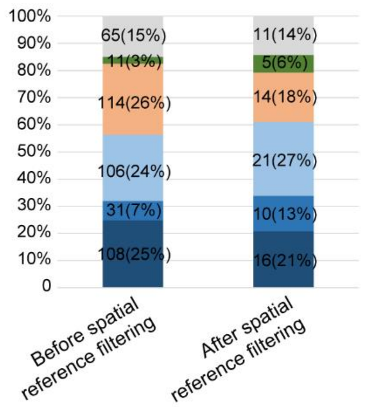

Figure S4. Comparison of ERM proteomes identified by BRET-ID and other proximity labeling methods. a. GO cellular component analysis of ERM proteomes identified by APEX2 and TurboID before filtering against spatial references. The APEX2-ERM dataset is from Supplementary file 1 of Hung et al[3]. The TurboID-ERM datasets with 10 and 60 minutes of biotin treatment from supplementary table 5 of Branon et al[2]. **b.** Filtering against the spatial reference results a more specific ERM dataset. The upper panel shows the overlap of proteins enriched in the comparison of blue light labeling samples against the spatial reference and the unlabeled control. The lower panel shows the GO cellular component analysis of the 190 overlapped proteins. **c.** The subcellular distribution of 1000 enriched proteins excluded by the spatial reference filtering. Representative ER-related proteins that are excluded by the spatial reference filtering are shown with UniProt-annotated subcellular localizations. **d**. Subcellular distribution of the 358 BRET-ID-enriched proteins excluded by spatial reference filtering. Representative ER-related proteins excluded by filtering are shown, along with their UniProt-annotated subcellular localizations. **e** Subcellular distribution of BRET-ID-enriched proteins before versus after spatial reference filtering.

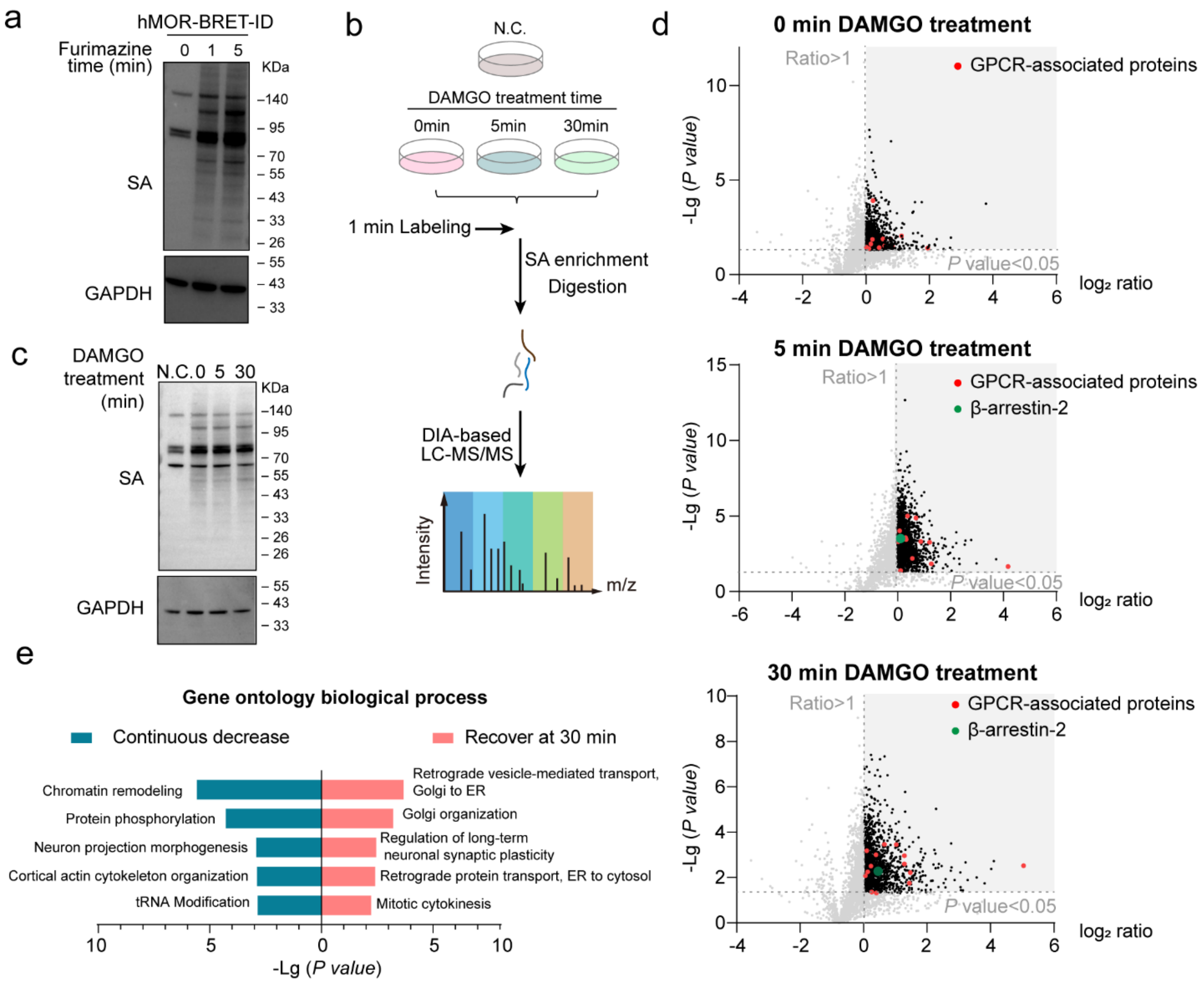

Figure S5. Precise proteomic profiling of hMOR-interacting proteins using BRET-ID. a. Furimazine-dependent hMOR-BRET-ID labeling. HEK293T cells expressing hMOR-BRET-ID were incubated with 500 μM biotin-aniline for 30 minutes, followed by the addition of 75 μM furimazine for 1 minute. **b.** Design of DIA-based proteomics for mapping hMOR-interacting proteins using BRET-ID. **c.** hMOR-BRET-ID labeling at various DAMGO treatment durations. HEK293T cells expressing hMOR-BRET-ID were stimulated with 10 μM DAMGO for 0, 5 or 30 minutes, followed by 1-minute of furimazine treatment to initiate BRET-ID labeling. In **a** and **c**, biotinylation was assessed by streptavidin blotting. **d**. Volcano plots showing the enrichment of labeled proteins by hMOR-BRET-ID under different DAMGO treatment times. Proteins that

are significantly enriched in the labeled samples (p-value < 0.05) are highlighted, with known hMOR related proteins in red and β-arrestin-2 in green. **e**. GO biological process analysis of protein clusters showing decreased interactions with hMOR after 5 minutes of DAMGO treatment. The left panel illustrates the "continuous decrease" protein cluster, which maintains low-level hMOR interactions at 30 minutes of DAMGO treatment, while the right panel shows the "recovering" protein cluster, which exhibits increased hMOR interactions at 30 minutes of DAMGO treatment.

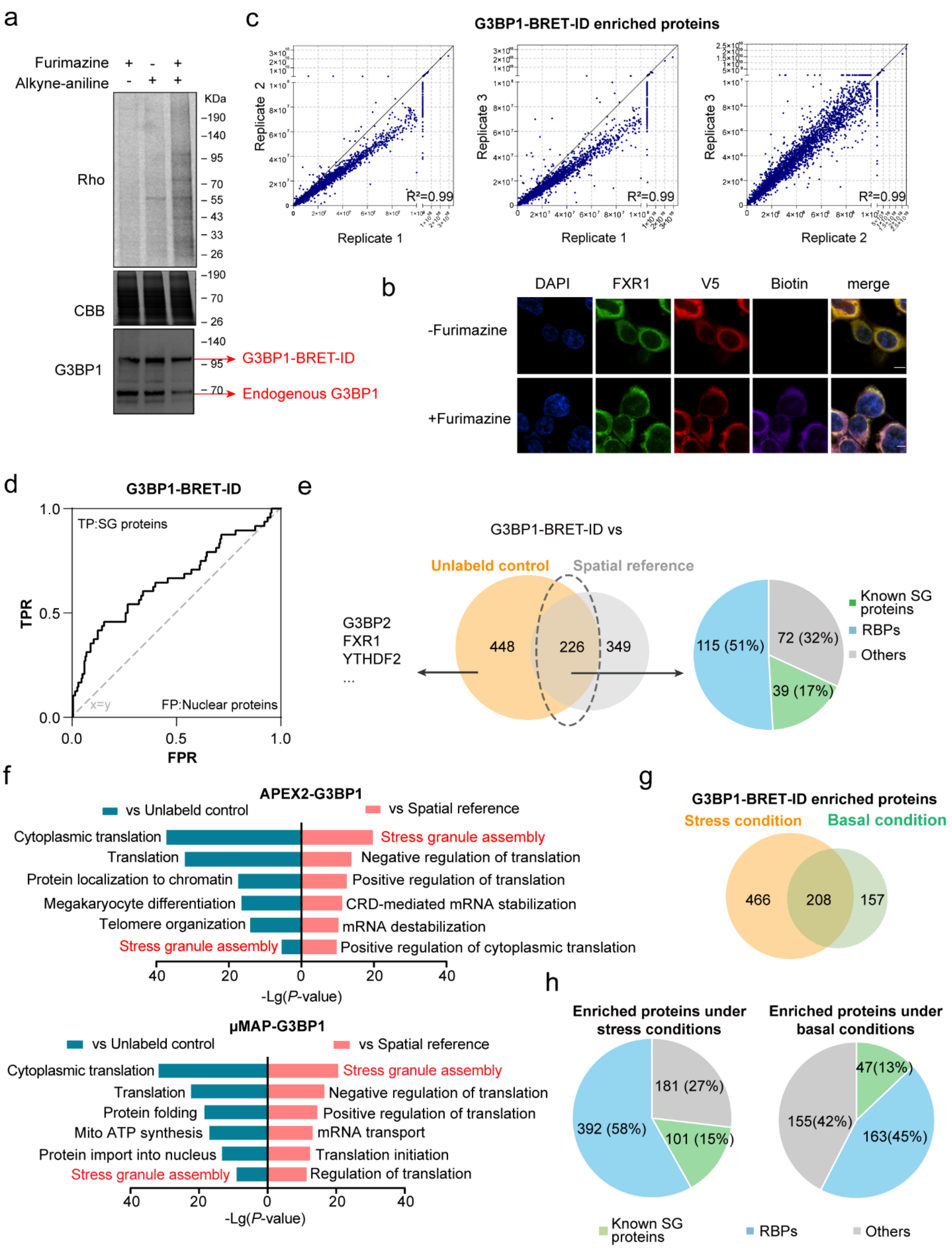

a
Furimazine + - +
Alkyne-aniline - + +
KDa
Rho
CBB
G3BP1
G3BP1-BRET-ID
Endogenous G3BP1
c
G3BP1-BRET-ID enriched proteins
Replicate 2
Replicate 1
Replicate 3
Replicate 1
Replicate 3
Replicate 2
R²=0.99
b
DAPI
FXR1
V5
Biotin
merge
-Furimazine
+Furimazine
d
G3BP1-BRET-ID
TP:SG proteins
FP:Nuclear proteins
x=y
TPR
FPR
e
G3BP1-BRET-ID vs
Unlabeld control
Spatial reference
448
226
349
G3BP2
FXR1
YTHDF2
...
115 (51%)
72 (32%)
39 (17%)
Known SG proteins
RBPs
Others
f
APEX2-G3BP1
vs Unlabeld control
vs Spatial reference
Cytoplasmic translation
Translation
Protein localization to chromatin
Megakaryocyte differentiation
Telomere organization
Stress granule assembly
Stress granule assembly
Negative regulation of translation
Positive regulation of translation
CRD-mediated mRNA stabilization
mRNA destabilization
Positive regulation of cytoplasmic translation
-Lg(P-value)
μMAP-G3BP1
vs Unlabeld control
vs Spatial reference
Cytoplasmic translation
Translation
Protein folding
Mito ATP synthesis
Protein import into nucleus
Stress granule assembly
Stress granule assembly
Negative regulation of translation
Positive regulation of translation
mRNA transport
Translation initiation
Regulation of translation
-Lg(P-value)
g
G3BP1-BRET-ID enriched proteins
Stress condition
Basal condition
466
208
157
h
Enriched proteins under stress conditions
Enriched proteins under basal conditions
392 (58%)
181 (27%)
101 (15%)
47(13%)
155(42%)
163(45%)
Known SG proteins
RBPs
Others

Figure S6. Precise proteomic profiling of stress granules using BRET-ID. a. In-gel fluorescence of furimazine-dependent G3BP1-BRET-ID labeling. HEK293T cells stably expressing G3BP1-BRET-ID were incubated with alkyne-aniline for 15mins, followed by 1-minute furimazine-based BRET-ID labeling. The cells were lysed and click reacted with azide-Rhodamine, followed by in-gel fluorescence scanning. Coomassie Brilliant Blue (CBB) staining was used to confirm equal protein loading and anti-G3BP1 blotting detects both endogenous and BRET-ID-tagged G3BP1. **b**. Confocal fluorescence imaging of BRET-ID labeling in SGs under basal condition. HEK293T cells stably expressing G3BP1-BRET-ID were incubated with alkyne-aniline for 15mins, followed by 1-minute furimazine-based BRET-ID labeling and imaging. Streptavidin-AF647 labels biotinylated proteins, while anti-V5 staining shows enzyme expression. FXR1 marks the stress granules. Scale bars, 5 μm. **c.** Correlation of protein intensities across biological replicates for G3BP1-BRET-ID labeling. **d**. Receiver operating characteristic (ROC) curves for G3BP1-BRET-ID labeling. Proteins are ranked in descending order based on enrichment ratios. True positives are known SG proteins, while false positives are annotated nuclear proteins. **e**. Filtering against the spatial reference does not increase the specificity of BRET-ID-identified SG dataset and decrease its sensitivity. The left panel shows the overlap of proteins enriched in the comparison of G3BP1-BRET-ID samples against the spatial reference and the unlabeled control. The right panel shows the percentages of known SG proteins and RNA-binding proteins in the 226 overlapped proteins. Well-established SG proteins such as G3BP2, FXR1 and YTHDF2 are excluded after filtering against the spatial reference. **f**. GO biological process analysis of proteins identified by APEX2 and μMAP before and after filtering against spatial references. The APEX2-G3BP1 datasets are from supplementary table S1 of Kollet et al[4]. The μMAP -G3BP1 datasets are from supplementary data 1 of Pan et al[5]. **g**. Overlap of G3BP1-BRET-ID-enriched proteins under basal and stress conditions. **h.** Percentages of known stress granule proteins and RNA-binding proteins in G3BP1-BRET-ID-enriched proteins under the stress or basal condition.

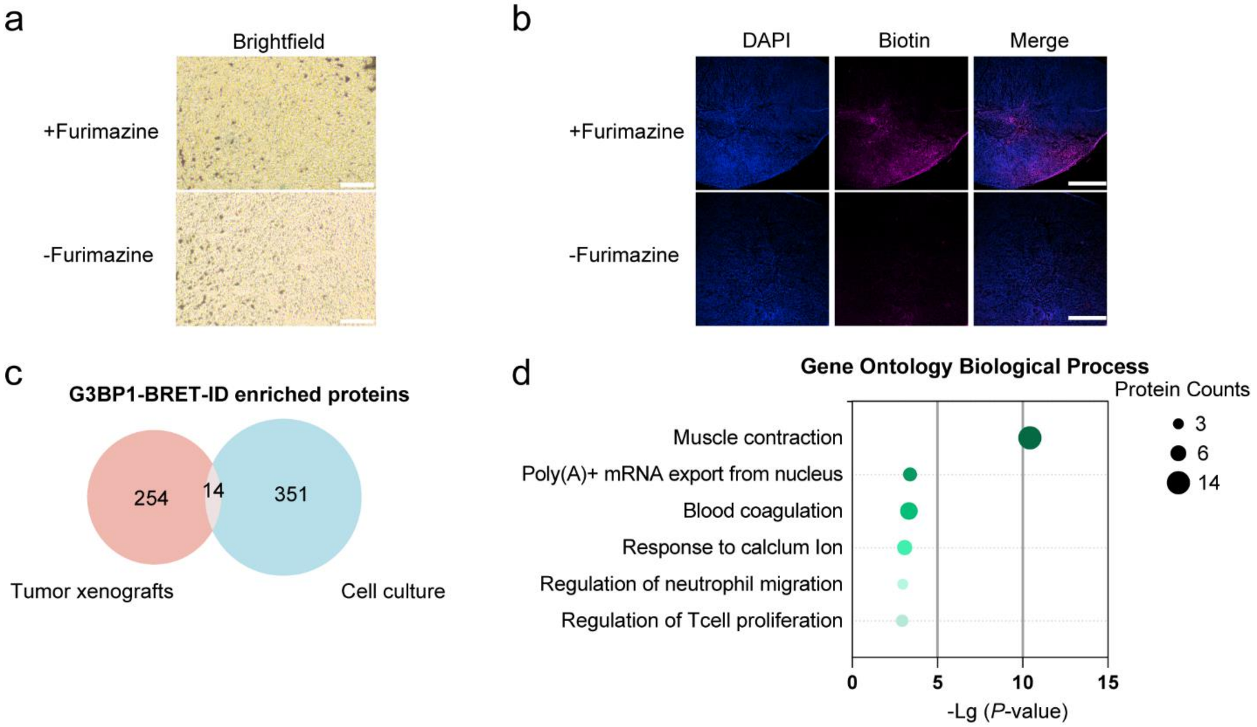

Figure S7. Additional analysis of G3BP-interacting proteins identified by *in vivo* BRET-ID labeling. a. Tumor samples were embedded, cryosectioned into 10-μm-thick slices, and mounted onto glass slides for bright-field imaging. **b**. Immunofluorescence analysis of BRET-ID labeling in tumor tissues. Tissue sections underwent heat-mediated antigen retrieval, followed by click-labeling with azide-biotin. Staining with DAPI (nuclei) and streptavidin-AF647 (labeling signals) was performed to visualize spatial biotinylation patterns. Scale bars, 200 μm. **c**. Overlap of G3BP1-BRET-ID-enriched proteins in tumor xenografts and cell culture. **d**. GO biological process analysis of G3BP1-BRET-ID-enriched proteins in tumor xenografts.

Supplementary Tables

Table S1. ERM proteins enriched by BRET- or blue light-based photocatalytic labeling. Related to figure 3 and figure S3. The ERM proteins enriched by BRET-ID are shown in Tab 1, while those enriched by blue light are shown in Tab 2. The mean values of ratios and *p*-values were calculated based on three replicates. In both tabs, unlabeled samples were used as negative controls for comparisons. True positive list (ERM-localized proteins) and False positive list (mitochondrial matrix proteins) for ROC analysis are shown in Tab 3.

Table S2. Filtering of blue light-based ERM labeling against the cytosolic spatial reference. Related to figure 3 and figure S4. Proteins enriched by blue light-induced background labeling are presented in Tab 1. Proteins enriched in the comparison of blue light-based ERM labeling against the cytosolic spatial reference are presented in Tab 2. The mean values of ratios and *p*-values were calculated based on three replicates. After spatial reference filtering, the final list of ERM proteins identified by blue light-based labeling are shown in Tab 3. The blue light-activated ERM labeling samples were compared with both the unlabeled control and the spatial reference. Two lists were generated based on significant enrichment comparisons (ratio>1, *p*-value<0.05). The overlapping portion between the two lists represents the final list. True positive list (ERM-localized proteins) and False positive list (non-secretory proteins) for ROC analysis are shown in Tab 4.

Table S3. The hMOR interactome identified by BRET-ID under different DAMGO treatment conditions. Related to figure 4 and figure S5. Tab 1 shows the ratios of proteins at 0, 5, and 30 minutes of DAMGO treatment. Proteins that exhibit significant enrichment (ratio>1, *p*-value<0.05) at least at one time point are shown. The mean values of ratios and *p*-values were calculated based on three replicates. True positive list (plasma membrane proteins) and False positive list (nuclear proteins) are shown in Tab 2.

Table S4. G3BP1-BRET-ID enriched proteins under stress or basal conditions. Related to figure 5 and figure S6. Proteins enriched by G3BP1-BRET-ID under stress and basal conditions are presented in Tab 1 and Tab 2, respectively. The mean values of ratios and *p*-values were calculated based on three replicates. True positive list (stress granule proteins) and False positive list (nuclear proteins) for ROC analysis are shown in Tab 3.

Table S5. G3BP1-BRET-ID enriched proteins in tumor xenografts. Related to figure 6. G3BP1 interaction proteins identified by BRET-ID in the xenograft model are presented in Tab 1. The mean values of ratios and *p*-values were calculated based on three replicates.

Table S6. List of fusion constructs used in this study.

Names	Features	Vector	Details
cpNluc-HT	HA-HaloTag$_{(4-154)}$-NanoLuc$_{(66-171)}$-GGTGGSGGTGGS-NanoLuc$_{(1-65)}$-HaloTag$_{(156-297)}$	pcDNA 3.1	HaloTag$_{(4-154)}$ indicates the amino acid sequence of HaloTag from position 4 to position 154: IGTGFPFDPHYVEVLGERMHYVDVGPRDGTPVLFLHGNPTSSYVWRNIIPHVAPTHRCIAPDLIGMGKSDKPDLGYFFDDHVRFMDAFIEALGLEEVVLVIHDWGSALGFHWAKRNPERVKGIAFMEFIRPIPTWDEWPEFARETFQAFRT

			HaloTag$_{(156-297)}$: DVGRKLIIDQNVFIEGTLPMGVVR PLTEVEMDHYREPFLNPVDREPLW RFPNELPIAGEPANIVALVEEYMD WLHQSPVPKLLFWGTPGVLIPPAE AARLAKSLPNCKAVDIGPGLNLLQ EDNPDLIGSEIARWLSTLEISG<br>NanoLuc$_{(1-65)}$: MVFTLEDFVGDWRQTAGYNLDQ VLEQGGVSSLFQNLGVSVTPIQRIV LSGENGLKIDIHVIIPYE<br>NanoLuc$_{(66-171)}$: GLSGDQMGQIEKIFKVVYPVDDH HFKVILHYGTLVIDGVTPNMIDYF GRPYEGIAVFDGKKITVTGTLWNG NKIIDERLINPDGSLLFRVTINGVTG WRLCERILA<br>HA: YPYDVPDYA
NanoLuc	HA-NanoLuc	pFN31K	
Nluc-15-miniSOG/SOPP/SOPP3	V5-NanoLuc-15 aa linker-miniSOG/SOPP/SOPP3	pCMV	The protein sequence of NanoLuc is from Hall et al.[6] The protein sequences of miniSOG, SOPP and SOPP3 are from Westberg et al.[7]<br>V5: GKPIPNPLLGLDST

			15 aa linker: GDPLVQCGGIAGSAT<br>NanoLuc-15-SOPP3 is the final version used for BRET-ID.
Nluc-5/12/20/30-LOV	V5-NanoLuc-5/12/20/30 aa linker-LOV	pCMV	5 aa linker: GDPLV<br>12 aa linker: TSGSPGLQEFGT<br>20 aa linker:<br>LEYDVPDYAKLYPYDVPDYA<br>30 aa linker:<br>GDPLVQCGGIAGSATGDPLVQCG GIAGSAT
BRET-ID-ERM	V5- BRET-ID -linker-Sec61β	pCMV	Linker: GGSGGGGSGGGGSNSRV<br>Sec61β:<br>PGPTPSGTNVGSSGRSPSKAVAAR AAGSTVRQRKNASCGTRSAGRTTS AGTGGMWRFYTEDSPGLKVGPVP VLVMSLLFIASVFMLHIWGKYTRS
BRET-ID-NLS	V5- BRET-ID – NLS	pcDNA 3.1	NLS:<br>AEFSRADPKKKRKVDPKKKRKVD PKKKRKV
BRET-ID-OMM	V5- BRET-ID -MAVS	pcDNA 3.1	MAVS:<br>ADAEFRPSPGALWLQVAVTGVLV VTLLVVLYRRRLH

BRET-ID-mito matrix	COX-V5-BRET-ID	PcDNA 3.1	COX: MLATRVFSLVGKRAISTSVCVRAH
Flag-hMOR	Signal peptide-Flag-hMOR	pcDNA 3.1	Signal peptide: MKTIIALSYIFCLVFA<br>The protein sequence of hMOR is from UniProt
Flag-hMOR-BRET-ID	Signal peptide-Flag-Hmor-BRET-ID	pcDNA 3.1	BRET-ID is directly fused to the sequence mentioned above.
G3BP1-BRET-ID	G3BP1-V5-BRET-ID	PXL304	The protein sequence of G3BP1 is from UniProt.<br>This lentiviral vector carries a blasticidin resistance marker.

References

1. Manglik, A. et al. Structure-based discovery of opioid analgesics with reduced side effects. *Nature* **537**, 185-190 (2016).
2. Branon, T.C. et al. Efficient proximity labeling in living cells and organisms with TurboID. *Nat. Biotechnol.* **36**, 880-887 (2018).
3. Hung, V. et al. Proteomic mapping of cytosol-facing outer mitochondrial and ER membranes in living human cells by proximity biotinylation. *Elife* **6** (2017).
4. Marmor-Kollet, H. et al. Spatiotemporal Proteomic Analysis of Stress Granule Disassembly Using APEX Reveals Regulation by SUMOylation and Links to ALS Pathogenesis. *Mol. Cell* **80**, 876-891.e876 (2020).
5. Pan, C.R., Knutson, S.D., Huth, S.W. & MacMillan, D.W.C. µMap proximity labeling in living cells reveals stress granule disassembly mechanisms. *Nat. Chem. Biol.* (2024).
6. Hall, M.P. et al. Engineered luciferase reporter from a deep sea shrimp utilizing a novel imidazopyrazinone substrate. *ACS Chem. Biol.* **7**, 1848-1857 (2012).
7. Westberg, M., Bregnhøj, M., Etzerodt, M. & Ogilby, P.R. No Photon Wasted: An Efficient and Selective Singlet Oxygen Photosensitizing Protein. *J. Phys. Chem. B* **121**, 9366-9371 (2017).
