# Supplementary material for: Precise and In Vivo-Compatible Spatial Proteomics via Bioluminescence-Triggered Photocatalytic Proximity Labeling
Source: ACS Cent Sci. 2025 Jul 30;11(9):1611–26. doi: 10.1021/acscentsci.5c00520 (PMC12464768; doi:10.1021/acscentsci.5c00520)
Supplement: Supplementary file 7 [file oc5c00520_si_007.pdf]

oc-2025-005209.R1

Name: Peer Review Information for "Precise and in vivo-compatible spatial proteomics via bioluminescence-triggered photocatalytic proximity labeling"

First Round of Reviewer Comments

Reviewer: 1

Comments to the Author

This manuscript presents a highly innovative and impactful proximity labeling (PL) strategy, BRET-ID, which leverages genetically encoded bioluminescence resonance energy transfer (BRET) to drive photocatalytic protein labeling in living cells and organisms. The authors successfully address several key limitations of existing photocatalytic PL methods—namely toxicity, light-induced background, and poor in vivo applicability—by creating a new genetically encoded platform capable of sub-minute temporal resolution and high spatial precision.

The BRET-ID approach is conceptually elegant and technically well-executed. Its broad utility is demonstrated across diverse biological contexts, including endoplasmic reticulum membrane (ERM) protein profiling, dynamic GPCR endocytosis, and stress granule biology. Notably, the authors apply BRET-ID in tumor xenograft models, highlighting its strong potential for in vivo proteomic applications.

Overall, this work represents a valuable contribution to the molecular toolkit for studying protein localization and interaction dynamics in live systems. Its versatility and ease of genetic encoding will likely draw widespread interest from researchers in chemical biology, cell biology, and biomedical research. I strongly recommend publication in ACS Central Science after revisions addressing the following points:

Mayjor Concerns:

1. The authors' data show that light-induced background labeling results in the enrichment of multiple ERM proteins. Have the authors attempted to directly visualize this background labeling using other strategies like fluorescence imaging? Such data could help clarify whether the background labeling has a specific subcellular distribution, which would aid future users in interpreting their datasets.

2. The identification of RICTOR in SGs is intriguing. However, this conclusion would be strengthened by orthogonal evidence—e.g., loss-of-function experiments testing whether RICTOR contributes functionally to SG formation or dynamics.

3. While the main focus is on stress-induced SG formation, the G3BP1 interactome under basal conditions also represents a valuable and underexplored dataset. A more detailed analysis of this protein list—such as assessing the proportion of RNA-binding proteins and known stress granule components—would help clarify the baseline interaction landscape of G3BP1.

Minor Concerns:

1. The authors state that BRET-ID loses enzymatic activity approximately 5 minutes after activation. It would be useful to determine whether this is due to the intrinsic photobleaching of the system or cofactor availability. Specifically, since miniSOG activity depends on FMN, could local depletion of FMN contribute to the observed loss of activity? Have the authors tested whether supplementing exogenous FMN rescues BRET-ID activity?

2. Based on the presented data, BRET-ID exhibits excellent labeling specificity, but the overall labeling signal appears relatively weak compared to conventional light-activated methods. While this likely contributes to reduced background, it may also limit sensitivity. A more detailed discussion of this tradeoff—particularly regarding applications where stronger labeling might be necessary—would help guide future users in choosing or adapting the method appropriately.

Reviewer: 2

Comments to the Author

Reviewer Report

This manuscript introduces BRET-ID, a novel proximity labeling (PL) technique that addresses limitations of current PL methods. By fusing NanoLuc luciferase with SOPP3, a photosensitizer, the authors enable bioluminescence resonance energy transfer (BRET) to trigger protein labeling without exogenous light or hydrogen peroxide. The technology demonstrates excellent spatial and temporal resolution across several biological applications, including mapping ER membrane proteins, GPCR dynamics, and stress granule components in both cultured cells and tumor xenografts. The authors provide a well-structured account of their research objectives, methodologies, and findings. This work addresses a significant need for non-toxic PL methods compatible with in vivo applications. BRET-ID has high spatiotemporal resolution, minimal background, self-termination, and demonstrated in vitro and in vivo applications. Overall, the key conclusions drawn in the paper are convincing and supported by their experimental data, though a few items need clarification. Specifically:

Major Concerns

1. Insufficient comparison from related work: The manuscript didn’t adequately explain the distinction between BRET-ID and the recently published APEX2-SOPP3 system (doi: 10.1038/s41422-024-01061-9). The authors should explicitly address:

- Why SOPP3 alone works in BRET-ID but needed APEX2 in previous work
- How the alkyne-aniline probe enables direct labeling compared to biotin-phenol
- The mechanistic differences in protein labeling between the two approaches

2. The authors make a significant claim that BRET-ID eliminates the need for spatial references in proximity labeling experiments. Additional discussion is needed about the contexts where this applies, or if they want to generalize this claim, at least one more compartment dataset supporting this assertion would be beneficial. It's unclear if the stress granule data also supports this claim, and this should be addressed. Maybe by using a similar ROC curve analysis.

Minor Concerns

1. I would recommend putting the current fig 1 into SI. NLuc-Halotag seems like an inferior design compared to NLuc-LOV/SOPP3. I would try not to confuse the readers and have them focus on the better design.

2. Fig 1f, lane 1 from the left: how could a no-enzyme control have HA staining?

3. Fig S2e left panel, why is BRET with enzyme weaker than no enzyme control?

4. In Figure 2, the optimization process for NanoLuc-SOPP3 linker design is described, but the rationale for choosing the rigid 15aa linker needs more detail.

5. Supplementary Figure 2c compares alkyne-aniline vs. alkyne-phenol probes, but lacks a detailed explanation for the superior performance of alkyne-aniline, which appears to be critical for the success of BRET-ID without APEX2.

6. I recommend showing colocalization of V5 and biotin in Fig 2i similar to Fig S2h to demonstrate the better spatial resolution of BRET-ID.

7. Page 17, line 47/48, reference superscript.

8. For the tumor labeling experiment, it would be great to show a cross section of the tumor post-labeling between two groups to demonstrate the penetration of substrates and the coverage of the tissue.

Overall, this work represents a significant advance in proximity labeling technology with particular value for in vivo applications. With appropriate revisions addressing the above-mentioned comments, this manuscript would merit publication in ACS Central Science.

Reviewer: 3

Comments to the Author

This manuscript presents an innovative and timely proximity labeling platform, BRET-ID, which combines genetic encoding, oxidative precision, and in vivo compatibility without reliance on exogenous light or hydrogen peroxide. The authors successfully demonstrate labeling in diverse compartments and deliver the first proteomic map of stress granules in live tumor tissue. The study is compelling in scope, carefully executed, and clearly written. For the publication of this manuscript in ACS Central Science, I recommend the following revisions:

Major comments

1. A key promise of BRET-ID is its broad applicability to organelles. However, labeling was not tested in the mitochondrial matrix, which poses a unique challenge due to its compartmentalization and the need for furimazine to cross the inner mitochondrial

membrane. A matrix-targeted NanoLuc-SOPP3 construct should be tested to determine whether BRET-ID is functional in this topologically enclosed environment. Even a negative result would be informative and help define the method’s boundaries.

2. In the tumor xenograft model, proximity labeling by G3BP1-BRET-ID was analyzed without an in vivo cytosolic reference control. Including a tumor-expressed cytosolic BRET-ID control would help establish the specificity of the G3BP1 interactome and distinguish it from background cytosolic labeling, especially under tumor stress conditions. This control would mirror the in vitro controls and improve confidence in the in vivo interactome conclusions.

3. The manuscript uses different enrichment ratio thresholds (e.g., ratio >1 vs. >2) to define significantly labeled proteins across experiments, but the rationale behind these choices is unclear. Since these cutoffs influence the composition and interpretation of reported interactomes (e.g., for G3BP1 in vitro vs. tumor), I recommend that the authors briefly justify the threshold used in each context and whether these were empirically determined based on signal-to-noise or statistical robustness.

Minor comments

4. Figure 2D includes an alkyne-phenol structure, which does not appear to be used in the study. Since the text and data focus exclusively on alkyne-aniline, I suggest removing the phenol structure to prevent confusion unless experimental data using it are presented.

5. The authors may consider mentioning the iAPEX method (Sroka et al., bioRxiv, 2025), which also enables in vivo oxidative labeling by local hydrogen peroxide generation via co-expresion of d-amino acid oxidase. Although mechanistically distinct, both systems share a goal of enabling safe, precise proximity labeling in live organisms. A short reference in the Discussion would help readers situate BRET-ID within the broader context of emerging in vivo PL platforms.

Author's Response to Peer Review Comments:

Dear Editor,

Below are our point-to-point responses to the questions and concerns raised by the reviewers (colored in blue). The newly added or revised texts have also been colored in yellow in the manuscript files to facilitate the second round of review.

Reviewer(s)' Comments to Author:

Reviewer: 1

Recommendation: Publish in ACS Central Science after minor revisions noted.

Comments:

This manuscript presents a highly innovative and impactful proximity labeling (PL) strategy, BRET-ID, which leverages genetically encoded bioluminescence resonance energy transfer (BRET) to drive photocatalytic protein labeling in living cells and organisms. The authors successfully address several key limitations of existing photocatalytic PL methods—namely toxicity, light-induced background, and poor in vivo applicability—by creating a new genetically encoded platform capable of sub-minute temporal resolution and high spatial precision.

The BRET-ID approach is conceptually elegant and technically well-executed. Its broad utility is demonstrated across diverse biological contexts, including endoplasmic reticulum membrane (ERM) protein profiling, dynamic GPCR endocytosis, and stress granule biology. Notably, the authors apply BRET-ID in tumor xenograft models, highlighting its strong potential for in vivo proteomic applications.

Overall, this work represents a valuable contribution to the molecular toolkit for studying protein localization and interaction dynamics in live systems. Its versatility and ease of genetic encoding will likely draw widespread interest from researchers in chemical biology, cell biology, and biomedical research. I strongly recommend publication in ACS Central Science after revisions addressing the following points:

Response: We thank the reviewer for the positive comments.

Mayjor Concerns:

1. The authors' data show that light-induced background labeling results in the enrichment of multiple ERM proteins. Have the authors attempted to directly visualize this background labeling using other strategies like fluorescence imaging? Such data could help clarify whether the background labeling has a specific subcellular distribution, which would aid future users in interpreting their datasets.

Response: We thank the reviewer for this insightful suggestion. Following their recommendation, we conducted confocal imaging to assess light-induced background labeling (**Rebuttal Figure 1, now included as Figure S3d in the revised manuscript**). The imaging results corroborate our proteomic data, which revealed non-specific labeling of proteins across diverse subcellular compartments, including the ER.

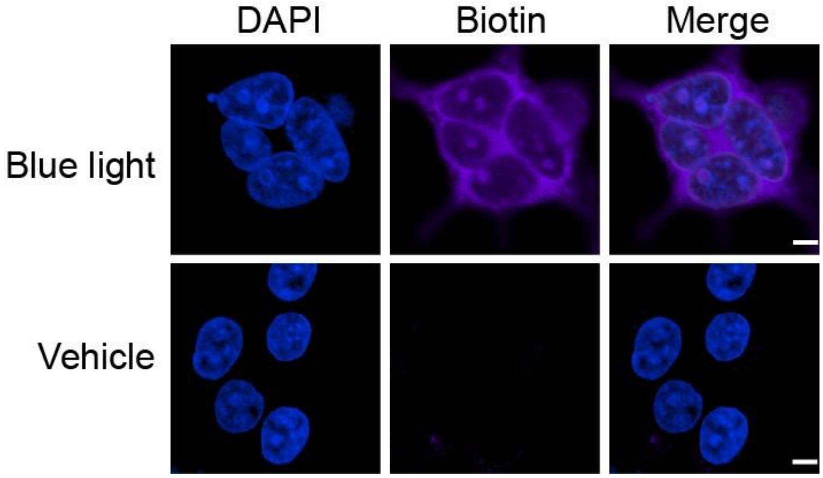

Rebuttal Figure 1. Confocal fluorescence imaging of light-induced background labeling. HEK293T cells were treated with alkyne-aniline and irradiated by blue light. Cells were fixed and labeled with azide-biotin, followed by staining with Streptavidin-AF647 for visualization of labeled proteins. Scale bars, 5 μm.

2. The identification of RICTOR in SGs is intriguing. However, this conclusion would be strengthened by orthogonal evidence—e.g., loss-of-function experiments testing whether RICTOR contributes functionally to SG formation or dynamics.

Response: We thank the reviewer for their thoughtful suggestion. As proposed, we performed confocal imaging to evaluate RICTOR's role in stress granule (SG) assembly during arsenite stress in mammalian cells. HEK293T cells were pretreated with 10 μM of the selective RICTOR inhibitor JR-AB2-011[1], followed by arsenite exposure. Imaging revealed that RICTOR inhibition significantly disrupted SG formation (**Rebuttal Figure 2, now designated as Figure 5i in the revised manuscript**), underscoring its critical role in this process.

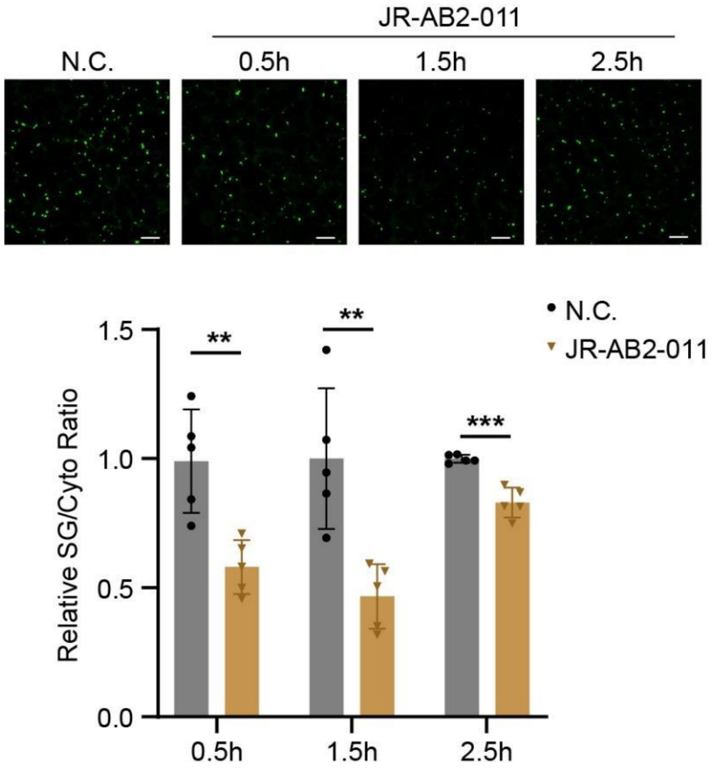

Rebuttal Figure 2. Confocal fluorescence imaging of SG formation under RICTOR inhibition. G3BP1-GFP knock-in HEK293T cells were pretreated with 10 μM of the selective RICTOR inhibitor JR-AB2-011 for the indicated durations, followed by 500 μM of arsenite treatment for 30 minutes. The Relative ratios of G3BP1 in the SG versus in the cytosol were calculated based on five different areas per well. Scale bars, 30 μm.

3. While the main focus is on stress-induced SG formation, the G3BP1 interactome under basal conditions also represents a valuable and underexplored dataset. A more detailed analysis of this protein list—such as assessing the proportion of RNA-binding proteins and known stress granule components—would help clarify the baseline interaction landscape of G3BP1.

Response: We thank the reviewer for their insightful suggestion. As proposed, we analyzed G3BP1-interacting proteins under basal conditions for enrichment of RNA-binding proteins (RBPs) and established SG components. Of the 365 G3BP1 interactors identified, 163 (45%) are annotated RBPs and 47 (13%) are known SG-associated proteins—a degree of enrichment comparable to that observed for G3BP1 interactors under arsenite-induced stress (**Rebuttal Figure 3, now Figure S6h in the revised manuscript**). This analysis further highlights the ability of BRET-ID to resolve specific protein-protein interactions within membraneless organelles.

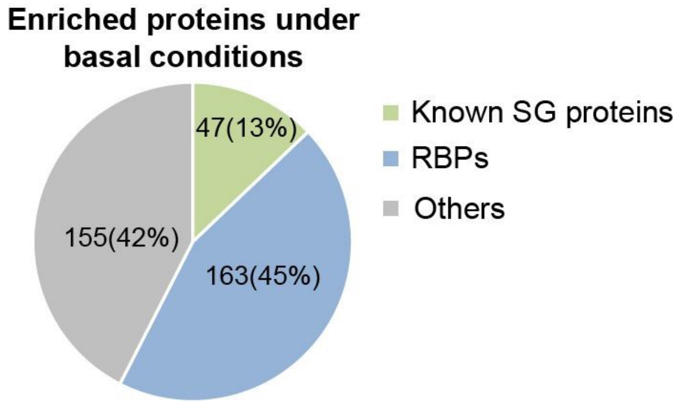

Rebuttal Figure 3. Percentages of known SG proteins and RBPs in BRET-ID-identified G3BP1-interacting proteins under basal condition.

Minor Concerns:

1. The authors state that BRET-ID loses enzymatic activity approximately 5 minutes after activation. It would be useful to determine whether this is due to the intrinsic photobleaching of the system or cofactor availability. Specifically, since miniSOG activity depends on FMN, could local depletion of FMN contribute to the observed loss of activity? Have the authors tested whether supplementing exogenous FMN rescues BRET-ID activity?

Response: We thank the reviewer for pointing out this possibility. As suggested, we supplemented BRET-ID-expressing cells with 150 μM riboflavin, a precursor of FMN, during furimazine treatment. However, this intervention failed to restore BRET-ID activity (**Rebuttal Figure 4, now Figure S2f in the revised manuscript**), effectively ruling out FMN depletion as the cause of enzymatic inactivation. Instead, we propose that oxidation-induced modification of the enzyme itself is the more likely mechanism driving its functional loss.

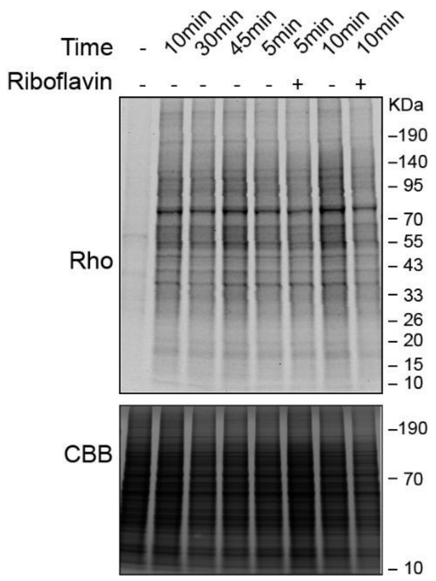

Rebuttal Figure 4. FMN supplement fails to rescue BRET-ID activity. HEK293T cells expressing the BRET-ID construct were pre-incubated with 1 mM alkyne-aniline for 15 minutes, followed by treatment with 75 μM furimazine for 5 minutes, with or without 150 μM riboflavin supplementation.

2. Based on the presented data, BRET-ID exhibits excellent labeling specificity, but the overall labeling signal appears relatively weak compared to conventional light-activated methods. While this likely contributes to reduced background, it may also limit sensitivity. A more detailed discussion of this tradeoff—particularly regarding applications where stronger labeling might be necessary—would help guide future users in choosing or adapting the method appropriately.

Response: We thank the reviewer for this suggestion. As recommended, we have expanded our discussion on the inherent trade-off between specificity and sensitivity in the final paragraph of the Discussion section.

Reviewer: 2

Recommendation: Publish in ACS Central Science after minor revisions noted.

Comments:

Reviewer Report

This manuscript introduces BRET-ID, a novel proximity labeling (PL) technique that addresses limitations of current PL methods. By fusing NanoLuc luciferase with SOPP3, a photosensitizer, the authors enable bioluminescence resonance energy transfer (BRET) to trigger protein labeling without exogenous light or hydrogen peroxide. The technology demonstrates excellent spatial and temporal resolution across several biological applications, including mapping ER membrane proteins, GPCR dynamics, and stress granule components in both cultured cells and tumor xenografts. The authors provide a well-structured account of their research objectives, methodologies, and findings. This work addresses a significant need for non-toxic PL methods compatible with in vivo applications. BRET-ID has high spatiotemporal resolution, minimal background, self-termination, and demonstrated in vitro and in vivo applications. Overall, the key conclusions drawn in the paper are convincing and supported by their experimental data, though a few items need clarification. Specifically:

Response: We thank the reviewer for the positive comments.

Major Concerns

1. Insufficient comparison from related work: The manuscript didn't adequately explain the distinction between BRET-ID and the recently published APEX2-SOPP3 system (doi: 10.1038/s41422-02401061-9). The authors should explicitly address:

o Why SOPP3 alone works in BRET-ID but needed APEX2 in previous work oHow

the alkyne-aniline probe enables direct labeling compared to biotin-phenol o The

mechanistic differences in protein labeling between the two approaches

Response: We sincerely appreciate the reviewer's valuable suggestion. As recommended, we have integrated a comparative discussion of BRET-ID and the APEX2-SOPP3 system into the Discussion section. These two approaches employ fundamentally distinct labeling chemistries:

The APEX2-SOPP3 system (**Rebuttal Figure 5a**) relies on exogenous blue light to activate SOPP3, generating superoxide anion ($O_2^{\bullet -}$) that is enzymatically converted to H_2O_2 via endogenous superoxide dismutase (SOD). This H_2O_2 pool subsequently enables APEX2mediated generation of biotin-phenol radicals, which preferentially label proximal tyrosine residues.

In contrast, BRET-ID (**Rebuttal Figure 5b**) eliminates the need for external illumination by utilizing NanoLuc-derived bioluminescence to activate SOPP3 through a BRET mechanism. The resulting $^1O_2$ directly oxidizes neighboring proteins, with subsequent alkyne-aniline labeling predominantly targeting histidine residues.

Critically, these systems diverge in their terminal enzymatic components: APEX2 executes the final labeling step in the APEX2-SOPP3 strategy, whereas SOPP3 execute the final labeling in BRET-ID. This fundamental difference reflects their distinct design rationales—while the APEX2-SOPP3 strategy was developed to eliminate cytotoxic H_2O_2 dependence inherent to classical APEX2 methods, BRET-ID achieves blue light-independent operation through its bioluminescent mechanism, thereby enabling *in vivo* protein labeling.

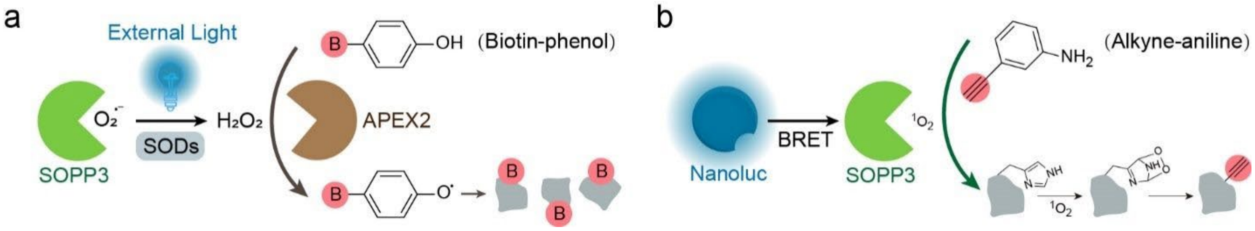

Rebuttal Figure 5. The labeling mechanisms of APEX2-SOPP3 (a) and BRET-ID (b).

2. The authors make a significant claim that BRET-ID eliminates the need for spatial references in proximity labeling experiments. Additional discussion is needed about the contexts where this applies, or if they want to generalize this claim, at least one more compartment dataset supporting this assertion would be beneficial. It's unclear if the stress granule data also supports this claim, and this should be addressed. Maybe by using a similar ROC curve analysis.

Response: We sincerely thank the reviewer for raising this important point. BRET-ID's ability to operate without spatial reference comparison is indeed strongly supported by our stress granule data, which we previously included as **Figure S6e** in the original manuscript. As detailed in this analysis, spatial reference comparison not only failed to improve specificity in SG identification but also reduced sensitivity—a pattern aligning with our earlier observations during ER membrane mapping experiments. Based on these findings, we omitted spatial reference comparison when compiling the final SG protein lists. The resulting list demonstrates exceptional specificity for SG-associated proteins, as shown in **Figure 5**.

Minor Concerns

1. I would recommend putting the current fig 1 into SI. NLuc-Halotag seems like an inferior design compared to NLuc-LOV/SOPP3. I would try not to confuse the readers and have them focus on the better design.

Response: We sincerely appreciate the reviewer's insightful comment. In response, we have relocated all experimental results originally presented in **Figure 1** to **Figure S1** in the revised manuscript, while retaining the comparative schematic diagrams illustrating blue light-based versus BRET-based photocatalytic labeling mechanisms in **Figure 1**. This reorganization improves manuscript clarity by separating methodological frameworks from

detailed results, ensuring readers can focus on the conceptual distinction between these labeling strategies.

2. *Fig 1f, lane 1 from the left: how could a no-enzyme control have HA staining?*

Response: We sincerely appreciate the reviewer for highlighting this oversight. We deeply regret the labeling error that occurred in our original submission. Upon re-examining our laboratory records, we identified that the lane in question was inadvertently mislabeled during data organization by one author, and this discrepancy was unfortunately overlooked by all authors, including the corresponding author, during final verification. The sample in this lane corresponds to HA-cpNluc-HT transfection without exposure to either HMME or chloroalkane-HMME. We confirm that the experimental results themselves remain valid and reproducible, as this protocol has been rigorously repeated with consistent outcomes. The labeling error has been corrected in the revised manuscript, and we have implemented additional cross-checking protocols to prevent such oversights in the whole manuscript.

3. *Fig S2e left panel, why is BRET with enzyme weaker than no enzyme control?*

Response: We apologize for the unclear labels. The no enzyme control sample (the 3rd lane) is performed with blue light irradiation rather than under BRET. Therefore, the endogenous photosensitizers generate quite high labeling background with alkyne-aniline and blue light treatments. The no enzyme control for BRET-ID labeling has been shown in **Figure 2d**, which shows much lower labeling than the BRET-ID labeling sample. We have revise the labels of **Figure S2e** in the revise manuscript to make it clearer.

4. *In Figure 2, the optimization process for NanoLuc-SOPP3 linker design is described, but the rationale for choosing the rigid 15aa linker needs more detail.*

Response: We than the reviewer for the suggestion. We therefore added a more detailed description of the rationale for choosing the rigid 15aa linker in the revised manuscript. Our systematic optimization identified NanoLuc-SOPP3—a fusion protein of NanoLuc and SOPP3 linked by a rigid 15-amino-acid linker—as the most efficient BRET-ID version (**Figure 2c and S2a-b**). This rigid linker might stably maintain an optimal donor-acceptor distance, a critical parameter for efficient energy transfer. Based on these findings, this construct served as our lead candidate for subsequent characterization.

5. *Supplementary Figure 2c compares alkyne-aniline vs. alkyne-phenol probes, but lacks a detailed explanation for the superior performance of alkyne-aniline, which appears to be critical for the success of BRET-ID without APEX2.*

Response: We sincerely appreciate the reviewer's suggestion. To enhance mechanistic clarity, we have expanded the description of **Figure S2c** in the revised manuscript, explicitly

demonstrating that alkyne-aniline exhibits superior efficiency in labeling proteins oxidized by SOPP3-generated singlet oxygen ($^1O_2$) compared to alkyne-phenol.

6. *I recommend showing colocalization of V5 and biotin in Fig 2i similar to Fig S2h to demonstrate the better spatial resolution of BRET-ID.*

Response: We thank the reviewer for the suggestion. As suggested, we have included the corresponding colocalization graphs in **Figure 2i** of the revised manuscript.

7. *Page 17, line 47/48, reference superscript.*

Response: We thank the reviewer for the careful check. We have corrected it.

8. *For the tumor labeling experiment, it would be great to show a cross section of the tumor postlabeling between two groups to demonstrate the penetration of substrates and the coverage of the tissue.*

Response: We sincerely appreciate the reviewer's insightful suggestion. As recommended, crosssectional imaging has been incorporated into the revised manuscript. Due to furimazine's intrinsic pale-yellow color, we observed that tumor slices from furimazine-treated samples exhibit a distinct yellow coloration (**Rebuttal Figure 6a, now Figure S7a in the revised manuscript**), providing direct evidence of robust furimazine penetration into tumor tissue. This aligns with prior reports of furimazine's favorable tissue permeability in tumor models[2]. To further validate labeling efficacy, tumor slices were click-labeled with azide-biotin and subjected to streptavidin-AF647 immunofluorescence imaging. Pronounced furimazine-dependent biotinylation was observed throughout the tumor tissue (**Rebuttal Figure 6b, now Figure S7b in the revised manuscript**), demonstrating the depth and uniformity of BRET-ID labeling within tumors.

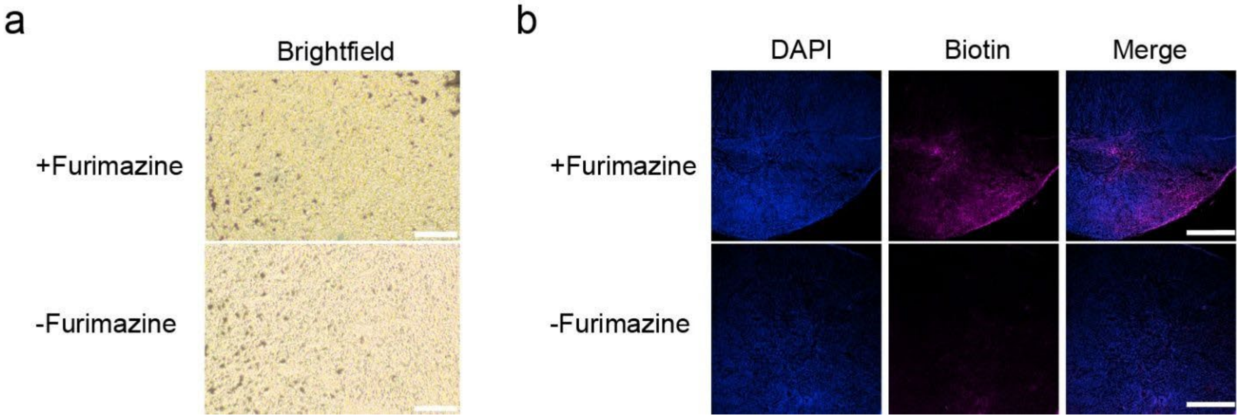

Rebuttal Figure 6. Cross-section imaging of furimazine-treated tumors. (**a**) Tumor samples were embedded, cryosectioned into 10-μm-thick slices, and mounted onto glass slides for brightfield imaging. (**b**) Immunofluorescence analysis of BRET-ID labeling in tumor tissues.

Tissue sections underwent heat-mediated antigen retrieval, followed by click-labeling with azide-biotin. Staining with DAPI (nuclei) and streptavidin-AF647 (labeling signals) was performed to visualize spatial biotinylation patterns. Scale bars, 200 μm.

Overall, this work represents a significant advance in proximity labeling technology with particular value for in vivo applications. With appropriate revisions addressing the above-mentioned comments, this manuscript would merit publication in ACS Central Science.

Response: We thank the reviewer again for the positive comments.

Reviewer: 3

Recommendation: Major revisions required.

Comments:

This manuscript presents an innovative and timely proximity labeling platform, BRET-ID, which combines genetic encoding, oxidative precision, and in vivo compatibility without reliance on exogenous light or hydrogen peroxide. The authors successfully demonstrate labeling in diverse compartments and deliver the first proteomic map of stress granules in live tumor tissue. The study is compelling in scope, carefully executed, and clearly written. For the publication of this manuscript in ACS Central Science, I recommend the following revisions:

Response: We thank the reviewer for the positive comments.

Major comments

1. A key promise of BRET-ID is its broad applicability to organelles. However, labeling was not tested in the mitochondrial matrix, which poses a unique challenge due to its compartmentalization and the need for furimazine to cross the inner mitochondrial membrane. A matrix-targeted NanoLuc-SOPP3 construct should be tested to determine whether BRET-ID is functional in this topologically enclosed environment.
Even a negative result would be informative and help define the method's boundaries.

Response: We thank the reviewer for this insightful suggestion. Following your recommendation, we engineered a construct fusing NanoLuc-SOPP3 to a mitochondrial targeting sequence and transfected it into HEK293T cells to perform BRET-ID labeling. Confocal imaging confirmed that the mitochondria matrix-targeted NanoLuc-SOPP3 co-localizes with citrate synthase (a mitochondrial marker), and the BRET-ID labeling signal

post furimazine addition aligns with both the enzyme and the marker (**Rebuttal Figure 7; Figure 2i in the revised manuscript**). These findings demonstrate that furimazine can permeate the inner mitochondrial membrane, enabling BRET-ID functionality within this topologically enclosed compartment. This observation aligns with prior studies showing NanoLuc activity in mitochondria upon furimazine administration, such as the established MitoLuc assay system[3].

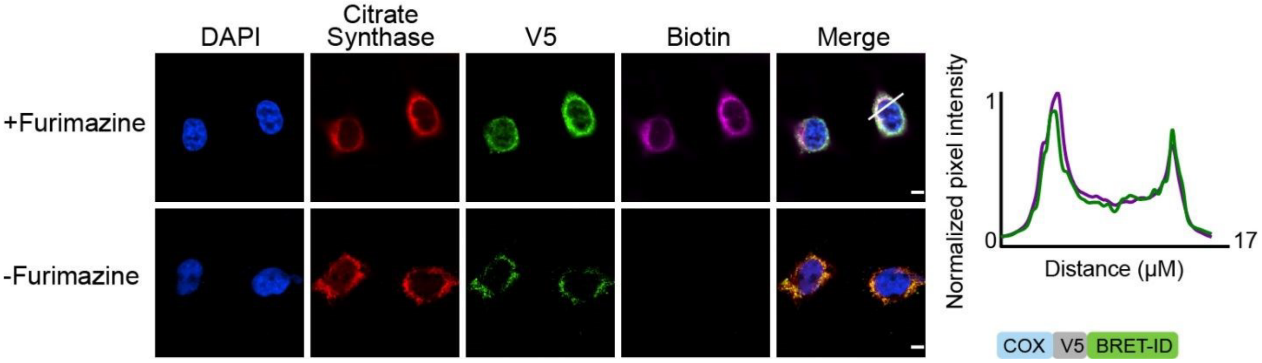

Rebuttal Figure 7. Confocal fluorescence imaging of BRET-ID labeling in the mitochondrial matrix. HEK293T cells were transfected with the mitochondrial matrix-targeted BRET-ID construct for 24 hours, followed by furimazine-based labeling initiation. Cells were fixed and labeled with azide-biotin, followed by staining with Streptavidin-AF647 for visualization of labeled proteins. Anti-V5 staining indicates enzyme expression. Citrate Synthase marks the mitochondria. Scale bars, 5 μm. White lines indicate where line plots were generated. Average intensity of biotinylation and V5 staining was quantified.

2. In the tumor xenograft model, proximity labeling by G3BP1-BRET-ID was analyzed without an in vivo cytosolic reference control. Including a tumor-expressed cytosolic BRET-ID control would help establish the specificity of the G3BP1 interactome and distinguish it from background cytosolic labeling, especially under tumor stress conditions. This control would mirror the in vitro controls and improve confidence in the in vivo interactome conclusions.

Response: We sincerely appreciate the reviewer's insightful observation. BRET-ID's capacity to function without spatial reference comparison is empirically validated by our ER membrane (ERM) and stress granule (SG) datasets. As demonstrated in these analyses, spatial reference comparison not only failed to enhance specificity in SG identification but also compromised sensitivity. Consequently, we deliberately excluded this step during the compilation of SG protein lists in our original cell culture experiments. The final catalog exhibits remarkable specificity for SG-associated proteins, as rigorously validated in **Figure 5**. This rationale underpins our decision to omit cytosolic BRET-ID as a spatial reference for *in vivo* mapping. Critically, the tumor-derived *in vivo* dataset validates precise SG protein labeling and remains directly comparable to cell culture controls through application of identical analytical filters.

3. The manuscript uses different enrichment ratio thresholds (e.g., ratio >1 vs. >2) to define significantly labeled proteins across experiments, but the rationale behind these choices is unclear. Since these cutoffs influence the composition and interpretation of reported interactomes (e.g., for G3BP1 in vitro vs. tumor), I recommend that the authors briefly justify the threshold used in each context and whether these were empirically determined based on signal-to-noise or statistical robustness.

Response: We sincerely appreciate the reviewer's insightful observation. For our initial proof-ofconcept ERM proteomic experiments, we employed unbiased statistical analysis and applied a standard filtering criteria of fold change>1 and *p*-value<0.05 to define enriched proteins. This is why we select this cutoff for the first proof-of-concept proteomic experiment on ERM. In the SG proteomic experiments, however, the 5-minute BRET-ID labeling protocol (versus 1 minute for ERM) resulted in substantially higher overall protein enrichment. To account for this increased signal-to-noise ratio, we applied a more stringent threshold (fold change>2 and *p*-value<0.05) for SG data analysis. As suggested, we now explicitly clarify this rationale in the revised manuscript within the SG experimental context. Importantly, the same threshold (fold change>2 and *p*value<0.05) was applied to tumor-derived SG datasets, ensuring direct comparability between *in vitro* and *in vivo* results.

To further validate our selection of distinct cutoff ratios, we employed receiver operating characteristic (ROC) analysis—a standard methodology in proximity labeling studies for determining enrichment thresholds by optimizing the balance between true positive rate (TPR) and false positive rate (FPR). For BRET-ID-ERM labeling (the ROC curve shown in original manuscript **Figure 3c**), the ROC-derived cutoff ratio of 1.11 was identified as the value maximizing the TPR-FPR differential (**Rebuttal Figure 8a**). Notably, this threshold aligns closely with our empirically applied cutoff (fold change>1 and *p*-value<0.05), as the lowest enrichment ratio among proteins meeting these criteria is 1.01. In the SG cell culture experiments
(the ROC curve shown in original manuscript **Figure S6d**), the ROC analysis yielded a cutoff of 1.9 (**Rebuttal Figure 8b**). However, our actual applied threshold (fold change>2 and *p*value<0.05) adopts a more stringent selectivity to ensure high-confidence identifications. Collectively, these ROC-driven analyses rigorously substantiate our cutoff selections and underscore the reliability of our final protein lists.

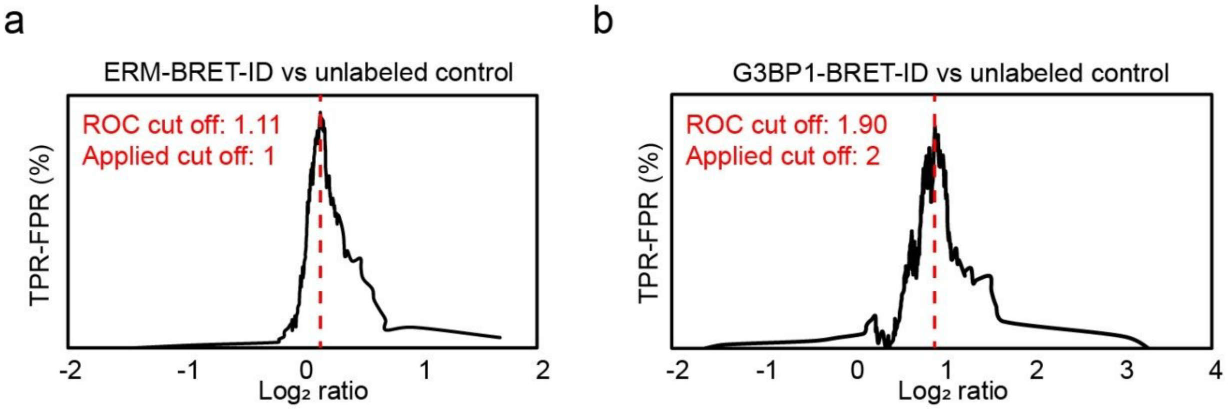

Rebuttal Figure 8. ROC cutoff ratios determined by maximizing the TPR-FPR differential for ERM (a) and SG (b) datasets.

Minor comments

4. Figure 2D includes an alkyne-phenol structure, which does not appear to be used in the study. Since the text and data focus exclusively on alkyne-aniline, I suggest removing the phenol structure to prevent confusion unless experimental data using it are presented.

Response: We thank the reviewer for the suggestion. We have removed the alkyne-phenol structure from **Figure 2**.

5. The authors may consider mentioning the iAPEX method (Sroka et al., bioRxiv, 2025), which also enables in vivo oxidative labeling by local hydrogen peroxide generation via co-expresion of d-amino acid oxidase. Although mechanistically distinct, both systems share a goal of enabling safe, precise proximity labeling in live organisms. A short reference in the Discussion would help readers situate BRET-ID within the broader context of emerging in vivo PL platforms.

Response: We thank the reviewer for this suggestion. In alignment with feedback from Reviewer 2, we have incorporated a detailed discussion of BRET-ID and iAPEX methodologies in the revised manuscript. This addition clarifies their distinct advantages and mechanistic differences to enhance reader understanding.

Reference:

1. Benavides-Serrato, A.; Lee, J.; Holmes, B.; Landon, K. A.; Bashir, T.; Jung, M. E.; Lichtenstein, A.; Gera, J., Correction: Specific blockade of Rictor-mTOR association inhibits mTORC2 activity and is cytotoxic in glioblastoma. *PLoS ONE* **2019,** *14* (2), e0212160.
2. Stacer, A. C.; Nyati, S.; Moudgil, P.; Iyengar, R.; Luker, K. E.; Rehemtulla, A.; Luker, G. D., NanoLuc reporter for dual luciferase imaging in living animals. *Mol. Imaging* **2013,** *12* (7), 1-13.
3. Needs, H. I.; Lorriman, J. S.; Pereira, G. C.; Henley, J. M.; Collinson, I., The MitoLuc Assay System for Accurate Real-Time Monitoring of Mitochondrial Protein Import Within Mammalian Cells. *J. Mol. Biol.* **2023,** *435* (13), 168129.

oc-2025-005209.R2

Name: Peer Review Information for "Precise and in vivo-compatible spatial proteomics via bioluminescence-triggered photocatalytic proximity labeling"

Second Round of Reviewer Comments

Reviewer: 2

Comments to the Author

All comments addressed satisfactorily. I would just add V5 staining in Fig S7b if possible.

Reviewer: 3

Comments to the Author

During the revision process, the authors have made improvements to their manuscript. However, there are still remained issues that need to be addressed before the manuscript can be considered for publication:

1. The proximity labeling radius of the alkyne-aniline radical remains insufficiently characterized, making it difficult to accept the assertion that this approach achieves

spatial resolution precise enough to eliminate the need for cytosolic or organelle reference datasets. The authors suggest that their ROC analyses of ERM and SG data demonstrate that reference data are unnecessary for defining the spatial proteomes of these compartments. However, I remain unconvinced by this argument. For example, Table S1 (ERM mapping data) lists among the top-ranked proteins APOO, TIMM13, SFXN2, and UQCC5 — all well-established residents of the mitochondrial intermembrane space or inner mitochondrial membrane. These proteins cannot reasonably be considered ERM components. While the authors might contend that these mitochondrial proteins were labeled due to close apposition of mitochondria and ERM, this would further emphasize the need for reference datasets (e.g., from mitochondria or cytosol) to accurately resolve spatial proteomes. As an illustration, if the authors performed BRET-ID mapping using constructs targeted to the outer or inner mitochondrial membranes, such apparent contaminants could be identified and removed.

Furthermore, the rationale regarding the observed decrease in specificity when comparing against untargeted BRET-ID controls is not clearly explained. The reduction in specificity may indicate that the labeling radius of alkyne-aniline radicals from the untargeted BRET-ID construct is broad enough to capture resident proteins of ERM or SG. I recommend that the authors explicitly discuss this potential limitation and revise their conclusions regarding the sufficiency of non-referenced datasets accordingly. If the authors continue to argue that comparison with untargeted controls is less informative than comparison with unlabeled controls, a clear rationale should be provided in the revised manuscript

2. In accordance with standard proteomics research guidelines, the raw mass spectrometry data generated and analyzed in this study should be deposited in a publicly accessible proteomics repository, such as PRIDE or ProteomeXchange. The corresponding accession number or link should be provided, ideally in the Acknowledgments section.

Reviewer: 1

Comments to the Author

The authors have addressed all of my previous concerns. Congratulations on this work!

Author's Response to Peer Review Comments:

Reviewer: 2

Recommendation: Publish in ACS Central Science without change.

Comments:

All comments addressed satisfactorily. I would just add V5 staining in Fig S7b if possible.

Responses: We thank the Reviewer for their positive feedback. Regarding Figure S7b, because both the -furimazine and +furimazine conditions express the V5-tagged enzyme, we did not include V5 staining during last revision. We suggest that biotinylation staining alone sufficiently demonstrates the depth of BRET-ID labeling within tumor tissues, as it directly visualizes the labeling event rather than enzyme expression.

Reviewer: 3

Recommendation: Publish in ACS Central Science after minor revisions noted.

Comments:

During the revision process, the authors have made improvements to their manuscript. However, there are still remained issues that need to be addressed before the manuscript can be considered for publication:

1. The proximity labeling radius of the alkyne-aniline radical remains insufficiently characterized, making it difficult to accept the assertion that this approach achieves spatial resolution precise enough to eliminate the need for cytosolic or organelle reference datasets. The authors suggest that their ROC analyses of ERM and SG data demonstrate that reference data are unnecessary for defining the spatial proteomes of these compartments. However, I remain unconvinced by this argument. For example, Table S1 (ERM mapping data) lists among the top-ranked proteins APOO, TIMM13, SFXN2, and UQCC5 — all well-established residents of the mitochondrial intermembrane space or inner mitochondrial membrane. These proteins cannot reasonably be considered ERM components. While the authors might contend that these mitochondrial proteins were labeled due to close apposition of mitochondria and ERM, this would further emphasize the need for reference datasets (e.g., from mitochondria or cytosol) to

accurately resolve spatial proteomes. As an illustration, if the authors performed BRET-ID mapping using constructs targeted to the outer or inner mitochondrial membranes, such apparent contaminants could be identified and removed.

Responses: We thank the Reviewer for their insightful comments. We agree that the assertion of eliminating the need for a spatial reference was overstated. Consequently, we have moderated this claim in the revised manuscript.

We appreciate the Reviewer identifying the enrichment of mitochondrial proteins in the ERMtargeted BRET-ID dataset. This finding is actually well-anticipated. As the Reviewer suggests, it aligns with observations that ERM-targeted and outer mitochondrial membrane (OMM)-targeted APEX2 enzymes cross-label each other's resident proteins, even when using a cytosolic APEX2 spatial reference[1]. This phenomenon occurs because the ER and mitochondria form close membrane contact sites (MCSs) at distances of approximately 30 nm[2]. Proteins residing within this proximity range are physiologically relevant neighbors and inherently challenging to resolve distinctly using proximity labeling techniques.

We acknowledge the Reviewer's suggestion that using a mitochondria-resident spatial reference could help mitigate these mitochondrial signals. However, such a reference would simultaneously label many genuine ERM proteins, resulting in the undesirable removal of true positives. Therefore, spatial reference filtering represents a persistent trade-off in proximity labeling: it enhances the specificity of the final protein list at the expense of sensitivity.

In direct response to these comments, we have removed all claims stating "no need for spatial references." Instead, we now simply report our observation that employing a spatial reference did not significantly enhance the specificity of the BRET-ID-generated ERM dataset while simultaneously reducing the number of proteins identified.

Furthermore, the rationale regarding the observed decrease in specificity when comparing against untargeted BRET-ID controls is not clearly explained. The reduction in specificity may indicate that the labeling radius of alkyne-aniline radicals from the untargeted BRET-ID construct is broad enough to capture resident proteins of ERM or SG. I recommend that the authors explicitly discuss this potential limitation and revise their conclusions regarding the sufficiency of non-referenced datasets accordingly. If the authors continue to argue that comparison with untargeted controls is less informative than comparison with unlabeled controls, a clear rationale should be provided in the revised manuscript

Responses: We thank the Reviewer for their comments. We acknowledge that the original manuscript did not explicitly mention that comparison against the untargeted BRET-ID control reduces specificity, and we apologize for any confusion this may have caused. To

address this, we have added a new figure panel (Rebuttal Figure 1; now Figure S4d in the revised manuscript) demonstrating the specificity of the BRET-ID-ERM dataset before and after filtering against the spatial reference. Following this filtering step, the final list retained only 77 proteins, with many known ERM proteins also being excluded (Rebuttal Figure 1a). Crucially, the filtering did not significantly improve the specificity of the ERM labeling. Proteins identified before and after spatial reference filtering showed comparable subcellular distributions (Rebuttal Figure 1b, now Figure S4e in the revised manuscript). This stands in stark contrast to blue light-based labeling, where spatial reference filtering markedly enhances ERM specificity.

Given that the BRET-ID-ERM dataset prior to spatial reference comparison already exhibits high specificity, we have used this larger list for subsequent analyses. As noted in our previous response, we no longer claim spatial reference filtering is unnecessary. Instead, we clarify for users that this step represents a trade-off between sensitivity and specificity.

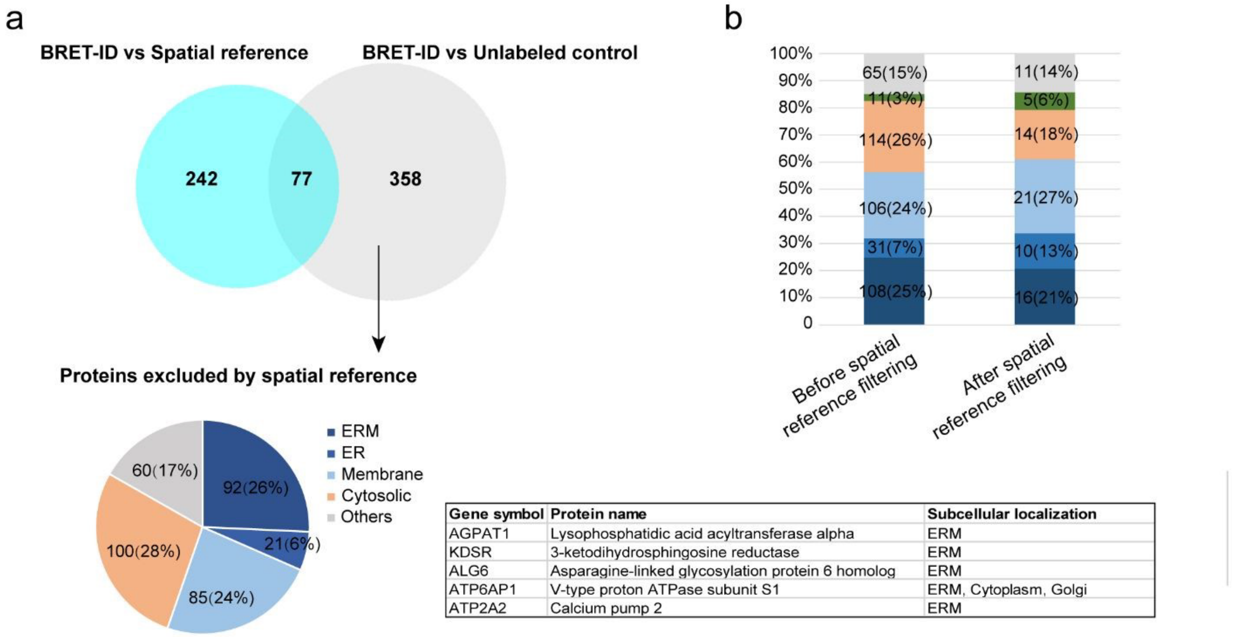

Gene symbol	Protein name	Subcellular localization
AGPAT1	Lysophosphatidic acid acyltransferase alpha	ERM
KDSR	3-ketodihydrosphingosine reductase	ERM
ALG6	Asparagine-linked glycosylation protein 6 homolog	ERM
ATP6AP1	V-type proton ATPase subunit S1	ERM, Cytoplasm, Golgi
ATP2A2	Calcium pump 2	ERM

Rebuttal Figure 1. Spatial reference filtering decreases sensitivity without significantly improving specificity in BRET-ID-ERM. (**a**) Subcellular distribution of the 358 BRET-IDenriched proteins excluded by spatial reference filtering. Representative ER-related proteins excluded by filtering are shown, along with their UniProt-annotated subcellular localizations. (**b**) Subcellular distribution of BRET-ID-enriched proteins before versus after spatial reference filtering.

2. In accordance with standard proteomics research guidelines, the raw mass spectrometry data generated and analyzed in this study should be deposited in a publicly accessible proteomics repository, such as PRIDE or ProteomeXchange. The corresponding accession number or link should be provided, ideally in the Acknowledgments section.

Responses: We thank the reviewer for pointing this out. We have submitted the raw mass spectrometry data to the iProX database; it can be accessed via the following links:

Link	Code
https://www.iprox.cn/page/SSV024.html;url=1751625964203Zdow	G8K7
https://www.iprox.cn/page/SSV024.html;url=1751626109679KjJl	sOo2
https://www.iprox.cn/page/SSV024.html;url=1751626141363siSb	imHs
https://www.iprox.cn/page/SSV024.html;url=1751626163113KD2Q	JgRb
https://www.iprox.cn/page/SSV024.html;url=175162618460191rc	R1sj
https://www.iprox.cn/page/SSV024.html;url=1751626205265QDzU	3NO4

The accession link is included in the Acknowledgments section. These data will become publicly available upon the manuscript's publication.

Reviewer: 1

Recommendation: Publish in ACS Central Science without change.

Comments:

The authors have addressed all of my previous concerns. Congratulations on this work!

Responses: We thank the Reviewer for the supportive comments.

1. Hung, V.; Lam, S. S.; Udeshi, N. D.; Svinkina, T.; Guzman, G.; Mootha, V. K.; Carr, S. A.; Ting, A. Y., Proteomic mapping of cytosol-facing outer mitochondrial and ER membranes in living human cells by proximity biotinylation. *eLife* **2017,** *6*.
2. Cho, K. F.; Branon, T. C.; Rajeev, S.; Svinkina, T.; Udeshi, N. D.; Thoudam, T.; Kwak, C.; Rhee, H. W.; Lee, I. K.; Carr, S. A.; Ting, A. Y., Split-TurboID enables contact-dependent proximity labeling in cells. *Proc. Natl. Acad. Sci. U. S. A.* **2020,** *117* (22), 12143-12154.
